# Recent advances in mono- and multi-nuclear photoluminescent Cu(i) complexes with nitrogen containing ligands and their stimuli responsiveness†

**DOI:** 10.1039/d5sc04685h

**Published:** 2025-10-17

**Authors:** Alessandra Forni, Daniele Malpicci, Elena Lucenti, Luca Zecchinello, Alessia Colombo, Elena Cariati

**Affiliations:** a Institute of Chemical Sciences and Technologies ‘‘Giulio Natta’’ (SCITEC) of CNR Via Golgi 19 20133 Milano Italy; b Department of Chemistry, Università degli Studi di Milano Via Golgi 19 20133 Milano Italy daniele.malpicci@unimi.it elena.cariati@unimi.it

## Abstract

Luminescent Cu(i)-derivatives represent a highly desirable alternative to their noble metal analogues in view of copper's relative abundance and low environmental concerns while maintaining large color tunability, high quantum yield and low photo-thermal lability. Moreover, the large variety of structural forms, spanning from 0D mononuclear to 3D polynuclear compounds characterized by peculiar emissive features, opens the door to an incredibly huge family of Cu(i) derivatives with potential application in many different fields. The present review focuses on luminescent neutral 0D mono-, di-, tri- and tetranuclear Cu(i) complexes with N-donor ligands developed during the period 2020 to mid-2025. After a general overview, specific sections are dedicated to members of each nuclearity. Emphasis is given to compounds' stimuli responsiveness, in particular towards vapour exposure and thermal and mechanical perturbations.

## Abstract 2

Recently developed luminescent neutral 0D mono-, di-, tri- and tetranuclear Cu(i) complexes with N-donor ligands are gathered and analyzed with a focus on their synthesis, luminescent behavior and responsiveness to external stimuli.

## Graphical abstract

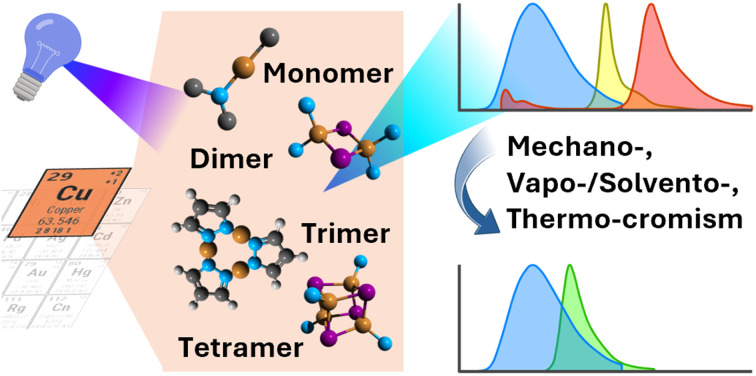

## 1. Introduction

The importance of photoluminescent materials in science and everyday life is testified by their widespread applications in diversified fields spanning from lighting and display technologies to bio-imaging.^1–4^ The search for new, highly efficient, low-cost luminescent materials is therefore strongly pursued and currently largely directed towards the development of earth-abundant, first-row transition metal derivatives as sustainable alternatives to precious metal photofunctional ones.^5^

In this regard, highly performing derivatives have already been obtained for the coinage Cu(i), Ag(i) and Au(i) series, where the d^10^ configuration guarantees the absence of detrimental d–d transitions.^6^ Inside this family, Cu(i) compounds have emerged as particularly intriguing not only over their more expensive Au(i) analogues with comparable photoluminescent performances but also over Ag(i) ones which are often characterized by lower stability towards air–oxygen mixtures and light leading to photodegradation.^7^ Moreover, Cu(i) systems display remarkable structural diversity often resulting in peculiar chemical and/or physical properties, which have already been exploited for organic light-emitting diodes (OLEDs),^7–11^ light-emitting electrochemical cells (LECs),^12^ solar cells,^13–16^ X-ray scintillators^17–19^ and luminescent sensors.^20,21^

**Fig. 1:**
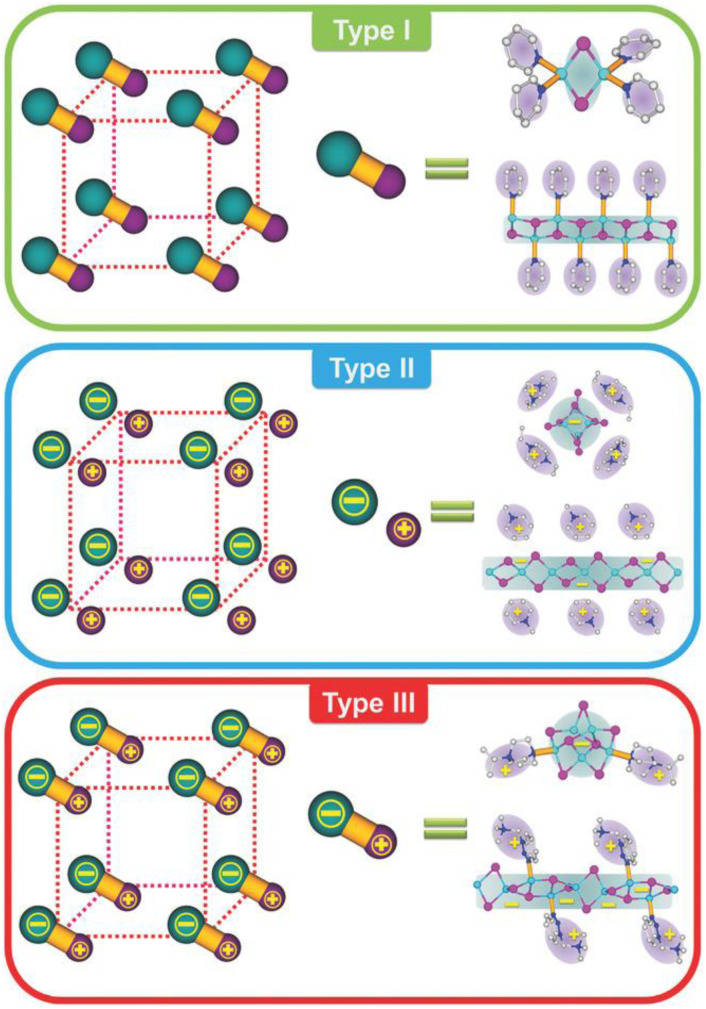
Conceptual representation (left; green ball: inorganic module; purple ball: organic ligand; yellow rod: coordinative bond) and examples (right) of Types I, II, and III structures. Type I structures are neutral CuX(L)_*n*_ species made of Cu–L dative bond only. Type II structures are ionic CuX(L) species composed of ionic bond only. Type III structures are AIO CuX(L) species possessing both bonds. Reproduced with permission from ref. 9. Copyright 2018, John Wiley and Sons.

Members of this family comprise both halogen free and halocuprate systems, with the latter conveniently classified^9^ into the following three main groups, based on the nature of the chemical interaction between Cu(i) and the organic ligand (typically containing nitrogen, sulfur or phosphorus): Type I structures are those formed through a dative copper–ligand bond; Type II are those where a cationic ligand interacts electrostatically with an anionic inorganic unit; in Type III structures, the halocuprate module and the ligand are connected through both dative and ionic bonds. Type I structures are generally more luminescent than Type II structures, which, on the other hand, are characterized by higher stability. The type III family, also denoted as all-in-one (AIO), synergistically combines the high stability of Type II structures with the fascinating photophysical properties of the Type I derivatives (Fig. 1).

Despite their promising features, the number of Cu(i) AIO systems is still limited, and relevant results have been collected in a recent review.^22^ Based on these considerations, here, we report on significant progress made on 0D neutral photoluminescent Cu(i) halocuprates of Type I and neutral halogen free coordination complexes during 2020–mid-2025. It is important to note that this focused selection inherently excludes certain noteworthy cationic mononuclear emitters, *e.g.* the family of [Cu(N^N)(P^P)]^+^ (where N^N = 2,2′-bpy derivatives and P^P = bulky chelating ligands, like tetraphenyldiphosphoxane (POP) or 4,5-bis(diphenylphosphino)-9,9-dimethylxanthene (Xantphos), which find successful application in LECs).^12^

**Fig. 2:**
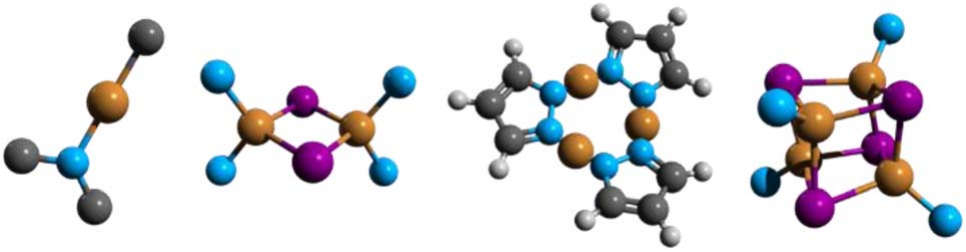
Schematic structures of the most representative monomers (labelled either 1.xy, with x being the NHC and y the amide ligands, or 1.*n*), dimers (labelled 2.*n*), trimers (labelled 3.*n*) and tetramers (labelled 4.*n*), where *n* is the compound number within a given category reported in the review. Colour code: brown = Cu; purple = halogen; light blue = nitrogen.

This review is organized in sections dedicated to monomers (numbered in the text as either 1.xy, where x stands for the N-heterocyclic carbene, NHC, and y for the amide ligands, or 1.*n*), dimers (2.*n*), trimers (3.*n*) and tetramers (4.*n*) (Fig. 2). These compounds are superior luminophores in the solid state with greater earth-abundancy and much cheaper prices as compared to late transition metal derivatives and, therefore, have gained ever-growing attention from both fundamental research and device construction. Among ligands supporting this family of complexes, we reviewed those including (but not limited to) the N-donor ones.

Remarkably, in the last few decades, the number of photoluminescent Cu(i) derivatives with response to grinding, temperature, exposure to vapours, stretching or pressing *etc.* has largely increased.^23,24^ Materials with stimuli responsive luminescence (variation in the position, intensity and/or lifetime) are of scientific and technological interest owing to their wide potential applications in several fields, including chemosensors, data storage, optoelectronic devices and imaging. Therefore, in the present review, particular emphasis on stimuli responsiveness of Cu(i) 0D neutral complexes is given. Because temperature-induced variations in luminescence are commonly encountered and typically addressed within photophysical characterization, thermochromic behavior will not be examined in a separate section. In contrast, vapochromism, mechanochromism and multi-stimuli responsiveness will be the subject of specific sections.

### 1.1 Overview of Cu(i) derivatives

Cu(i) derivatives are characterized by pseudo-tetrahedral, trigonal or linear coordination geometry around the metal center. Among these, tetra-coordinated complexes represent the majority. In these compounds, where d–d electronic transitions are avoided due to the Cu(i) d^10^ configuration, various excited states can serve as the emissive ones depending on the nature of the ligands and the specific structure.^5,6,25^ In neutral Cu(i) halocuprates of Type I and halogen free coordination complexes, the Cu(i)–L bond is essentially a dative bond of lone pairs of ligands to the vacant Cu(i) 4s and 4p orbitals; therefore, the highest occupied molecular orbital (HOMO) is mainly localized on the 3d orbitals of the metal center. In complexes with halogenides, the HOMO also contains contribution from the halogen coming from its π-interaction with the appropriate orbital on the metal. The lowest unoccupied molecular orbital (LUMO) is an antibonding orbital whose character strongly depends on the nature of the ligand.^21,26,27^ In complexes with aliphatic ligands, the LUMO is mainly composed of Cu(i) 4s and 4p orbitals, and the lowest excited state originates from a metal centered (MC) transition. In complexes with aromatic ligands having low energy π* orbitals, the LUMO possesses large ligand contribution resulting in the lowest energy excited state of metal to ligand charge transfer (MLCT) or mixed (M + X)LCT character (where X is a halogenide) and the emission color, for similar coordination environments, depending on the ligand.^26,28–30^ Since MLCT excited states can be regarded as a formal oxidation of Cu(i) to Cu(ii), they are characterized by a molecular reorganization from a pseudotetrahedral to a flattened Cu(ii) square-planar coordination, resulting in lowering of the excited-state energies and increase of non-radiative deactivations. Therefore, rigidification of the system hampering Jahn–Teller distortion exerts a positive effect on the quantum yield.^31^ Intriguingly, the small overlap between the orbitals involved in the transition is often reflected in a small energy difference between the singlet and triplet of MLCT nature. Energy gaps approximately below 10^3^ cm^−1^ (120 meV) are at the basis of the Thermally Activated Delayed Fluorescence (TADF) frequently observed for various Cu(i) complexes and exploited for singlet harvesting in OLED technology.^32,33^ Interestingly, some Cu(i) compounds have also been recognized as dual-mode emitters through concomitant TADF and phosphorescence at room temperature (r.t.), ensuring that both triplet and singlet harvesting are exploitable to improve the OLED performance.^34^

Moreover, ligand-centered (either intraligand, IL, or ligand-to-ligand, LL) excited states can also be responsible for the emission, especially when extended aromatic ligands are present.

In multinuclear compounds with Cu⋯Cu distances shorter than the sum of the van der Waals radii (2.8 Å or, as recently re-evaluated, 3.84 Å (ref. 35 and 36)), an additional metal centered emissive state can be due to the transition from the 3d orbital to vacant Cu⋯Cu overlapped 4s and 4p ones. To highlight its origin, such d–s,p transition, frequently admixed, in halogenido complexes, with a halide-to-metal charge transfer (XMCT) one, is indicated as “cluster-centered” (CC). The corresponding emission depends on Cu⋯Cu distances and geometrical distortions of the CC excited states and therefore reflects structural rigidity. As expected, while the ligand has a great impact on the (M + X)LCT band, it does not affect the CC one except for geometrical constraints. Moreover, possible intermolecular cuprophilic interactions can result in additional MM emissive states. Alteration of both intra- and intermolecular cuprophilic interactions by external perturbations is often at the basis of Cu(i) derivatives' stimuli responsiveness.

The preparation of Cu(i) complexes with nitrogen donor ligands can be accomplished through different synthetic approaches. A one pot reaction at r.t. or under solvothermal conditions, the use of either Cu(i) or Cu(ii) salts as starting materials and solid state reactions represent some possible routes. Particularly challenging in this regard is the establishment of the synthetic conditions to get halocuprate with N-donor ligands having the desired arrangement.^37^ In fact, due to the lability of the Cu–N bond and the easy interconversion between different almost isoenergetic isomeric forms, subtle changes, such as temperature, CuX : L ratio or solvent, and the steric requirements of the ligand, affect the nature of the molecular array often in a not predictable way. In some cases, mixtures of different derivatives can also be obtained. Therefore, while one pot synthesis from ligands and CuX in solution at r.t. represents a straightforward methodology for the preparation of homoleptic complexes, it lacks control over nuclearity. The latter problem can, in a few cases, be overcome by a ligand exchange strategy starting from a preformed complex stable in solution using an excess of the substituting ligand. When dealing with CuI derivatives, the use of a KI-saturated aqueous solution represents a possible alternative to CuI dissolved in an organic solvent (typically acetonitrile, ACN), again mostly leading to products with unpredictable nuclearity.

The one pot reaction can also be performed under solvent free or solvent assisted conditions by directly mixing the starting metal salts and the organic ligand(s) with the advantage of avoiding the use of large amounts of harmful and high-cost organic solvents. Despite the lower controllability of this approach with respect to a more traditional one in solution, ever growing examples of its application are reported in the literature due to its undoubted advantage from the viewpoint of practical applications for large-scale production.

## 2. Monomeric complexes

### 2.1 NHC-based linear monomers

Among the numerous photoluminescent neutral monomeric Cu(i) complexes, a great number have the general formula LCuL′, where Cu(i) is linearly coordinated, in most cases, to N-heterocyclic carbenes (NHC) and amides (mainly carbazolides). These carbene–metal–amide (CMA) complexes are easily prepared by deprotonation of carbazole, Cz, or its derivatives, with NaO^*t*^Bu in tetrahydrofuran (THF), followed by the addition of (NHC)CuCl. CMAs have emerged as efficient luminescent materials, exhibiting high photoluminescent quantum yields (*Φ*), luminescence decay lifetimes (*τ*) in the microsecond or shorter range and tuneable emission colours across the entire visible spectrum in solution, solid state and doped films.^38,39^

**Fig. 3:**
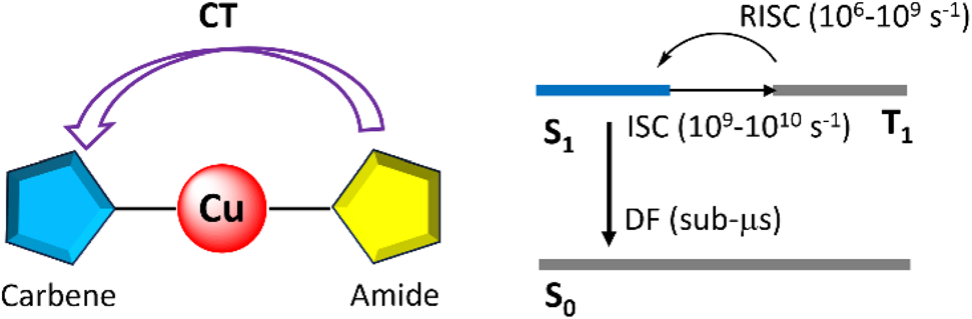
Diagram of excitation dynamics of CMA complexes; DF = delayed fluorescence.

Due to their copper-bridged linear geometry and coplanar conformation, CMAs are characterized by the localization of the HOMO on the amide and the LUMO on the carbene resulting in excited states of predominant ligand-to-ligand charge transfer (LLCT) character with minor metal contribution. The well-separated frontier molecular orbitals lead to small singlet–triplet energy gaps (Δ*E*_ST_), facilitating efficient TADF. Additionally, compared to purely organic TADF emitters, the presence of the copper centre enhances the SOC between singlet and triplet states, promoting rapid intersystem crossing (ISC) and reverse intersystem crossing (RISC), which result in short TADF lifetimes (in the sub-microsecond range, Fig. 3). The coplanar conformation further optimizes the electronic coupling between the ligands, increasing the oscillator strength and enabling CMAs to combine high radiative rates (*k*_r_) with short exciton lifetimes, making them highly promising for luminescent applications.

#### 2.1.1 Advancements during 2020–mid-2025

**Scheme 1:**
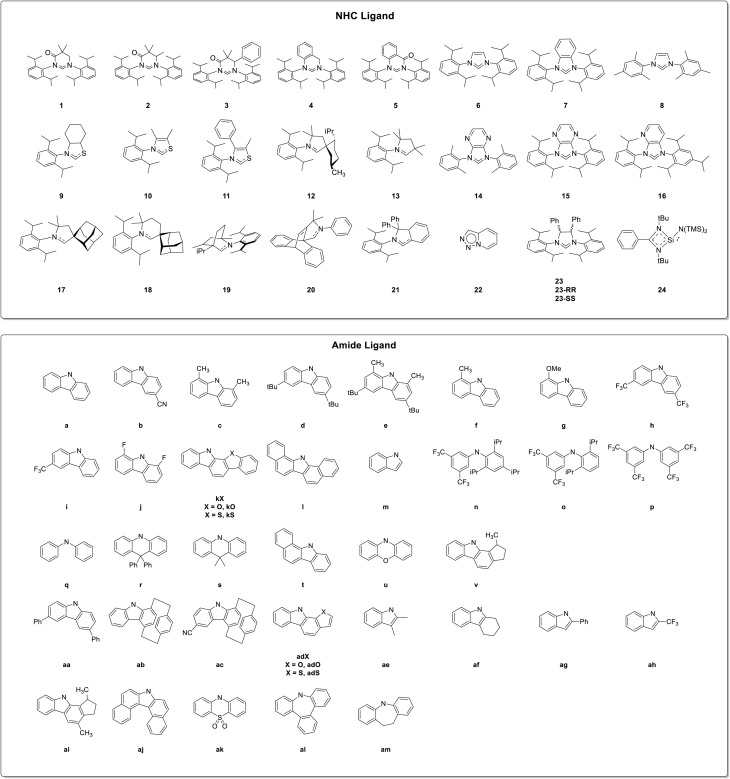
Structures of NHC and amide ligands.

In the present section, possible modifications of the NHC scaffold (Scheme 1 top) and the related effects on the electronic properties of the complexes are first considered. Successively, variation of the amide moieties are taken into account (Scheme 1 bottom). All derivatives that appeared in the literature in the 2020–mid-2025 period are collected in Table S1, where compounds are gathered according to the NHC ligand (even though reported in different publications), to better highlight the amide influence on the photophysical behavior. Moreover, CMA compounds are classified as 1.*n*x, where 1 stands for the monomer, *n* indicates the carbene type and the letter x refers to the amide.

**Fig. 4:**
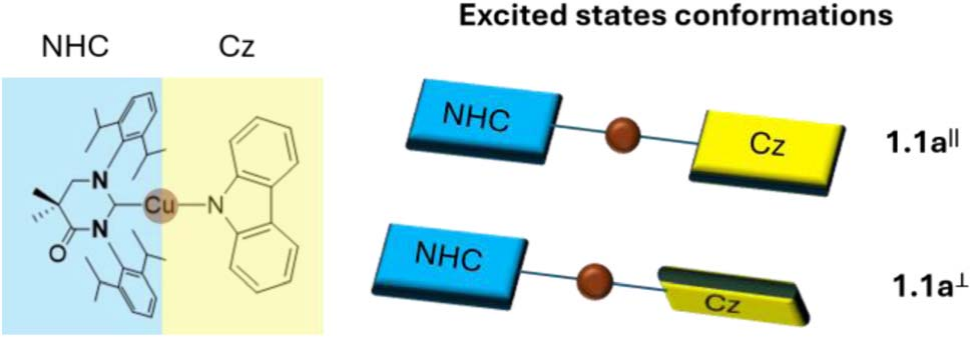
Left: molecular structure of 1.1a. Right: schematic representation of the excited state conformations with the two ligands coplanar (1.1a^‖^, top) or perpendicular to each other (1.1a^⊥^, bottom).

The mechanism behind TADF of a typical CMA complex, 1.1a (Fig. 4, left), was theoretically investigated employing the nuclear ensemble method.^40^ In agreement with previous results obtained on the same system through surface hopping nonadiabatic dynamics simulations, it was demonstrated that 1.1a displays two distinct excited-state conformations, having a coplanar or perpendicular orientation of the two ligands, each of them exhibiting unique excited-state dynamics (Fig. 4, right). In the gas phase, ISC from the S_1_ state proceeds through higher-lying triplet states, whereas RISC is observed exclusively in the perpendicular ligand orientation, occurring directly between the T_1_ and S_1_ states, both having mainly LLCT contribution. Moreover, the inclusion of solvent effects markedly alters the TADF mechanism, enabling RISC in both coplanar and perpendicular conformations. The calculated rate constants (4.05 × 10^7^ and 9.46 × 10^6^ s^−1^, respectively) show good agreement with the experimental one (1.20 × 10^7^ s^−1^).

Given the pivotal role of LLCT from the amide to the NHC ligand in influencing the emission wavelength and delayed fluorescence (DF) lifetimes of CMAs, a key approach for tailoring their optical properties is to adjust the π-accepting ability of NHC ligands, as highlighted by Ying and co-workers.^41^ A red-shift in the emission can be achieved by stabilizing the empty p-orbital of the carbene carbon atom and minimizing the antibonding contribution of the nitrogen atom in the LUMO. This can be done, for example, by promoting a greater sp^2^ character of the carbene carbon's p-orbital by incorporating a six-membered ring to increase the N–C_carbene_–N bond angle, approaching 120° (ligands 1–5) or, alternatively, replacing the nitrogen atom with a carbon or sulfur one (ligands 9–13). The introduction of electron-withdrawing carbonyl groups into the six-membered NHC backbone to stabilize its π* orbitals, resulting in a lowered LUMO energy,^18^ was exploited by Li *et al.*^42,43^ Complexes 1.2a,b and 1.3a,b display broad visible ILCT emissions in polystyrene (PS) films at r.t., with energies primarily governed by ligand substituents. The functionalization of the carbene scaffold with phenyl groups (1.3a,b) leads to a 25 nm red shift with respect to methylated analogues (1.2a,b) due to stabilization of both HOMO and LUMO levels.

In 2020, Chotard *et al.*^44^ prepared and investigated a family of CMAs based on six-membered mono (18)- or bi (19)-cyclic (alkyl)(amino)carbene (CAAC) ligands to highlight the effect of electronic (higher amphiphilicity of the carbene with respect to the previously reported five-membered CAAC 17 (ref. 45)) and structural factors (mono- *versus* bicyclic carbene) on the photoluminescence of CMA emitters. It was proven that rigidifying the carbene ligand to enforce a linear geometry in the excited state represents an effective strategy to suppress nonradiative decay, enabling the simultaneous achievement of near-unity *Φ* and sub-microsecond excited-state lifetimes. In particular, while toluene solutions of 1.19a display emission at 502 nm, with *τ* and *Φ* being equal to 1.3 μs and 100%, respectively, those of 1.18a, which possess greater conformational flexibility, are characterized by negligible CT emission under the same conditions (*Φ* = 3.6%).

In 2025, Riley *et al.*^46^ reported a series of CMAs featuring the (amino)barrelene carbene (CABC, 20) ligand. Cu(i) derivative, 1.20a, displays (in a 1% by weight PS matrix) bright yellow TADF as supported by temperature dependent photoluminescent behavior. It exhibits a high activation energy of 83 meV alongside a high radiative rate of 3.5 × 10^5^ s^−1^ and *Φ* equal to 65%. The emission peak at 565 nm is 60 nm red-shifted compared to the CAAC analogue with 17 (*ca.* 505 nm), confirming the increased π-accepting ability of this ligand.

The strategy of substituting a nitrogen atom with a sulfur one was applied by Ruduss *et al.*, in 2022,^47^ for a family of Cu(i) complexes (1.9a–c, 1.10a–c and 1.11a–e) containing 1,3-thiazoline carbenes. The developed compounds exhibit bright (*Φ* up to 86%) tunable emission, ranging from blue-green to green, through structural modifications of the carbazolide. The emissive mechanism is attributed to TADF, with *k*_r_ ranging from 2.8 × 10^5^ to 7.2 × 10^5^ s^−1^. Remarkably, 1,3-thiazoline-based ligands allow closer solid-state packing with respect to that observed for more hindered CMA complexes, allowing the formation of efficient electroluminescent excimers exploitable for the realization of OLED devices. In fact, acting on the steric hindrance of the emitter or on its concentration, it is possible to tune the prevalence of electroluminescence (EL) from either the monomer (bluish green) or the excimer (orange-red), resulting in a single-emitter white OLED (WOLED).

An alternative approach to stabilize the empty p-orbital of the carbene carbon is to expand the NHC π-system. This can be achieved by developing CMAs bearing phenyl-fused NHCs, a strategy that was employed in the design of 1.4a,^48^1.5a,^48^1.7a,^49^1.21a,^38^ and 1.22a ^50^ or by using pyrazine (Pyz)-/pyridine (Py)-fused NHCs as in 1.14a,^51^1.15a,^51,52^ and 1.16a.^52^1.14a, 1.15a and 1.16a exhibit TADF that is red-shifted with respect to the analogous benzo-fused 1.7a,^49^ due to the stronger electron-accepting ability of Pyz and Py, respectively. Remarkably, complex 1.21a, reported by Gernert *et al.*^38^ in 2020, is brightly luminescent in the red region (621 nm, *Φ* = 32%) and is a TADF emitter with a *k*_r_ of *ca.* 9 × 10^5^ s^−1^.

Focusing on the amide moiety, a widely adopted strategy to tune the LLCT transitions is its functionalization with either electron-donating (1.4a, 1.5a, 1.9c, 1.10c, 1.11c–e, and 1.12d–g ^53^) or electron-withdrawing (1.1h–i,^54^1.2b, 1.3b, 1.15b,^52^ and 1.17j ^55^) groups resulting, respectively, in red- or blue-shifted emission.

The optoelectronic properties of CMAs can also be modulated through intramolecular noncovalent interactions, as reported by Ying *et al.*^48,56^ The introduction of secondary metal–ligand contacts into linear Cu(i) complexes, *via* chalcogen-functionalized Cz-ligands, stabilizes favorable conformations and enhances emission properties. In particular, in complex 1.1kS, sulfur-based (S⋯Cu) interactions promote planar structures, resulting in high photoluminescence quantum yields (up to 93%) and short radiative lifetimes (as low as 0.8 μs). Zhang *et al.*^51^ reported complexes 1.14l and 1.15l where the Cz ligand forms noncovalent hydrogen bonding with the metal center in addition to the primary coordination σ-bond, resulting in a pincer-type chelation mode. 1.14l and 1.15l exhibit enhanced stability, strong TADF with a *Φ* up to 86% and short lifetimes (1.01 and 0.65 μs, respectively).

Another approach to shorten the DF lifetime is the use of indole derivatives, as proposed by Wang and co-workers.^57^ Indole-based CMA 1.1m, in fact, exhibits a shorter DF lifetime of 0.55 μs compared to its Cz-based counterpart 1.1a (1.4 μs), the two compounds having an almost identical excited-state energy (505 nm).

**Fig. 5:**
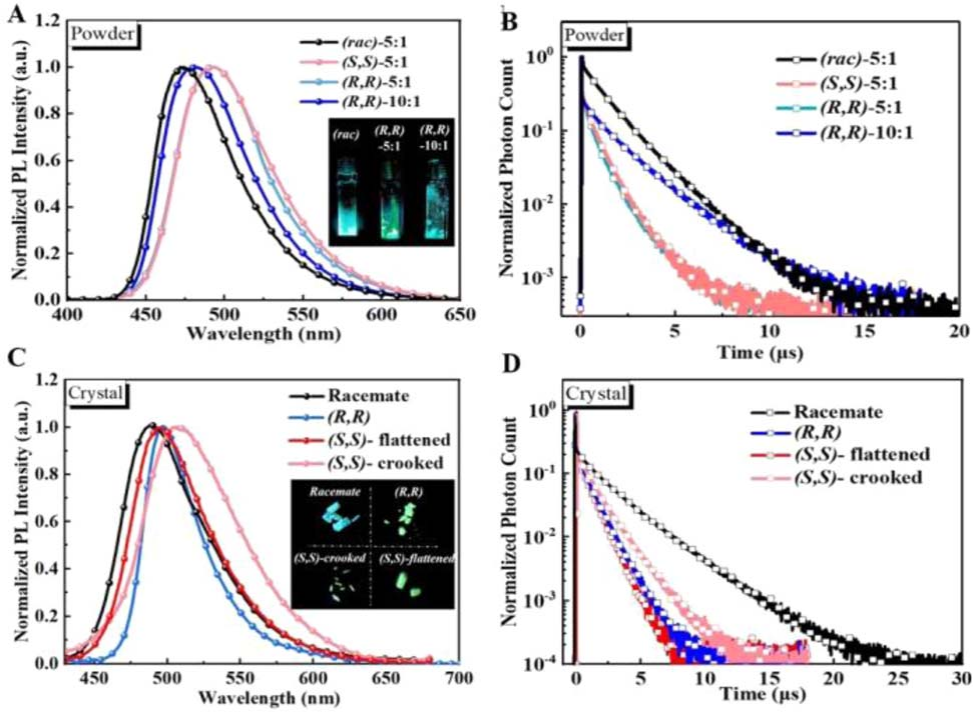
Fluorescent and transient photoluminescent decay curves of (*rac*)-, (*R*,*R*)- and (*S*,*S*)-1.23 (A and B) in powder form and (C and D) as single crystals (*λ*_exc_: 370 nm). Inset: powder and crystals under 365 nm UV light. Reproduced with permission from ref. 60. Copyright 2022, John Wiley and Sons.

To further enhance the ligand-to-ligand charge transfer, Ghosh *et al.*^58^ proposed variously substituted diphenyl amines, 1.6n–p,q. Similarly, acridine-based donors were employed by Ying *et al.*,^59,60^1.1r and 1.23s. Remarkably, 1.23s having a chiral NHC, represents the first chiroptically active CMA.^60^ It was demonstrated that the chiroptical properties are strongly influenced by the rotational freedom of the ligands. In a rigid environment, like powder and crystals, where the rotational freedom is limited, 1.23s, circularly polarized luminescence (CPL) is enhanced. Additionally, an interesting aggregation-dependent TADF was observed in both powder and single crystals. This effect was explained by the presence of several emissive CMA conformers with varying ligand–ligand dihedral angles (Fig. 5).

A family of CP-TADF CMAs displaying also mechanochromic features (see Section 6.2) was reported by Muthig *et al.*^53^ According to the rigidity of the environment, compounds 1.12a–u and 1.13v are either TADF (THF solutions) with high *k*_r_ (10^5^ s^−1^ order of magnitude) or mainly phosphorescent (solid state) emitters from LLCT/MLCT states. Furthermore, 1.12u, having phenoxazinyl (PZN) as the donor ligand, is the only compound of the series which displays, in PMMA, high CPL activity, besides very efficient orange-red TADF (*k*_r_ = 6.7 × 10^5^ s^−1^). This is related to the hindered ligand rotation in the rigid matrix in combination with a butterfly distortion of the PZN ligand, that is not observed for the carbazolate ligands.

Ghosh *et al.*^58^ reported a pioneering study on a family of N-heterocyclic silylene, NHSi, Cu(i) amide emitters (1.24n–q), employing a benzamidinato silylene ligand [PhC(^*t*^BuN)_2_SiN(TMS)_2_] in combination with acyclic secondary amines. The complexes were easily synthesized using a substituted diphenylamine along with the NHSi–copper mesityl complex in a 1 : 1 molar ratio in toluene at r.t. 1.24q displays in the solid state efficient TADF (*Φ* = 11%), as demonstrated by variable temperature photophysical characterization and supported by its calculated remarkably small singlet–triplet energy gap (Δ*E*_ST_ = 0.01 eV). The other members of the family feature dual emission (fluorescence and phosphorescence, modulated by the nature of the amide moiety) associated with their higher Δ*E*_ST_ (about 0.4 eV).

### 2.2 Tetrahedral and trigonal monomers

The use of rigid chelating ligands in combination with mutual sterical hindrances can minimize structural distortion and significantly enhance photoluminescence quantum yields, as reported in many publications that appeared during 2020–mid-2025 (Table S2).

#### 2.2.1 Tetrahedral compounds

**Scheme 2:**
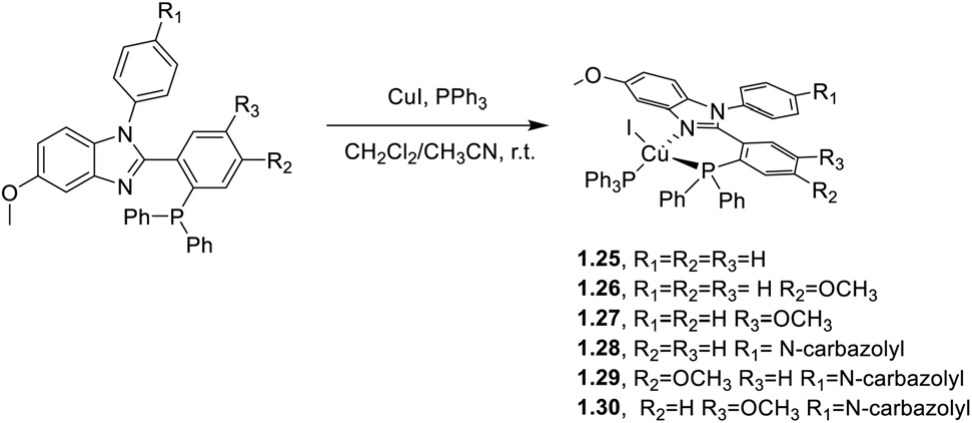
Synthesis of 1.25–30.

**Scheme 3:**
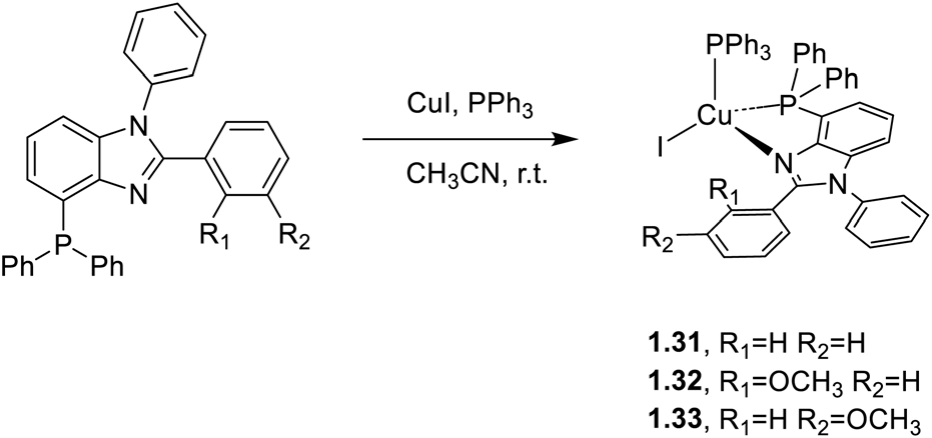
Synthetic procedure for 1.31–33.

In 2020 and 2021, Zhang and co-workers^61,62^ proposed a series of highly phosphorescent Cu(i) complexes of general formula [CuI(PPh_3_)(N^P)] containing either 2(2′-diphenyl-phosphinophenyl)-1-phenyl-5-methoxybenzimidazole derivatives (1.25–30)^61^ or 4-(diphenylphosphanyl)-1,2-diphenylbenzimidazole derivatives (1.31–33) as chelating N^P ligands.^62^ The compounds were prepared by a one pot reaction in solution, as reported in Schemes 2 and 3, respectively.

Powders of 1.25–30 display at r.t. high *Φ* (up to 88.3%), short emission decay times (from 4.8 to 55.2 μs) and emission maxima (in the 529–577 nm interval) tunable by varying the number and position of methoxy substituents on the N^P ligand.

Similarly, powders of 1.31–33 exhibit bright luminescence (*Φ* equal to 35% for 1.31, 27% for 1.32 and 30% for 1.33). Solution processed OLEDs with 1.31 and 1.33 as emissive dopants achieved the highest external quantum efficiencies, EQEs (3.08% for 1.33 and 3.00% for 1.31), with a brightness of 4410 and 4412 cd m^−2^, respectively. Instead, the brightest electroluminescence (7729 cd m^−2^) was reported for phosphor 1.29 as a dopant with 2.38% EQE.

**Scheme 4:**
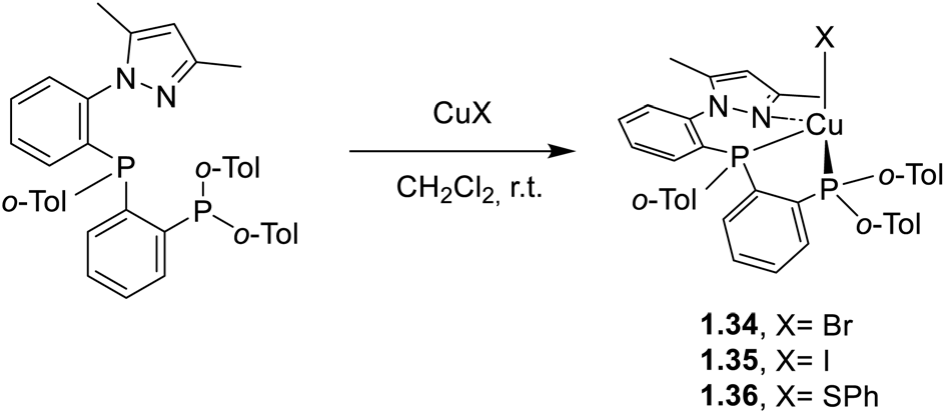
Synthesis of 1.34–36.

In parallel, Klein *et al.*^63^ investigated a novel rigid tridentate N^P^P ligand (namely 3,5-dimethyl-1-(2-((2-(di-*o*-tolyl)phosphanyl)(*o*-tolyl)phosphanyl)phenyl)-1*H*-pyrazole) in combination with Br^−^, I^−^ or SPh^−^ as monodentate ligands. Powders of compounds 1.34–36, prepared as reported in Scheme 4, demonstrated bright TADF (*Φ* up to 90%), as supported by the calculated small Δ*E*_ST_ (below 0.1 eV) and variable temperature photoluminescent investigations.

**Scheme 5:**
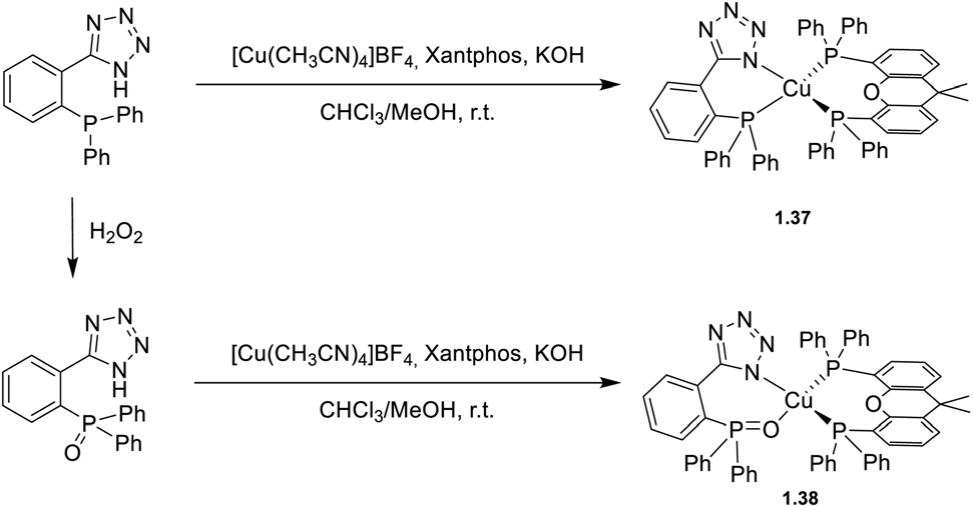
Synthesis of complexes 1.37 and 1.38.

In 2024, Cheng *et al.*^64^ developed an N^P chelating ligand to prepare rigid Cu(i) complexes (1.37 and 1.38, Scheme 5) with suppressed pseudo-Jahn–Teller distortion. 1.37 and 1.38, where the N^P ligand or its oxidized N^O form, respectively, is combined with Xantphos, exhibit in the solid state high *Φ* values (52 and 85%, respectively) and short emission lifetimes (4.2 and 7.5 μs, respectively) at r.t. According to DFT/TDDFT calculations and variable temperature experiments, a TADF mechanism was proposed.

**Scheme 6:**
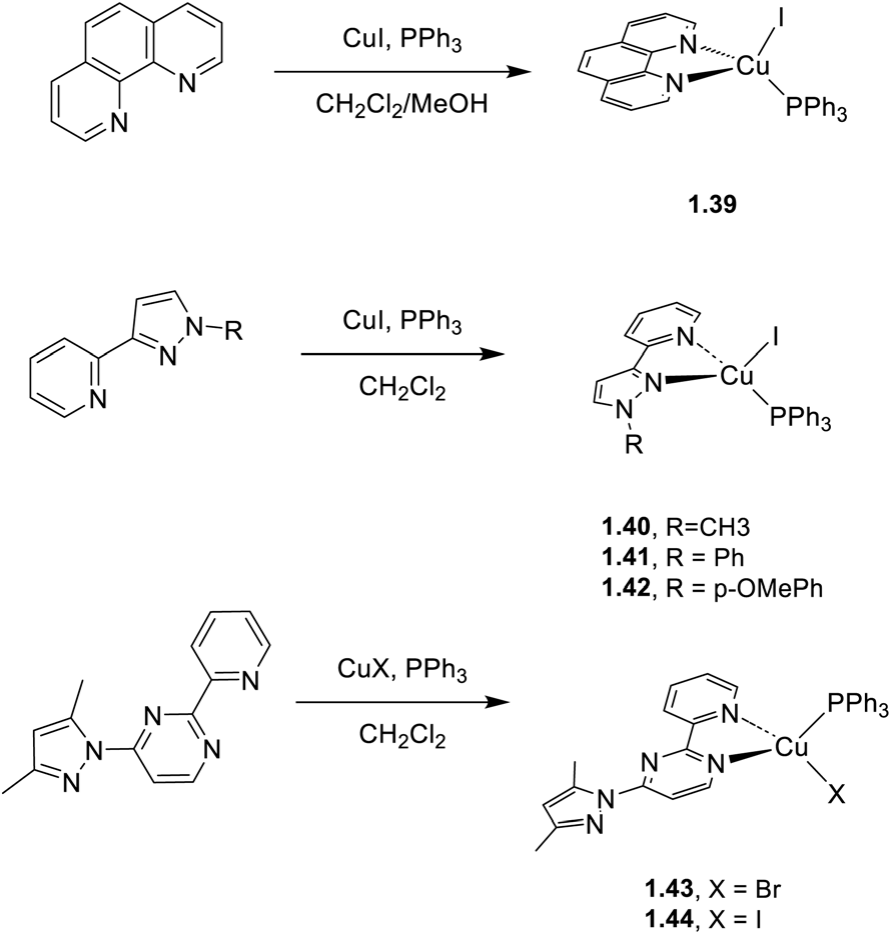
Synthesis of complexes 1.39, 1.40–42 and 1.43–44.

A large number of complexes with N^N chelating ligands were prepared by different research groups using phenanthroline,^65^ diimine^66^ and pyrimidine.^67^ Sun *et al.*^65^ compared the TADF properties of a novel phenanthroline Cu(i)-based emitter (1.39, Scheme 6) with those of its Cu(i) dimeric cationic analogue to investigate nuclearity effects. Both systems revealed efficient TADF (contributing approximately 80% of the total emission) associated with their similar singlet–triplet energy gaps (0.069 and 0.072 eV, for the monomer and dimer, respectively). By replacing phenanthroline with diimine ligands (1.40–42, Scheme 6) Farias *et al.*^66^ isolated a family of copper(i) complexes characterized by a combination of TADF and phosphorescence with higher energy emissions and lower quantum yields (37–67%) with respect to 1.39. The pyrimidine scaffold, incorporating a pyridine moiety to enhance its coordination capabilities, was used by Skvortsova *et al.*^67^ to obtain a series of mononuclear complexes based on 4-(3,5-dimethyl-1*H*-pyrazol-1-yl)-2-(pyridin-2-yl)pyrimidine as the ligand (1.43–44, Scheme 6). The complexes exhibit a very weak emission (*Φ* <1%) due to the low S_0_–T_1_ energy gap which favors nonradiative deactivation.

**Scheme 7:**
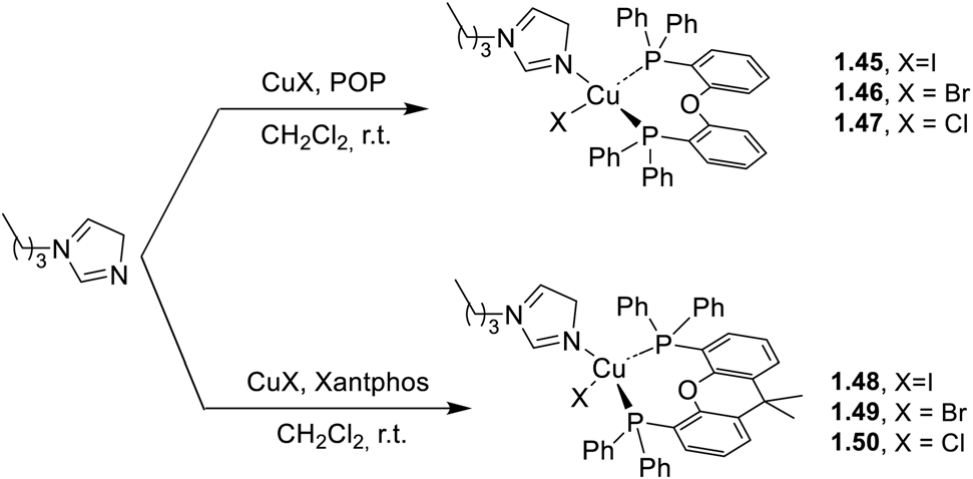
Synthesis of complexes 1.45–47 and 1.48–50.

To obtain large steric hindrance aimed at effectively inhibiting nonradiative transitions, Pan *et al.*^68^ selected bulky and rigid P^P ligands, namely POP (1.45–47) or Xantphos (1.48–50), in combination with 1-butylimidazole as the N-ligand which, by virtue of its electron-rich property, allows obtaining blue-emitting Cu(i) complexes. Based on this strategy, the authors synthesized (Scheme 7) six blue emissive derivatives characterized by intense emission (*Φ* from 39% (1.50) to 92% (1.45)) in the 444–480 nm range.

In addition, the authors performed single crystal X-ray diffraction (SCXRD) studies and photophysical investigations on thermally desolvated and solvated (by DCM and THF vapour exposure) phases. Interestingly, after heating at 333 K, 1.45-CH_2_Cl_2_ and 1.48-CH_2_Cl_2_ display opposite behavior, the former showing hypsochromic emission, while the latter bathochromic one. The heating process does not lead to structural collapse but promotes the formation of a new crystal phase.

**Scheme 8:**
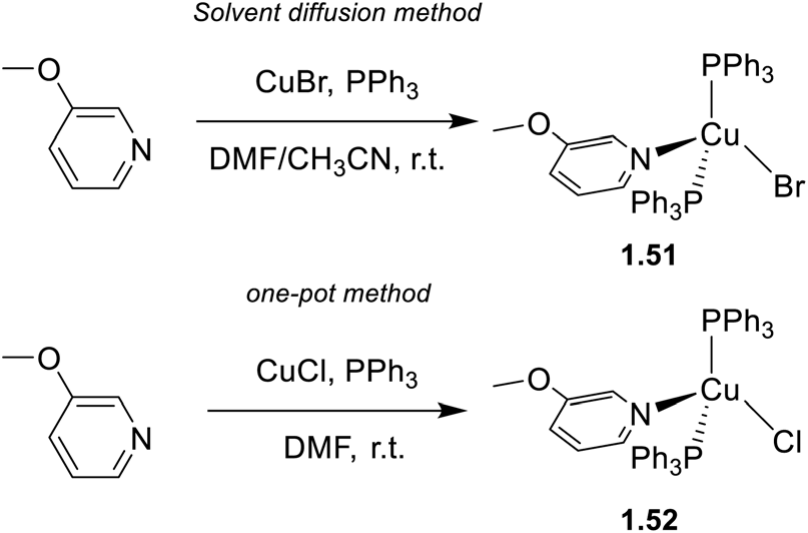
Synthesis of complexes 1.51 and 1.52.

Monodentate 3-methoxypyridine together with triphenylphosphine were used by Zhu *et al.*^69^ to prepare tetracoordinated 1.51 and 1.52. 1.51 was obtained as colorless transparent crystals with green emission under UV light, *via* a solvent diffusion method starting from CuBr and the ligands in ACN/*N*,*N*-dimethylformamide (DMF). In contrast, 1.52 was prepared by a one pot method, as reported in Scheme 8.

Both compounds exhibit remarkable TADF (*Φ* up to 95%), as supported by variable temperature photoluminescent investigations, associated with small Δ*E*_ST_ (below 0.1 eV).

#### 2.2.2 Trigonal compounds

**Scheme 9:**
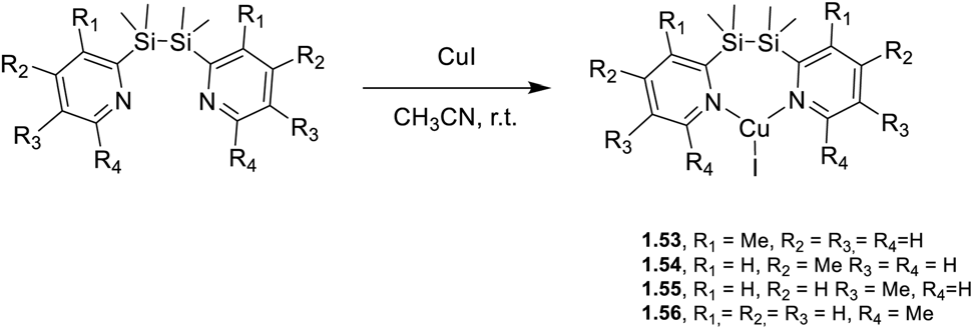
Synthesis of complexes 1.53–56.

Only one report referring to a monomeric neutral Cu(i) compound with trigonal coordination geometry has appeared in the literature during the 2020–mid-2025 period. Zhao *et al.*^70^ synthesized a series of complexes with 1,2-bis(methylpyridin-2-yl)disilane ligands, 1.53–56, Scheme 9. The compounds display AIE features, as revealed by solvent/nonsolvent (THF/water) photophysical investigations. Moreover, in powders, they display efficient TADF (*Φ* from 59 to 86%), consistent with small Δ*E*_ST_ (below 0.1 eV), in the 476–512 nm interval. Notably, the lowest energy maximum is observed for 1.56, characterized by the synperiplanar conformation of the ligand in the crystalline state, different from the others which present an anticlinal one.

## 3. Dimeric complexes

**Scheme 10:**
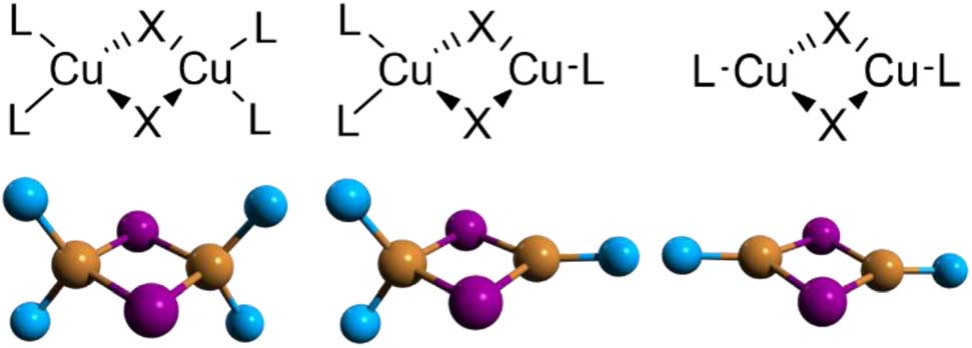
Structure of complexes with {Cu_2_X_2_} cores: left – [Cu_2_X_2_L_4_], centre – [Cu_2_X_2_L_3_] and right – [Cu_2_X_2_L_2_].

Inside the binuclear Cu(i) family, the neutral halogenide-bridged dimeric core, with either a planar or butterfly-shaped {Cu_2_X_2_} motif, is widely encountered in highly luminescent complexes. The greatest number of binuclear complexes are iodo compounds, mainly because of the larger affinity of Cu(i) towards the iodo ligand and the greater stability of copper(i) iodo species toward oxidation with respect to the bromo and chloro analogues. In such rhomboid dimers, the most common structural unit is [(Cu_2_X_2_)L_4_], where four surrounding ligands satisfy the preferential tetrahedral arrangement around copper(i) to result in both homoleptic or heteroleptic derivatives (Scheme 10 left). Halogenido bridged compounds based on [(Cu_2_X_2_)L_3_] or [(Cu_2_X_2_)L_2_] units (Scheme 10, centre and right, respectively), with copper assuming trigonal coordination geometry, are also found, especially when bulky or chelating ligands are involved, but they represent a minority inside the family.

**Scheme 11:**
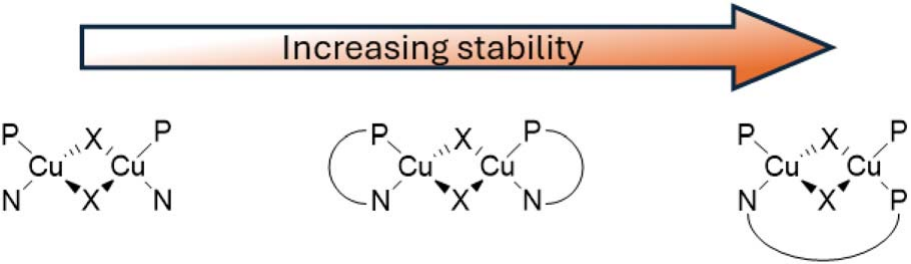
Increasing stabilization through chelating ligands.

Since soft phosphine is one of the favourite ligands of Cu(i) ions, various heteroleptic complexes with two PR_3_ and two N-coordinating ligands have been prepared, with [(Cu_2_X_2_)L_2_(PR_3_)_2_] (X = Cl, Br, and I; L = Py and picoline (Pic); R = phenyl and *n*-butyl) being among the first complexes of this type to be isolated and characterized in 1970 by Jardine.^71^ These compounds possess intrinsic lability which often results in a drastic change of their potentially interesting photoluminescent features upon processing. This limitation can be prevented by using bridging and chelating ligands^72,73^ which also impart rigidity to the system and, therefore, results in brighter emission (Scheme 11). In this regard, bidentate 2-(diphenylphosphino)pyridine and its derivatives have been revealed as interesting candidates^74^ to obtain emissive halogenide bridged compounds of general formula [(Cu_2_X_2_)(L)_2_(P^N)].

**Scheme 12:**
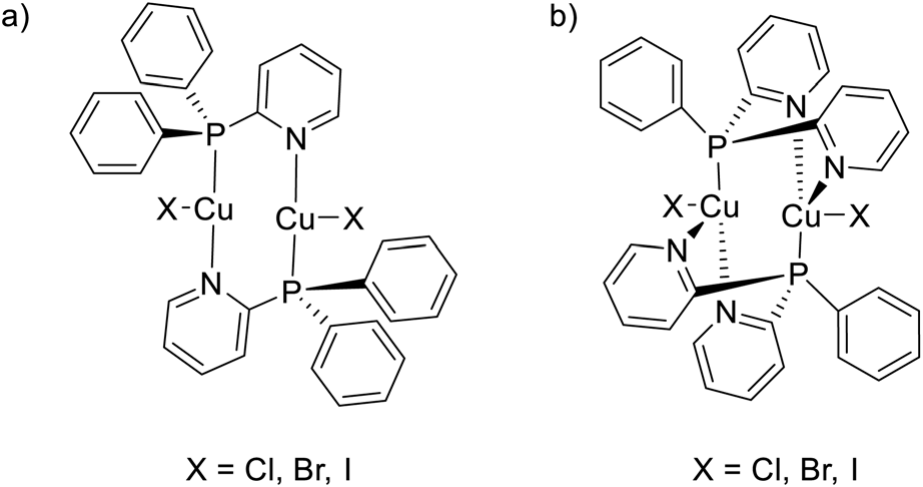
Examples of [Cu_2_(P^N)_2_X_2_] with trigonal (a) or tetragonal (b) geometries.

Importantly, these ligands have also been exploited in the preparation of another, more recent, family of emissive dimeric complexes of general formula [Cu_2_(P^N)_2_X_2_] with halogens as terminal groups, first reported by Yersin and coworkers in 2015 ^75^ (Scheme 12a). This family, having the copper atoms coordinated by nitrogen, phosphorus and halogen atoms, represents a straightforward example of emissive Cu(i) trigonal-planar geometry. With an additional Py on the phosphorus atom, dimeric tetracoordinated complexes with formula [Cu_2_(N^P^N)_2_X_2_] having terminal halogens atoms have also been prepared for the first time in 2016 by Kato (Scheme 12b).^76^

Cu(i) species with different nuclearities can easily interconvert in solution due to the lability of the Cu(i) ion, making it difficult to orient the synthesis towards a desired target. An effective way to synthesize hybrid structures with {Cu_2_I_2_} inorganic modules is to use bulky ligands. Frequently, one pot approaches have been fruitfully exploited for the preparation of homoleptic [(Cu_2_X_2_)L_4_] complexes. For example, many [(Cu_2_I_2_)L_4_] complexes are prepared through mixing a CuI solution, in either ACN or KI saturated aqueous solution, with the corresponding organic ligand. Another approach is based on ligand exchange starting from a dimeric complex stable in solution where an excess of substituting ligand is dissolved.

In recent years, the number of reports on photoluminescent dinuclear Cu(i) complexes has been rapidly increasing due to their interesting features. Frequently, in fact, the emissive excited states of {Cu_2_X_2_} complexes are to be associated with CT transition from the {Cu_2_X_2_} core to the available π* orbitals of the ligands. On excitation, the oxidation process involves the entire inorganic core and results in a reduced excited state distortion and, consequently, in bright luminescence. Moreover, the high charge-transfer nature of such (M + X)LCT states creates a spatially well-separated electron and hole, opening to the possibility of TADF. Among others, many Cu_2_I_2_ derivatives have been found to display efficient TADF and/or phosphorescence associated with SOC of the Cu_2_I_2_ center (*ξ*(Cu) = 857 cm^−1^ and *ξ*(I) = 5069 cm^−1^), where Cu and I synergistically promote the ISC/RISC rate. Through proper selection of the π* level of the ligands, emissions in the whole visible region can be observed.

It has to be considered that, sometimes, in dimeric complexes with the Cu⋯Cu distance shorter than the sum of Cu(i) vdW radii, ^3^CC states can take part in the emission process with effects related to the specific Cu⋯Cu distance and coordination geometry (tetrahedral or trigonal).

Importantly, the possibility to combine phosphorescence and TADF at r.t., which was first disclosed for the family of dimeric Cu(i) compounds of formula [Cu_2_(P^N)_2_X_2_] (where the (N^P) bridging ligand is 2-(diphenylphosphino)-6-methylpyridine and X = Cl, Br, or I) by Yersin in 2015,^75^ has significant advantages in OLED technology. Dimeric Cu(i) dual emitters have already been successfully applied as concomitant singlet and triplet harvesting materials.^77,78^

### 3.1 Advancements during 2020–mid-2025

The present section has been divided into two main subsections, one related to the majority of halogen bridged rhomboid dimers and the other to complexes with halogens as terminal ligands. A summary of the photophysical properties of the mentioned compounds is reported in Table S3.

#### 3.1.1 Halogen bridged rhomboid dimers

##### 3.1.1.1 Homo- and heteroleptic complexes containing monodentate N-donor ligands

**Scheme 13:**
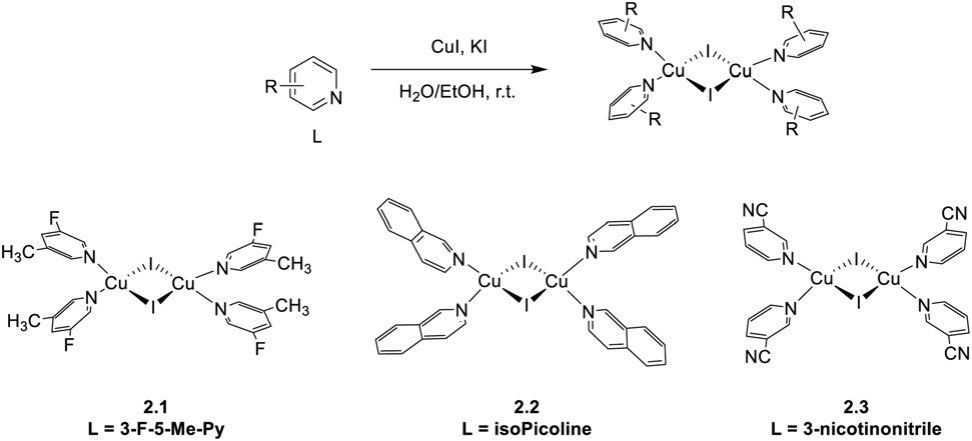
Synthesis of compounds 2.1–3.

New binuclear rhomboid homoleptic compounds of formula [(Cu_2_I_2_)L_4_] (where L are nitrogen donor monodentate bulky ligands) were reported in 2021 and 2024.^78,79^ In the 2021 paper,^79^2.1, 2.2, and 2.3 were prepared by adding to CuI in aqueous KI a solution of 3-fluoro-5-methyl-pyridine, isoquinoline, or nicotinonitrile, respectively (Scheme 13). The three compounds are soluble and non-emissive in common organic solvents but emit as solids at r.t. from green to red phosphorescence with *Φ* equal to 43.3, 13.5, and 5.1% and lifetimes of 12.5, 6.2 and 1.4 μs for 2.1, 2.2 and 2.3, respectively.

**Scheme 14:**
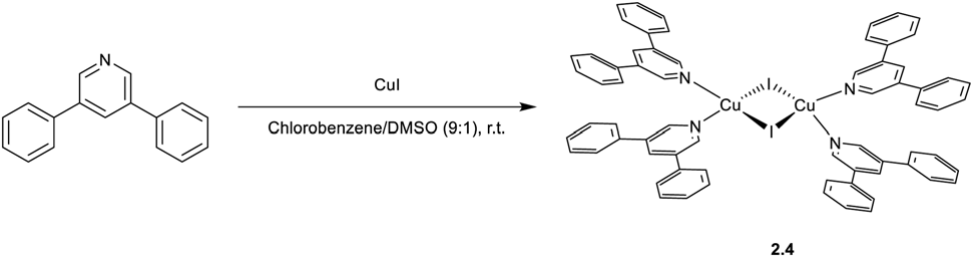
Synthesis of 2.4.

In 2024,^78^2.4 was prepared by a one pot reaction in solution using 3,5-diphenylpyridine as the ligand (Scheme 14). The film of 2.4 displays at r.t. green emission at 515 nm which, through variable temperature experiments, was assigned to concomitant TADF (facilitated by a Δ*E*_ST_ equal to 0.082 eV) and phosphorescence (with contributions equal to 88.6% and 11.4%, respectively). The compound was used to prepare solution-processed non-doped OLEDs with a maximum EQE of 4.8% and a brightness of 3895 cd m^−2^.

**Scheme 15:**
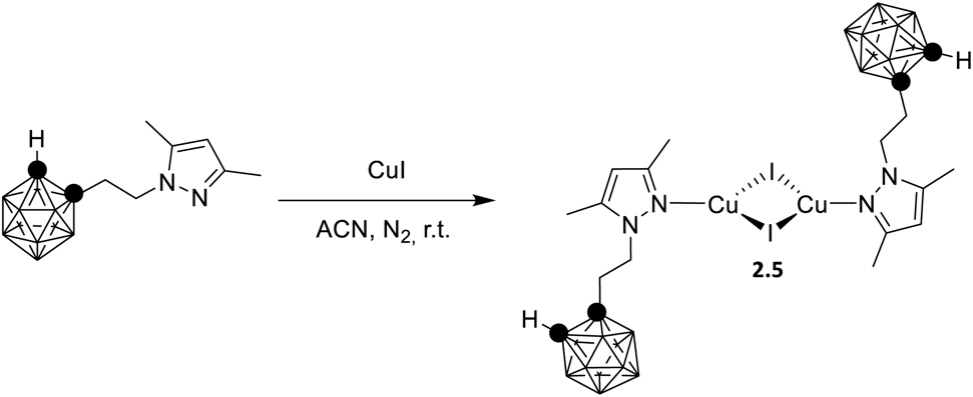
Synthesis of 2.5.

In 2021, by a one pot reaction of CuI with a carborane-based pyrazole ligand, Soldevila-Sanmartin *et al.*^172^ isolated, together with compounds of higher nuclearity (see Section 5), a tri-coordinated rhomboid dimer of formula [(Cu_2_I_2_)L_2_], 2.5 (Scheme 15).^172^ In 2.5, each trigonal-planar Cu(i) atom is bonded to the carboranyl pyrazole ligand through the pyrazole N atom, and Cu⋯Cu distances are much shorter than the sum of Cu(i) vdW radii (2.47–2.51 Å).^172^

Crystalline powders of the compound display bright blue emission (483 nm; *Φ* = 66.5%) which, different from the typical (M + X)LCT origin of tetracoordinated derivatives, was attributed to a cluster centered excited state associated with a d–s transition, as supported by TDDFT calculations.

A larger number of publications have been devoted to heteroleptic complexes containing N- and P-donor ligands.

**Scheme 16:**
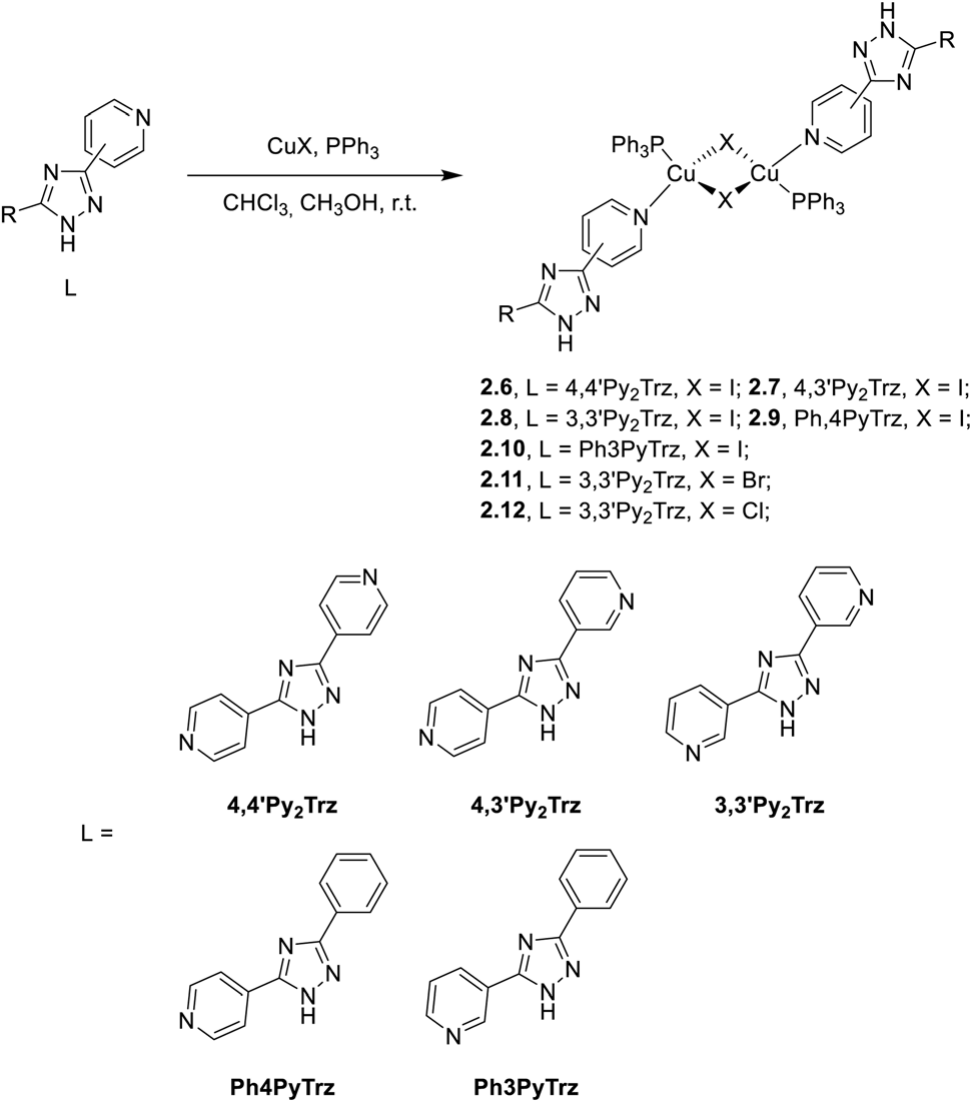
Synthesis of compounds 2.6–12.

**Scheme 17:**
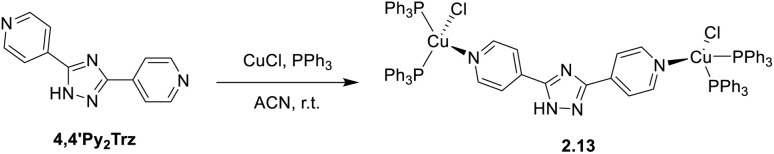
Synthesis of 2.13.

In 2023 and 2024, Gusev *et al.*^80,81^ reported a family of dimeric complexes of general formula [(Cu_2_X_2_)L_2_(PPh_3_)_2_] (where X = Cl, Br and I and L is a 3- or 4-pyridyl triazole derivative). In particular, the authors isolated and characterized five rhombohedral iodide, 2.6–10, one bromide, 2.11, and one chloride, 2.12, complexes in which the triazoles behave as monodentate through the pyridinic nitrogen atom (Scheme 16). In addition, 2.13, having 4-pyridyl triazole as the bridging ligand, was also isolated and characterized (Scheme 17).

All compounds prepared by a one pot reaction of CuX and the corresponding triazole in solution were structurally characterized by SCXRD studies and photophysically investigated in the crystalline state both at room and low temperatures. 2.6–13 display a bright broad emission from either MLCT or (M + X)LCT excited states, as supported by DFT/TDDFT calculations. From variable temperature experiments, r.t. emission of 2.8 and 2.11–13 was associated with efficient TADF (with about 20% phosphorescent contribution for 2.11 and 2.13). Moreover, moderately greenish emissive crystals of 2.9 were transformed into intense yellow powder through grinding (see Section 6.2).

**Scheme 18:**
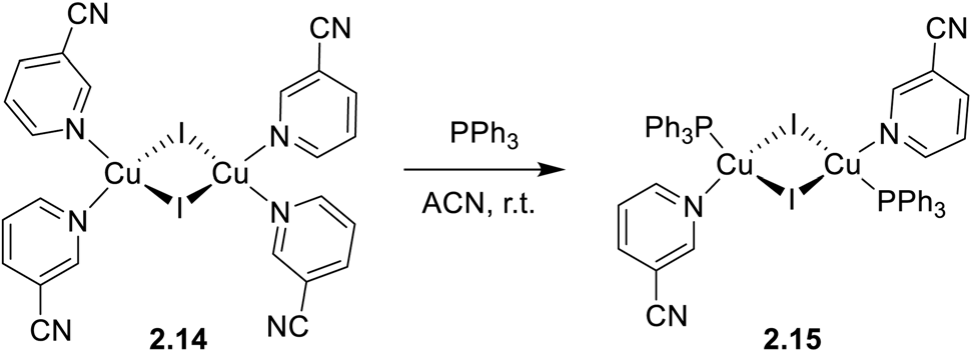
Synthesis of a heteroleptic complex 2.15 from a homoleptic one 2.14.

To assess the role of the ligands in switching from TADF to phosphorescence through variation in the Cu⋯Cu distance in dimeric complexes, Chatterjee *et al.* compared the photophysical behaviour of homoleptic 2.14 and heteroleptic 2.15, both in solution and in the solid state (Scheme 18).^82^

Powders of 2.14 and 2.15, having Cu⋯Cu distances of 2.64 and 3.07 Å, respectively, display green emission (535 nm, *Φ* = 66% and 538 nm, *Φ* = 83%, respectively) originating from two different mechanisms disclosed by variable temperature experiments and supported by theoretical calculations. In particular, 2.14 emits at r.t. through spectrally unresolvable phosphorescences from ^3^(M + X)LCT and ^3^CC states. Upon decreasing the temperature, a gradual increase in the emission intensity is observed accompanied by blue shifting and prolonging of the excited state lifetime (going from ∼6.43 to ∼37.56 μs upon cooling the sample from 300 to 8 K). In contrast, for 2.15, in agreement with its Cu⋯Cu distance longer than the sum of Cu(i) vdW radii, only (M + X)LCT is involved in the radiative deactivation. In particular, 2.15 irradiates through a TADF mechanism from ^1/3^(M + X)LCT with an estimated Δ*E*_ST_ of 0.076 eV, as evidenced by a temperature-dependent emissive study. By lowering the temperature, a sequential red-shift and intensity increase of the emission, along with a remarkable increase in the lifetimes (6.98 μs at r.t. and ∼94 μs at 50 K), was observed. It was therefore proven that, through electronic and steric effects of coordinating ligands, the increase of the Cu⋯Cu distance can effectively activate TADF with a high quantum yield in (M + X)LCT dimeric complexes.

**Scheme 19:**
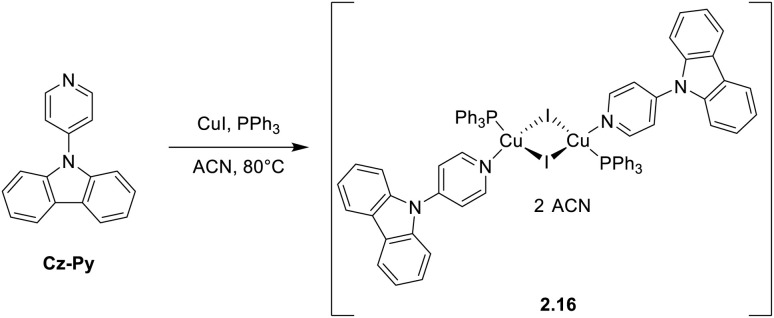
Synthesis of 2.16.

Zhao *et al.* reported the characterization and application as X-ray scintillators of compound 2.16 prepared by the reaction of 9-(pyridin-4-yl)-9*H*-carbazole, PPh_3_ and CuI under solvothermal conditions (Scheme 19).^19^

Through photophysical investigation of a solvent/non-solvent (DMSO/water) mixture, 2.16 was revealed as an AIEgen (best performing with 90% water volume: *Φ* = 21%; 9.33 *τ* = 10.1 μs) due to a restriction of intramolecular rotation (RIR) mechanism. In the crystalline state, at r.t., the compound displays a blue emission (462 nm; *τ* = 9.33 μs) and a yellow afterglow phosphorescence with vibronic replicas at 540 and 580 nm (*τ* = 133.5 ms). Through variable temperature experiments and DFT/TDDFT calculations, the blue emission was assigned to TADF (facilitated by the small calculated Δ*E*_ST_ = 0.075 eV). The long-lived emission was associated with the cocrystallized ACN molecules which restrict molecular vibrations resulting in more degenerate triplet levels. Moreover, thanks to its intense X-ray absorption and concomitant TADF, long-lived phosphorescence and AIE features, 2.16 was successfully applied in the construction of a flexible scintillator screen for high-resolution X-ray imaging with an ultrahigh spatial resolution (23.6 LP mm^−1^).

##### 3.1.1.2 Complexes with bridging P^N ligands

**Scheme 20:**
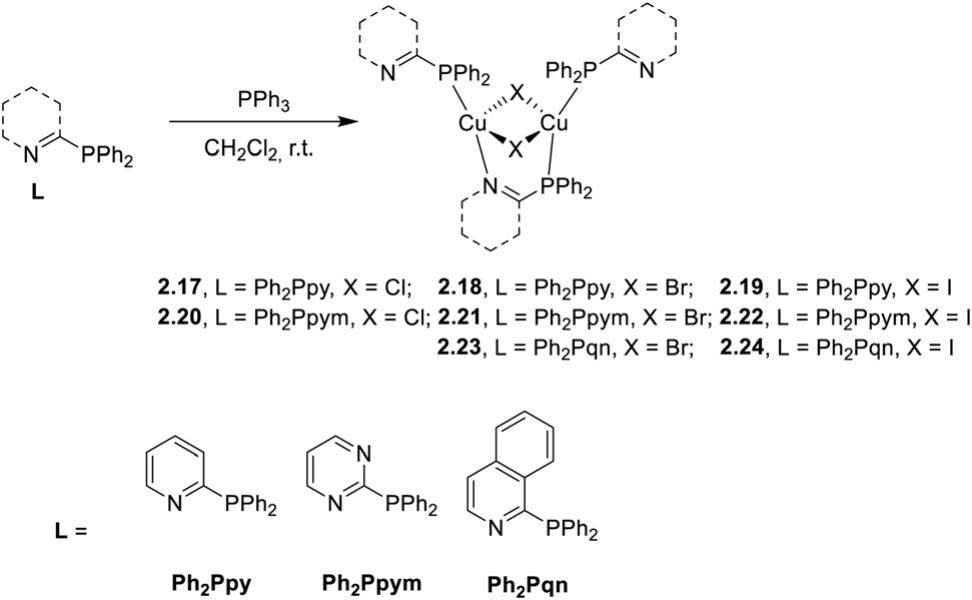
Synthesis of 2.17–24.

Starting from the 2015 seminal work by Yersin and co-workers, bidentate 2-(diphenylphosphino)pyridine has been widely investigated as a P^N-bridging ligand able to stabilize dimeric Cu(i) structures. In 2021, Yersin and co-workers reported an overview of eight (five already known and three new) dimeric, highly emissive complexes of type [(Cu_2_X_2_)L_3_] (X = Cl, Br, and I), where L are bidentate P^N ligands (namely, 2-diphenylphosphino-pyridine, Ph_2_Ppy; 2-diphenylphosphinopyrimidine, Ph_2_Ppym; 1-diphenylphosphino-isoquinoline, Ph_2_Piqn) 2.17–24, prepared by a one pot reaction of copper(i) halides with the respective ligands in DCM (Scheme 20).^83^

Powders of all compounds display at r.t. broad emission bands in the green to red spectral range, assigned to (M + X)LCT transitions through DFT/TDDFT calculations performed on [Cu_2_I_2_(Ph_2_Ppy)_3_], 2.19, chosen as a prototype. In agreement with their origins, the emissions are red shifted in the presence of more extended aromatic ligands. Moreover, in each series, the emission maxima are blue shifted on going from Cl to I. From variable temperature experiments and the supporting detailed theoretical calculations on 2.19, the r.t. emission was found to be associated with concomitant TADF and phosphorescence. In accordance with the proposed dual mode emission, at 77 K, a large increase in lifetimes is accompanied by a small red shift in PL spectra, revealing S_1_–T_1_ energy gaps (in the 37–88 meV interval) compatible with thermal activation of the DF. The contribution of phosphorescence (20% at r.t.) was associated with fast T_1_–S_0_ radiative rates, the fastest one being that of 2.19 prototype (*k*_r_ = 2.88 × 10^4^ s^−1^ at 77 K), thanks to the high SOC constant of iodide and the large contributions of its 5p-orbitals to the HOMO.

**Scheme 21:**
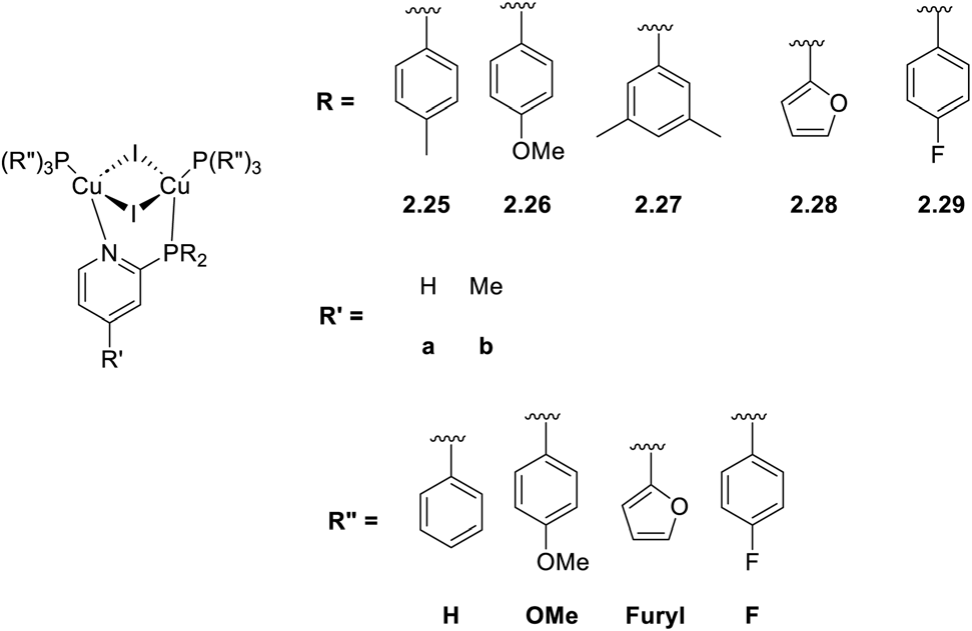
Synthesis of 2.25–29.

In 2021, Busch *et al.* reported a family of phosphino-modified 2-(diphenylphosphino)pyridine ligands used for the one pot preparation of dimeric [(Cu_2_I_2_)(P^N)(PR_3_)_2_] complexes in dichloromethane (CH_2_Cl_2_, DCM) solution (2.25–29, Scheme 21).^74^

The compounds were fully photophysically characterized as powders and blended films. Powders of 2.25–29 display emission, in the 520–560 nm range, from (M + X)LCT excited states very poorly affected by substituents on the phosphine moieties, with lifetimes increasing 6–7 times on going from 298 to 77 K (values in the range of 4.4–8.1 μs and 6.5–44.2 μs, respectively). This behaviour can be considered compatible with TADF emission. Doped films in the PMMA matrix were studied both at 298 and 77 K where hypsochromic shift was observed. Such blue shifting was associated with rigidochromic effects prevailing over electronic ones induced by TADF. Moreover, performances of the compounds in solution-processed OLED devices with several different heterostructures were investigated, with the best performances obtained for 2.25bH reaching a brightness of 5900 cd m^−2^ and a current efficiency of 3.79 cd A^−1^.

**Scheme 22:**
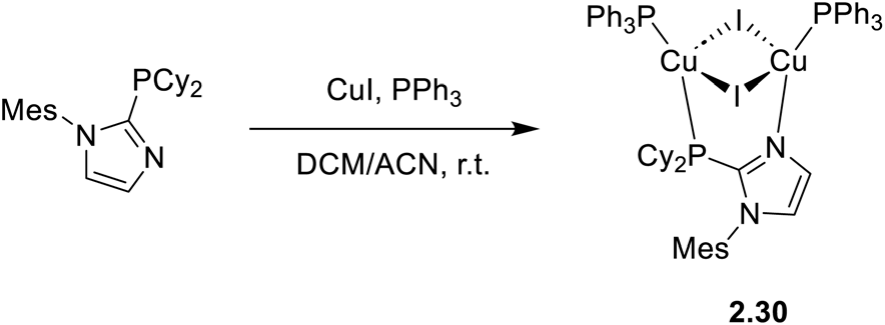
Synthesis of 2.30.

In 2025, Jiang *et al.* reported on the one pot synthesis and extended characterization of a Cu_2_I_2_ dimer, namely 2.30 [(Cu_2_I_2_)(P^N)(PPh_3_)_2_], wherein 1-(2,4,6-trimethylphenyl)-2-(dicyclohexylphosphino)imidazole acts as the P^N bridging ligand (Scheme 22).^84^

2.30 crystals display at r.t. bright blue emission centered at 445 nm (*Φ* = 74.2%; *τ* = 15.6 μs) which, in agreement with the small calculated Δ*E*_ST_ value (0.083 eV) and variable temperature experiments, was mainly (82% contribution) assigned to TADF (from excited states of (M + X)LCT character). In particular, a gradual red shift from 445 to 461 nm and a concomitant decrease in the emission intensity were observed on moving from 298 to 77 K. Moreover, a remarkable lengthening of the emission lifetime was observed at low temperature (114.3 μs at 100 K). 2.30 was also identified as an AIEgen being hardly emissive in THF solution but with emission intensity increasing in solvent/non-solvent (THF/water) mixtures with increasing non-solvent percentage (the highest intensity observed with 90% water content). This AIE behavior was attributed to the restricted intramolecular motion (RIM) mechanism associated with abundant intermolecular interactions in the aggregate phase. In addition, since the compound displayed thermal, chemical and photo-stability, together with solution processability, it was tested to assess its scintillation performance. 2.30 exhibited strong radioluminescence and excellent radiation stability, along with an ultra-low detection limit ascribable to the effective X-ray absorption by the heavy Cu_2_I_2_ core and the high radiation-induced exciton utilization efficiency in the TADF process.

##### 3.1.1.3 Complexes with chelating P^N ligands

**Scheme 23:**
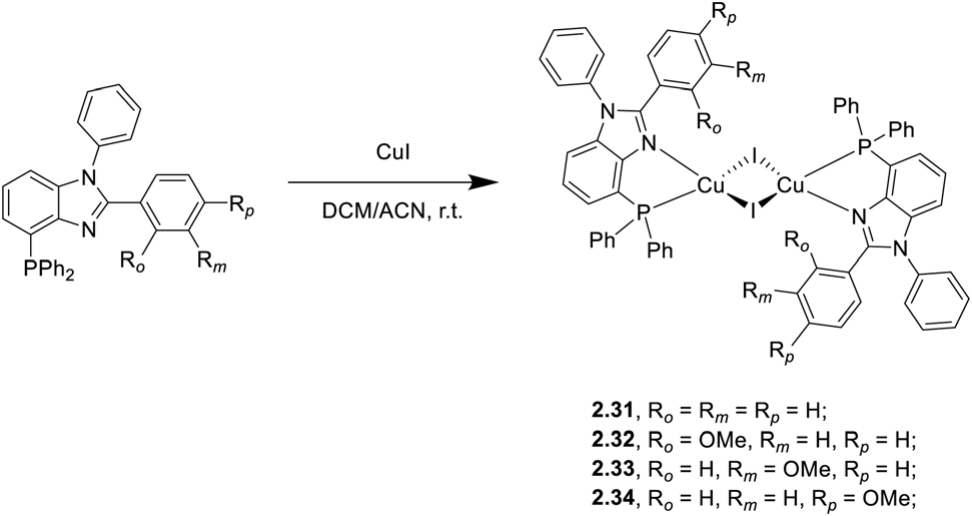
Synthesis of 2.31–34.

In 2021 and 2025, four iodide-bridged dinuclear complexes, 2.31–34, of general formula [(Cu_2_I_2_)(P^N)_2_] (where P^N is a bulky chelating ligand based on the 4-diphenylphosphino-benzimidazole scaffold) were prepared by a one pot reaction between CuI and the P^N ligand in DCM/ACN solution (Scheme 23).^85,86^

The four compounds are non-emissive in solution, due to geometry distortions in excited states, but quite so as powders (*Φ* = 34–49%) with the highest value for 2.34. The broad, unstructured bands in the greenish-yellow region (553–586 nm) of the four solid compounds were assigned to excited states of (M + X)LCT character. From steady state and lifetime measurements at different temperatures, deactivation of complex 2.31 at r.t. was associated with TADF (facilitated by the small Δ*E*_ST_, 0.020 eV), while those of 2.32, 2.33 and 2.34 with phosphorescence. From comparison of 2.34 with its monomeric analogue having a much lower *Φ* (6%, 559 nm, and *τ* = 0.765 μs), the positive double role of the Cu_2_I_2_ core in giving structural rigidity and promoting ISC was highlighted. 2.31–33 were investigated as dopants in solution processed OLED devices, with 2.31 resulting as the best performing dye with a peak brightness of 3325 cd m^−2^ and an EQE of 2.99%.

**Scheme 24:**
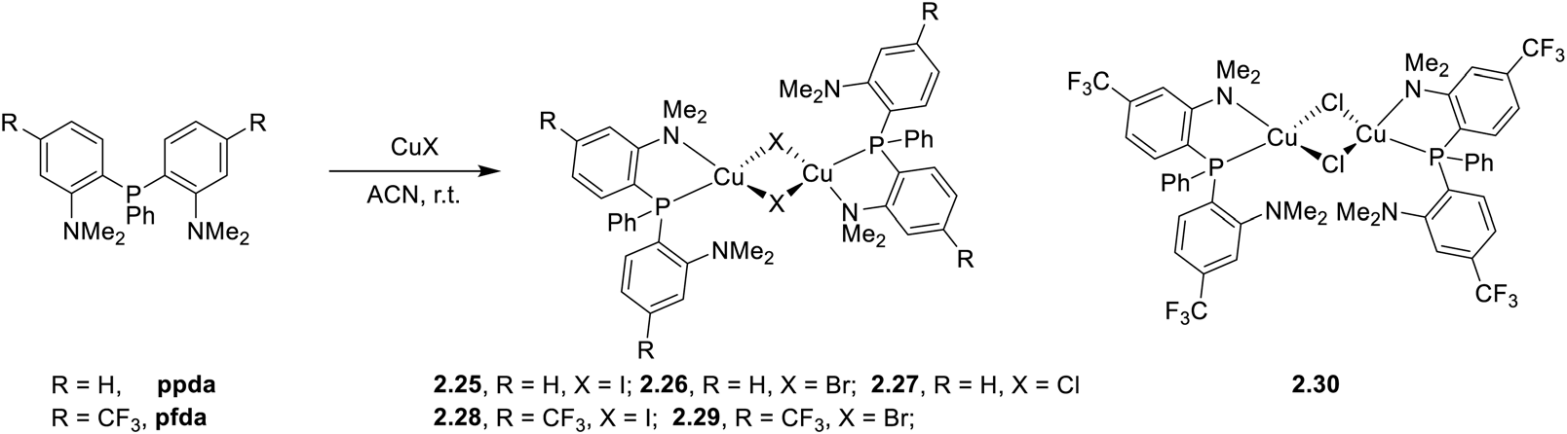
Synthesis of 2.35–40.

Similarly, in 2021, six dimeric Cu(i) complexes of general formula [(Cu_2_X_2_)(P^N)_2_] were prepared by a one pot reaction in solution using either 2-[2-(dimethylamino)phenyl(phenyl)-phosphino]-*N*,*N*-dimethylaniline, ppda, or 2-[2-(dimethylamino)-4-(trifluoromethyl)phenyl-(phenyl)phosphino]-*N*,*N*-dimethyl-5-trifluoromethylaniline, pfda (Scheme 24).^87^

SCXRD analysis revealed a planar Cu_2_X_2_ unit in complexes 2.35–39 and a butterfly-shaped one in 2.40. Powders of all compounds display luminescence from (M + X)LCT states. At r.t., 2.35, 2.38, and 2.39 show intense, long lived (lifetimes in the 0.4–19.2 μs interval) emissions in the 443–570 nm range. These emissions were attributed to TADF on the basis of the observed relevant increase of the lifetimes together with their red shift at 77 K, the small estimated Δ*E*_ST_ values (0.095–0.1667 eV) and variable temperature photoluminescence studies performed on 2.38. On the other hand, 2.36, 2.37 and 2.40 exhibit very weak, fast (lifetimes in the nanosecond regime, 4.4–9.3 ns) emission in the 534–595 nm region. The estimated large Δ*E*_ST_ values (0.3373–0.4184 eV) of these compounds were considered responsible for the observed prompt fluorescence. 2.38 was also investigated as an emitting material in solution-processed OLED devices, giving a maximum EQE of up to 0.17% and a luminance of 75.52 cd m^−2^.

**Fig. 6:**
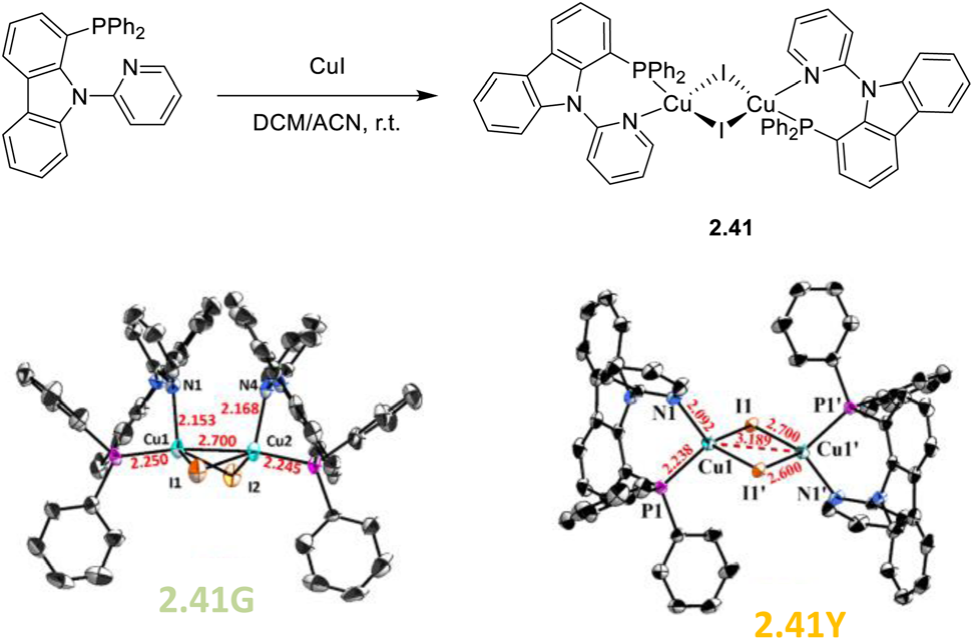
Top: synthesis of 2.41. Bottom: crystal structures of 2.41G (left) and 2.41Y (right). Adapted with permission from ref. 88. Copyright 2023, The Royal Society of Chemistry.

In 2023, Zhang *et al.* reported on the binuclear Cu(i) complex [(Cu_2_I_2_)(P^N)_2_], 2.41 (P^N = 1-(diphenylphosphino)-9-(pyridin-2-yl)-9*H*-carbazole, DPPCz). By a one pot reaction of CuI in ACN and DPPCz in DCM (Fig. 6 top), crystals of a green emissive (518 nm; *Φ* = 43%) polymorph, 2.41G (Fig. 6 bottom left), were obtained.^88^

On standing in solution at r.t. for 48 h, these crystals transform through spontaneous ligand rotation into a yellow emissive (550 nm; *Φ* = 18%) polymorph, 2.41Y (Fig. 6, bottom right). The crystal structure of the two polymorphs was determined by SCXRD analysis, and their different photoluminescent behaviour was interpreted with the aid of DFT/TDDFT calculations. The 9-(pyridin-2-yl)-9*H*-carbazole groups on the two DPPCz ligands were located on the same side in 2.41G, resulting in high steric hindrance and largely distorted geometry which justified its tendency to convert into 2.41Y. Variable temperature photophysical characterization, together with theoretical calculations performed on the two isomers, supported their TADF behavior guaranteed by their small calculated Δ*E*_ST_ (0.08 and 0.07 eV for 2.41G and 2.41Y, respectively) between S_1_ and T_1_ states of mixed MLCT, XLCT and ILCT character, with greater MLCT contribution in 2.41G.

##### 3.1.1.4 Complexes with chelating N^N ligands

**Fig. 7:**
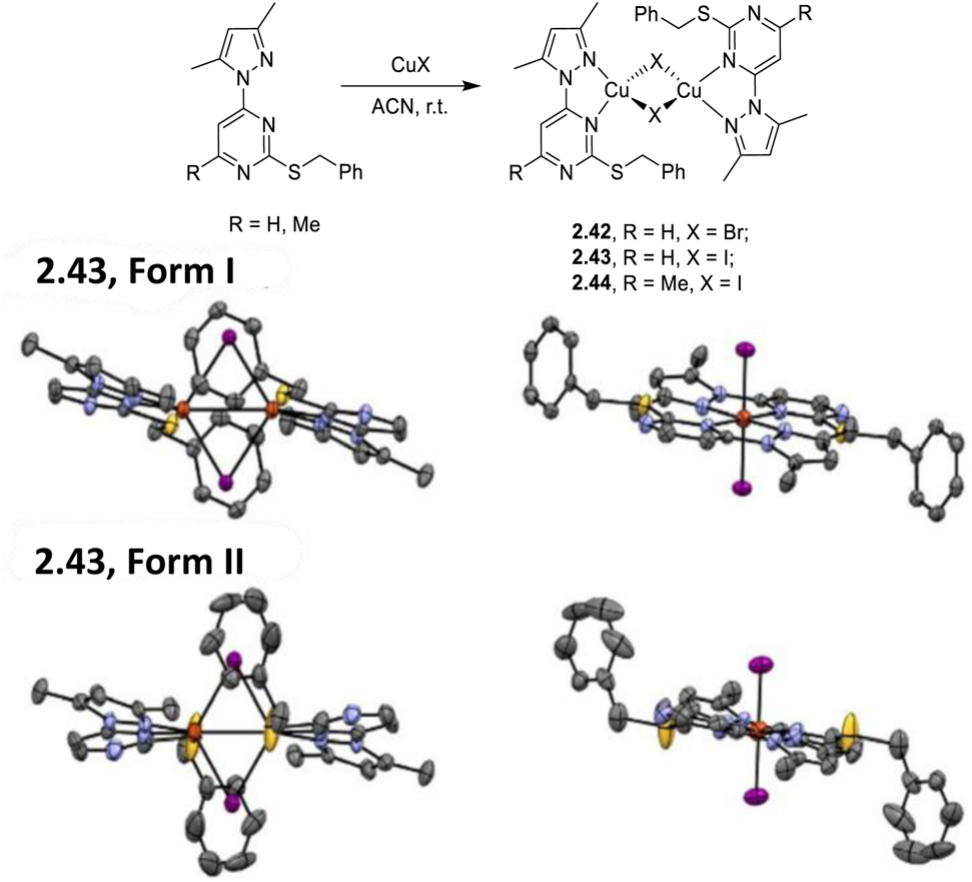
Top: synthesis of 2.42–44. Crystal structures of 2.43 – Form I (middle) and Form II (bottom). Adapted with permission from ref. 89. Copyright 2025, The Royal Society of Chemistry.

In 2025, Skvortsova *et al.* reported a family of dimeric complexes of general formula [(Cu_2_X_2_)(N^N)_2_] (N^N = LH, 2-benzylthio-4-(3,5-dimethyl-1*H*-pyrazol-1-yl)pyrimidine, where X = Br (2.42) and I (2.43); N^N = LMe, 2-benzylthio-4-(3,5-dimethyl-1*H*-pyrazol-1-yl)-6-methylpyrimidine, where X = I (2.44)) prepared by a one pot reaction in ACN of CuX with either (L^H^) or (L^Me^) (Fig. 7 top).^89^2.43 was isolated as two polymorphs: Form I, which is isostructural with 2.42 and 2.44, and Form II. Remarkably, while these compounds display in the solid state quite similar features (position, shape and nature) of the emission band, assigned to an ^3^XLCT excited state according to DFT/TDDFT calculations, their *Φ* values are very different (3 and 8% for Forms I and II, respectively). This unexpected result was associated with 0.2 Å difference in the Cu⋯Cu distances (2.86 Å *vs.* 2.65 Å for Forms I and II, Fig. 7 middle and bottom, respectively) and the related structural modifications. Through calculations, it was concluded that the longer Cu⋯Cu distance of Form I, as well as that of 2.42, is associated with an easier rearrangement in the T_1_ state with a consequent decrease in the T_1_–S_0_ energy gap and an increase in the non-radiative deactivation. The high *Φ* value of 2.44, showing a *d*_Cu⋯Cu_ intermediate between Form I and Form II, is explained by its higher T_1_–S_0_ energy gap, which makes it less prone to non-radiative deactivation.

#### 3.1.2 Dimers with terminal halogen atoms

As stated in the introduction of this section, the studies of Yersin (2015)^75^ and Kato (2016)^76^ have been the basis for a new family of dimeric trigonal [Cu_2_(P^N)_2_X_2_] and tetrahedral [Cu_2_(N^P^N)_2_X_2_] complexes with terminal halogen atoms.

**Scheme 25:**
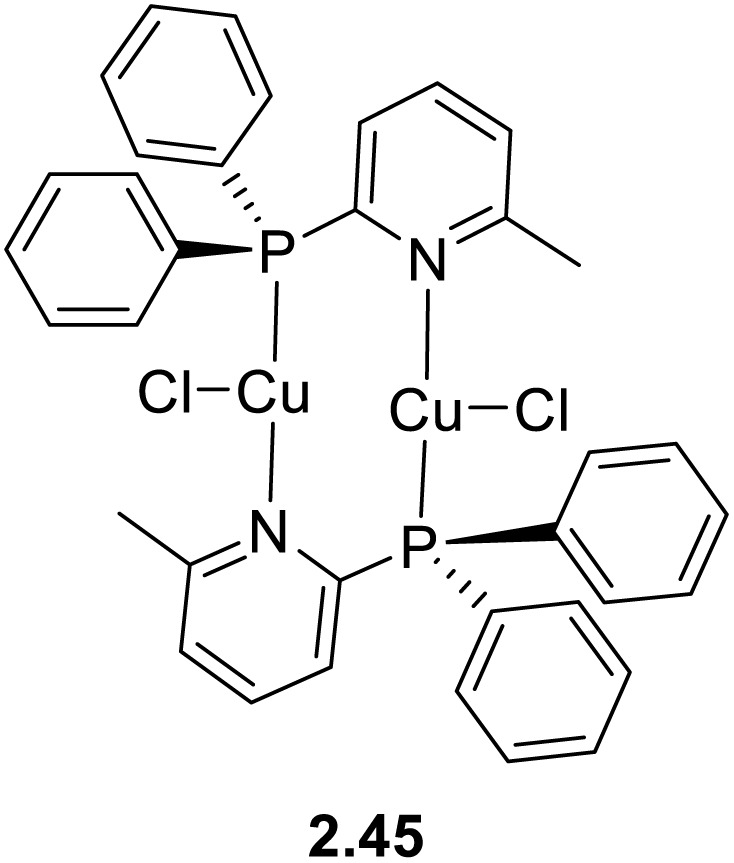
Chemical structure of 2.45.

In 2021, Yersin and co-workers^77^ developed a white emitting solution-processed OLED with an EQE equal to 3.80% by using [Cu_2_(P^N)_2_Cl_2_] (P^N = diphenylphosphanyl-6-methyl-pyridine), 2.45 (Scheme 25), as a single emitter and di(9*H*-carbazol-9-yl)pyridine as the host material for the emissive layer.

This EQE is lower than expected for this compound, which was identified in the 2015 paper as an outstanding candidate for singlet and triplet harvesting due to concomitant TADF and phosphorescence at r.t. (contributing 80% and 20%, respectively), and its high emission quantum yield and moderate emission lifetime (for powders, *Φ* = 92% and *τ* = 8.3 μs).

In 2023,^90^ this compound was further investigated through milli- to micro-second phosphorescence, femto-second fluorescence and theoretical calculations and compared with its chloride-bridged dimeric [Cu_2_Cl_2_(P^P)_2_] (P^P = 1,2-bis-(diphenylphosphino)benzene) analogue. It was concluded that cuprophilic interaction in Cu_2_Cl_2_(N^P)_2_, which is absent in [Cu_2_Cl_2_(P^P)_2_], is responsible for its much more efficient SOC between the T_1_ and neighboring states, resulting in a shorter radiative T_1_–S_0_ decay time (45 μs) and larger zero-field splitting (ZFS, 1.9 meV).

**Scheme 26:**
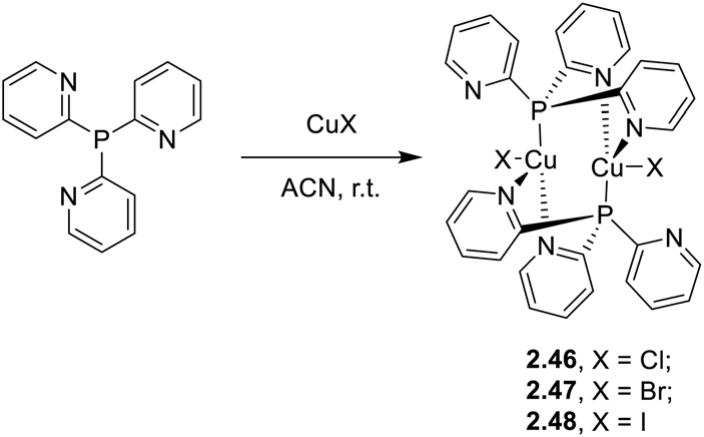
Synthesis of 2.46–48.

In 2020, Baranov *et al.* reported a family of dimeric complexes of general formula [Cu_2_(Py_3_P)_2_X_2_], 2.46–48 (where Py_3_P = tris(2-pyridyl)phosphine; X = Cl, Br, and I), in which two CuX units are N^P^N′-bridged by two ligands (Scheme 26).^91^

The compounds, which can be prepared by either one pot reaction in solution or through liquid assisted grinding of the reactants, display at r.t. bright photoluminescence (520–550 nm, *τ* = 14.5–20.0 μs, and *Φ* ≈ 53%) due to concomitant TADF and phosphorescence from singlet and triplet states of (M + X)LCT character, with relative importance related to the halide. In particular, TADF contribution increases in the order of Cl < Br < I (27, 35 and 61%, respectively). On the basis of experimental and theoretical results, such trend was related to the increasing SOC and decreasing Δ*E*_ST_ (Cl, 0.186 eV > Br, 0.155 eV > I, 0.124 eV) along the halogen group.

**Scheme 27:**
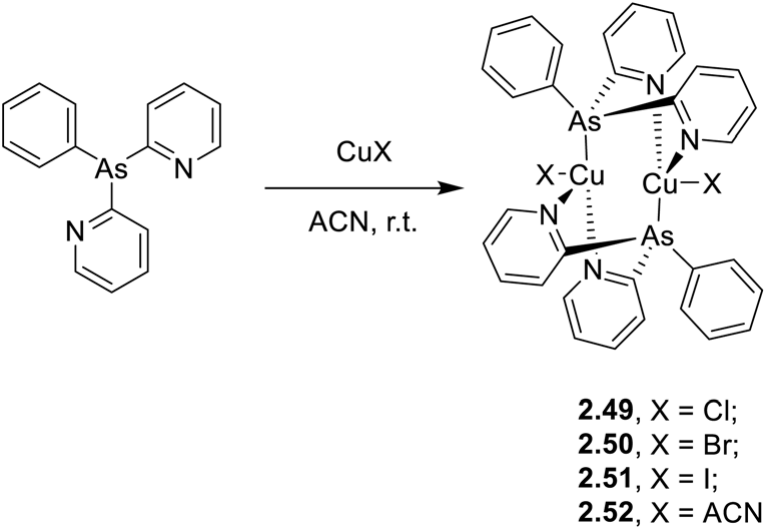
Synthesis of 2.49–52.

In 2022, some of the same authors reported a family of dimers of general formula [Cu_2_(Py_2_AsPh)_2_X_2_] (with Py_2_AsPh = bis(2-pyridyl)phenylarsine and X = Cl, Br, and I), 2.49–52, analogous to those reported by Kato in 2016 ^76^ but having arsine instead of phosphine ligands (Scheme 27).^92^

The compounds, prepared by a one pot reaction in solution, display in the solid state at r.t. a single (M + X)LCT broad emission in the 500–530 nm region. Through detailed photophysical and theoretical studies, the emission was assigned to concomitant TADF and phosphorescence with contribution related to the halide, the TADF proportion increasing in the order of Cl (51%) < Br (75%) < I (78%). The resulting lifetimes are significantly shorter (2–9 μs at 300 K) than those of their phosphine congeners (5–33 μs) due to the higher SOC strength of arsenic (*ξ* = 1202 cm^−1^) with respect to that of phosphorus (*ξ* = 230 cm^−1^).

**Scheme 28:**
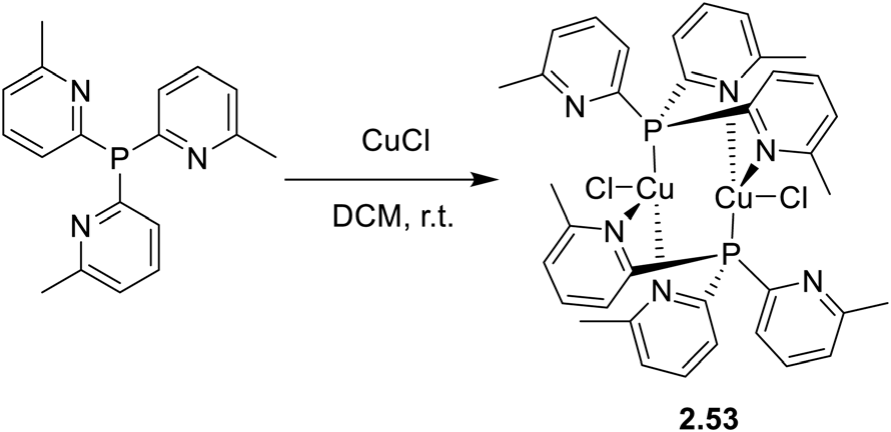
Synthesis of 2.53.

A dimeric analogue of formula [Cu_2_Cl_2_L_2_] (where L = tris(6-methyl-2-pyridyl)phosphine, 2.53) was isolated in 2024 by Artem'ev *et al.* by reaction of the ligand with CuCl in DCM (Scheme 28).^93^ The compound displays in the solid state at r.t. a green, broad, featureless emission which was assigned, with the support of TDDFT calculations, to ^1/3^(M + X)LCT excited states.

**Scheme 29:**
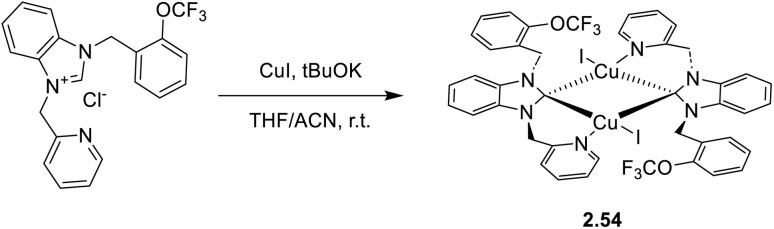
Synthesis of 2.54.

In 2024, a study reported by Yang *et al.* revealed how different N-substituents on the benzimidazole ring of N-heterocyclic carbenes (NHCs) can orient the synthesis towards 0D clusters of Cu_2_ (Scheme 29), Cu_3_ or Cu_4_ nuclearities (the latter reported in Sections 4 and 5, respectively).^94^

The SCXRD analysis of the dinuclear species 2.54 revealed that the carbene carbon possesses an asymmetric bridging mode between the two copper centers. 2.54 is non-emissive in solution, but its powder displays intense phosphorescence at 518 nm (*Φ* = 82% and *τ* = 14.2 μs) from an MLCT excited state, as supported by DFT/TDDFT calculations.

## 4. Trimeric complexes

The interest towards coinage metal-based cyclic trinuclear complexes (CTCs) started in 1970 when Vaughan reported the first Au(i)-CTC opening to a wide range of fundamental and applied research fields.^95,96^ Among d^10^ metal ions, Cu(i)-based CTCs are known for their bright and low-energy r.t. phosphorescence which, together with their cost-effectiveness, make them promising candidates for advanced photo-functional applications.

The majority of Cu(i) trinuclear complexes have the formula [Cu_3_L_3_] with a large planar triangular core in which an angular ditopic anionic ligand (typically pyrazolate, Pz; imidazolate, Im; 1,2,4-triazolate, Trz; or pyridinate, Py) bridges two copper ions. Trimeric compounds based on the [Cu_3_X_3_] unit represent an extremely limited number and will be treated at the end of this section.

### 4.1 [Cu_3_L_3_]-type trimers

The first Cu(i)-CTC was isolated in 1988 by Fackler *et al.*^97^ as a byproduct in Ag(i)-CTC synthesis. Successively, few examples of Cu(i) trimers with different bidentate ligands were reported in the literature for almost 15 years.^98–107^ However, in 2003, Omary *et al.* reported on the photophysical investigation of {Cu[3,5-(CF_3_)_2_Pz]}_3_ opening to a fruitful research field.^108^ Unlike its usual distorted tetrahedral geometry, each Cu(i) in CTCs exhibits a linear and unsaturated two-coordinate conformation resulting in a near-planar [Cu_3_L_3_] ring. This structure favors the formation of dimers/oligomers through intertrimeric cuprophilicity, supramolecular adducts based on π-acid⋯π-base or π-acid⋯Lewis base interactions, and metallocages assisted through host–guest interactions.^109^ The derivatives show interesting luminescence properties related to such supramolecular interactions. However, in the present section, only the presence/absence of cuprophilic interactions and their effect on CTCs photoluminescence are considered.

In the last few decades, many research groups have performed investigations to disclose the effect of Cu⋯Cu interactions on the photo- and chemophysical properties of Cu(i)-CTCs. The results on studies performed until 2020 were collected and analyzed by Li *et al.* in a seminal review on Au(i), Ag(i) and Cu(i) CTCs.^96^ Cuprophilic intra-metallotriangle interactions are not commonly observed due to the high rigidity of Pz, Trz, Im and Py, which impart to the scaffold planar or semi-bent geometries without close metal ion contacts.^110^ Different from intratrimer interactions, intertrimer ones are not affected by the rigidity of the ligands and can be fine-tuned by their fashioning with bulkier or smaller pendants. This higher molecular mobility is also reflected in different intermolecular Cu⋯Cu distances, *d*_Cu⋯Cu_, upon photoexcitation, as first reported by Vorontsov *et al.* for {[3,5-(CF_3_)_2_Py]Cu}_3_.^111^ In many cases, phosphorescence of Cu(i) CTCs is related to intertrimer metal–metal bonding excimers, whose formation is possible even when the ground-state intermolecular *d*_Cu⋯Cu_ is considerably longer than the sum of the vdW radii of the metals. A ground-state 4.0 Å distance has been established as the upper limit to observe an excimeric emissive state (MMCT or ligand-to-metal–metal-bonding charge transfer, LMMCT).^112^ As reported in the following, the ligands used for [Cu_3_L_3_] compounds during the 2020–mid-2025 period are variously substituted pyrazolate. The results are summarized in Table S4.

#### 4.1.1 Advancements during 2020–mid-2025

**Fig. 8:**
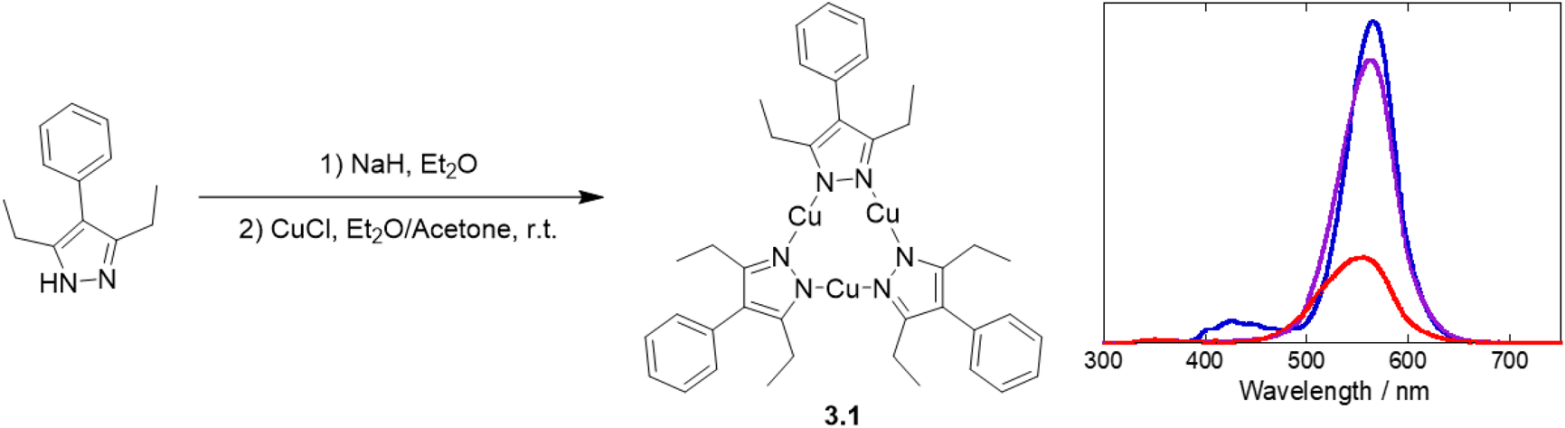
Synthesis of 3.1 (left) and temperature dependent photoluminescence spectra (83 K, blue; 173 K, violet; 298 K, red) at 280 nm excitation wavelength (right). Reproduced with permission from ref. 113. Copyright 2020, Oxford University Press.

In 2020, Fujisawa *et al.* reported an investigation on 3.1, which was prepared by reacting CuCl with 4-phenyl-3,5-diethyl-1-pyrazolate (Fig. 8).^113^ The bulkiness of the ligand resulted in the formation of a crystalline structure composed of isolated trimers with long inter- and intra-trimer Cu⋯Cu distances (in the 4.919–5.322 and 3.206–3.262 Å intervals, respectively).^113^

**Fig. 9:**
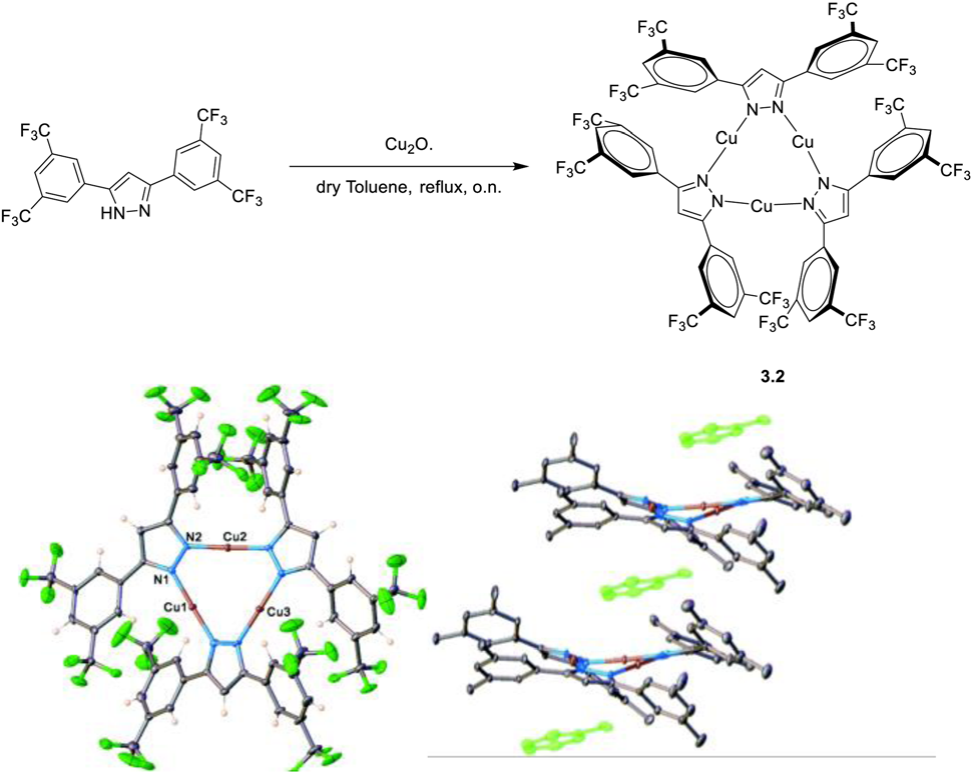
Synthesis (top) and crystal and packing structures (bottom) of 3.2. Reproduced with permission from ref. 114. Copyright 2020, The Royal Society of Chemistry.

Due to its long interchromophoric distances, 3.1 acts as an isolated emitter with solid state photoluminescence dominated by deactivation from a metal centered excited state with a much weaker high energy (HE) contribution of LC origin visible at low temperature. Similarly, as reported by Dias *et al.* in 2020,^114^ crystals of CTC, 3.2, obtained through a one pot reaction in refluxing dry toluene (Fig. 9) present a columnar packing in which the metallotrimer intercalates with toluene molecules which prevent intermolecular cuprophilic interactions. 3.2 is emissive only at 77 K with light blue luminescence at 460 nm. Due to its similarity to the free ligand, the emission was assigned to pyrazole ligand centered luminescence sensitized *via* an internal heavy atom effect.

**Fig. 10:**
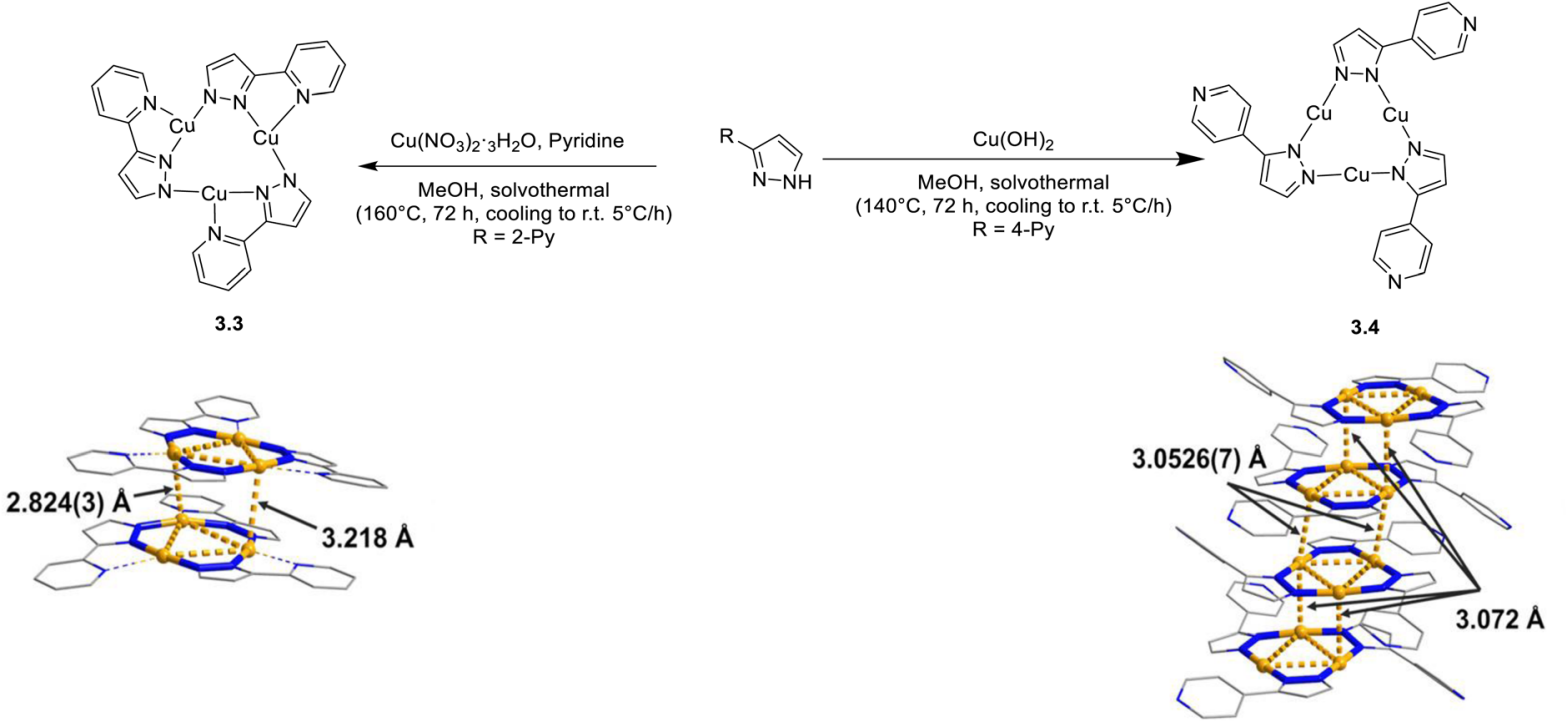
Synthesis and crystal structures of 3.3 (left) and 3.4 (right). Adapted with permission from ref. 115. Copyright 2020, American Chemical Society.

In the same year, Xing *et al.* prepared CTCs 3.3 and 3.4 by reacting, under solvothermal conditions, 3-(4-pyridyl)Pz and 3-(2′-pyridyl)Pz with Cu(NO_3_)_2_·3H_2_O and Cu(OH)_2_, respectively (Fig. 10).^115^ The two compounds display markedly different photoluminescence and structure, with 3.3 having a discrete dimer-of-trimer packing arrangement (shortest intermolecular Cu⋯Cu distances equal to 2.82 Å) and 3.4 organized into a stair-like column.^115^ Under ambient conditions, both compounds show a broad, unstructured emission at about 650 nm, but 3.4 exhibits longer lifetimes and higher *Φ* than 3.3 (27.9 μs, 65% and 5.8 μs, 1.3%, respectively). Moreover, upon cooling to 77 K, a HE (452 nm), ultralong (770.2 μs) and resolved emission appeared in the spectrum of 3.4, while for 3.3 only a bathochromic shift (∼30 nm) and decrease of emission intensity were observed. Through detailed theoretical calculations and experimental results, the emission of 3.3 was assigned to a ^3^MLCT state, while those of 3.4 were attributed to a high-lying ISC route leading to a HE molecular ^3^LC state and a low energy, LE, excimeric one.

**Fig. 11:**
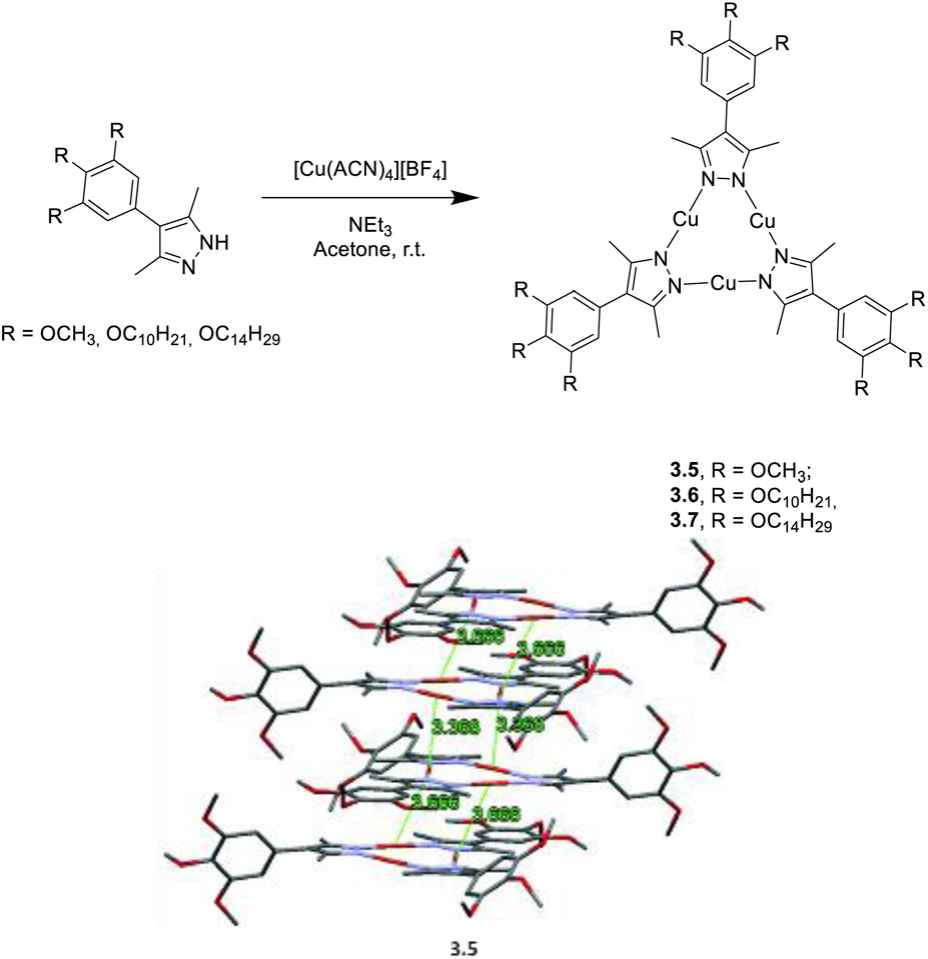
Synthesis of 3.5–7 (top) and crystal packing of 3.5 (bottom). Adapted with permission from ref. 116. Copyright 2020, The Royal Society of Chemistry.

Giménez *et al.* investigated three different Cu(i)-CTCs, 3.5, 3.6 and 3.7, prepared by mixing [Cu(CH_3_CN)_4_]BF_4_ with triethylamine (TEA) and 3,5-dimethyl-4-(3,4,5-(MeO)_3_Ph)Pz or 3,5-dimethyl-4-(3,4,5-(C_10_H_21_O)_3_Ph)Pz or 3,5-dimethyl-4-(3,4,5-(C_14_H_27_O)_3_Ph)Pz, respectively (Fig. 11).^116^ Intriguingly, 3.6 and 3.7 show thermodynamically stable liquid crystal phases.^116^ The three compounds display a similar supramolecular columnar network with molecules cofacially stacked at distances compatible with weak cuprophilic intermolecular interactions (about 3.6 Å). In agreement, neat films of the three CTCs share almost the same photoluminescence comprising one phosphorescence centered at 663, 661 and 664 nm (for 3.5, 3.6 and 3.7, respectively) with similar lifetimes (21, 28 and 26 μs, respectively), suggesting a common origin that authors assigned to the excimer ^3^MM state. Remarkably, the high *Φ* of 3.6 (42%) in the liquid crystalline state represents a record value among the few reported for liquid crystals of phosphorescent complexes.

**Fig. 12:**
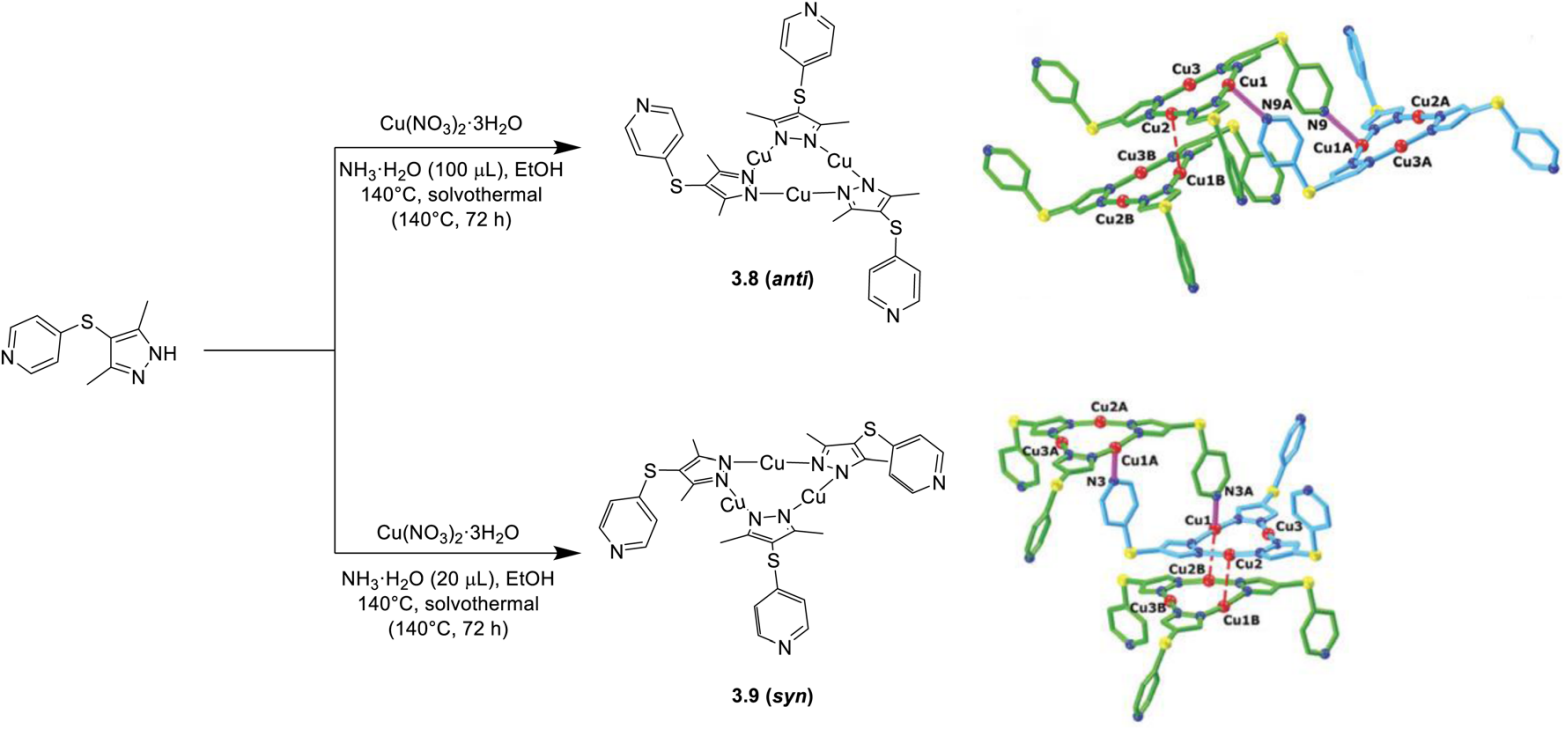
Synthesis of 3.8 and 3.9 (left) and their aggregate fragments (right), supported by intertrimeric N_Py_⋯Cu weak coordination bonds (purple bond) and intertrimeric Cu⋯Cu interactions (red dashed bond). All methyl groups and hydrogen atoms are omitted for clarity. Adapted with permission from ref. 117. Copyright 2021, The Royal Society of Chemistry.

In 2021, Zhan *et al.*^117^ used a 4-(pyridin-4-ylthio)-functionalized 3,5-dimethylpyrazole which can arrange in *syn* or *anti* conformations to isolate two different CTCs, 3.8 and 3.9, through a solvothermal reaction with Cu(NO_3_)_2_·3H_2_O in the presence of different amounts of aqueous ammonia. In crystals of these compounds, one or more Py units of a Cu_3_L_3_ trimer are involved in a weak coordination bond with the linear Cu(i) atom of another trimeric fragment to give dimeric [*anti*-Cu_3_L_3_]_2_ (3.8) and [*syn*-Cu_3_L_3_·C_2_H_5_OH]_2_ (3.9) structures (Fig. 12). Such dimers are further stabilized by intertrimeric Cu⋯Cu interactions (intermolecular *d*_Cu_⋯*d*_Cu_ spanning from 3.08 to 3.57 Å) and, in the case of 3.9, by a cocrystallized solvent molecule. Surprisingly, solid compounds display almost the same photophysical properties with emissions in the 540–583 nm range and lifetimes in the 11–18 μs interval (at 298 K). Through DFT/TDDFT calculations, the emissions were mainly assigned to ^3^MLCT states governed by the weak intertrimeric Cu⋯N_py_ interactions, with additional minor contribution of intertrimeric ^3^MMCT transitions for 3.9, showing the shortest intermolecular Cu⋯Cu contact.

**Fig. 13:**
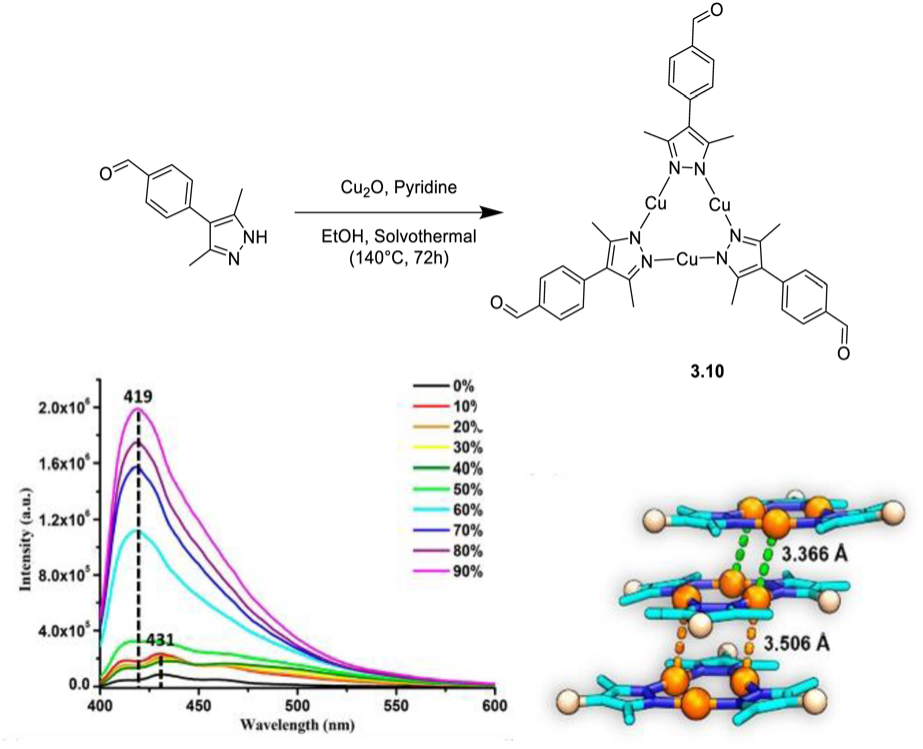
Synthesis (top), water fraction dependent emissive behaviour (bottom left) and crystal packing (bottom right) of 3.10. Adapted with permission from ref. 118. Copyright 2022, American Chemical Society.

Li *et al.* prepared a new CTC, 3.10, by reacting 4-(3,5-dimethyl-*1H*-pyrazol-4-yl)benzaldehyde, Cu_2_O and Py under solvothermal conditions (Fig. 13).^118^3.10 displays AIE behaviour in a solvent/non-solvent (THF/water) mixture with its 467 nm (*τ*_av_ = 0.53 ns) fluorescence *Φ* increasing from <1 to 4% with increment of the water fraction from 0 to 90%. The AIE features have been related to RIR associated with tight molecular packing and strong hydrogen bonding interactions and have been exploited for selective detection and sensing of Au(iii) ions.

**Fig. 14:**
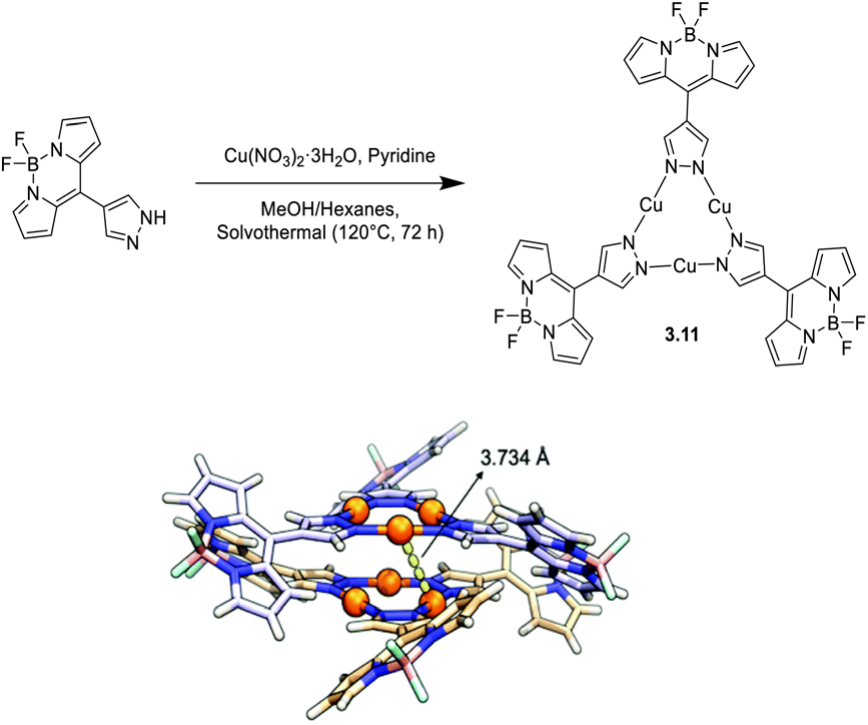
Synthesis (top) and crystal packing (bottom) of 3.11. Adapted with permission from ref. 119. Copyright 2022, The Royal Society of Chemistry.

During the same period, Xia *et al.* reacted a BODIPY-based pyrazolyl ligand with Cu(NO_3_)_2_ under solvothermal conditions to prepare 3.11, a Cu-CTC with enhanced visible absorption (Fig. 14).^119^

3.11 revealed great absorption capability with a large molar extinction coefficient (at 497 nm in DMSO) and ACQ (Aggregation Caused Quenching) behaviour, which made the dye fluorescent only in DMSO (518 nm, 2.75 ns, and *Φ* = 22.2%) or the PMMA matrix. Through DFT/TDDFT calculations and transient absorption experiments in DMSO solution, the authors assigned the strong absorption to a transition from S_0_ to a singlet excited state (S_9_) located on the three ligands, from which the ^1^MLCT S_1_ state is reached through fast internal conversion (IC). From S_1_, molecules can either deactivate through fluorescence or undergo fast ISC to populate ^3^MLCT triplet states (T_4_–T_6_) and then radiatively decay through phosphorescence, after IC to ligand centered LE triplets (T_1_–T_3_). On the basis of its strong visible absorption and long-lived triplet state, 3.11 was successfully investigated for its photooxidation activity.

**Fig. 15:**
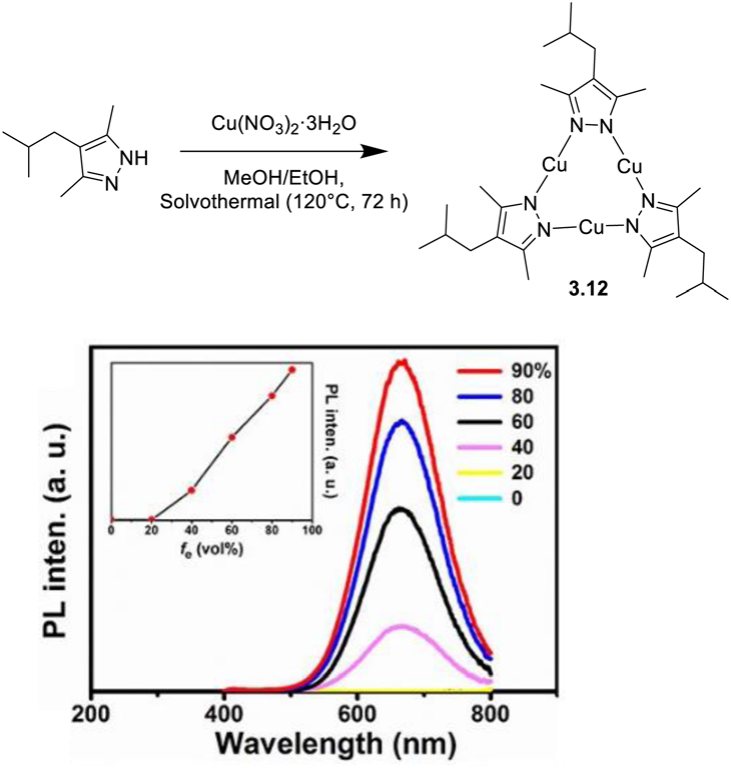
Synthesis (top), crystal packing (bottom left) and water fraction dependent emissive behavior (bottom right) of 3.12. Adapted with permission from ref. 120. Copyright 2022, American Chemical Society.

An opposite AIE behavior was observed in 2022 by Yang *et al.*, for an ultra-bright solid Cu(i)-CTC (*Φ* >99%), 3.12, obtained by reacting 3,5-dimethyl-4-isobutylpyrazole with Cu(NO_3_)_2_ under solvothermal conditions (Fig. 15).^120^

3.12 is not emissive in diluted THF solutions, but the addition of 40% water results in aggregate formation and switching on of a red phosphorescence (674 nm, Fig. 15). The highest *Φ* value (99.9% and *τ*_av_ = 38 ms) is obtained at 90% water. Crystals of 3.12 are characterized by a stacking of dimers-of-trimer (intermolecular *d*_Cu⋯Cu_ of about 2.98 Å, Fig. 15) and a large number of C–H⋯N and C–H⋯π interactions. Such chromophoric organization strongly impacts the aggregated photoluminescence due to the dominant role of cuprophilic interactions in the excited states and the reduced nonradiative transition through restricted movement of alkyl groups. In fact, the photophysical behavior in the crystalline state is very similar to that observed in THF/90% water solution (677 nm, *τ*_av_ = 35.7 ms, and *Φ* = 99.9%). Through DFT-TDDFT analysis, it was concluded that from the high-lying dimer S_11_, populated upon excitation, IC to the lowest singlet state S_1_ (^1^LMMCT/^1^LC) occurs, then ISC to the T_10_ (^3^LMMCT/^3^LC) state and, finally, IC to T_1_ (^3^LMMCT/^3^LC), from which red phosphorescence is eventually produced.

**Fig. 16:**
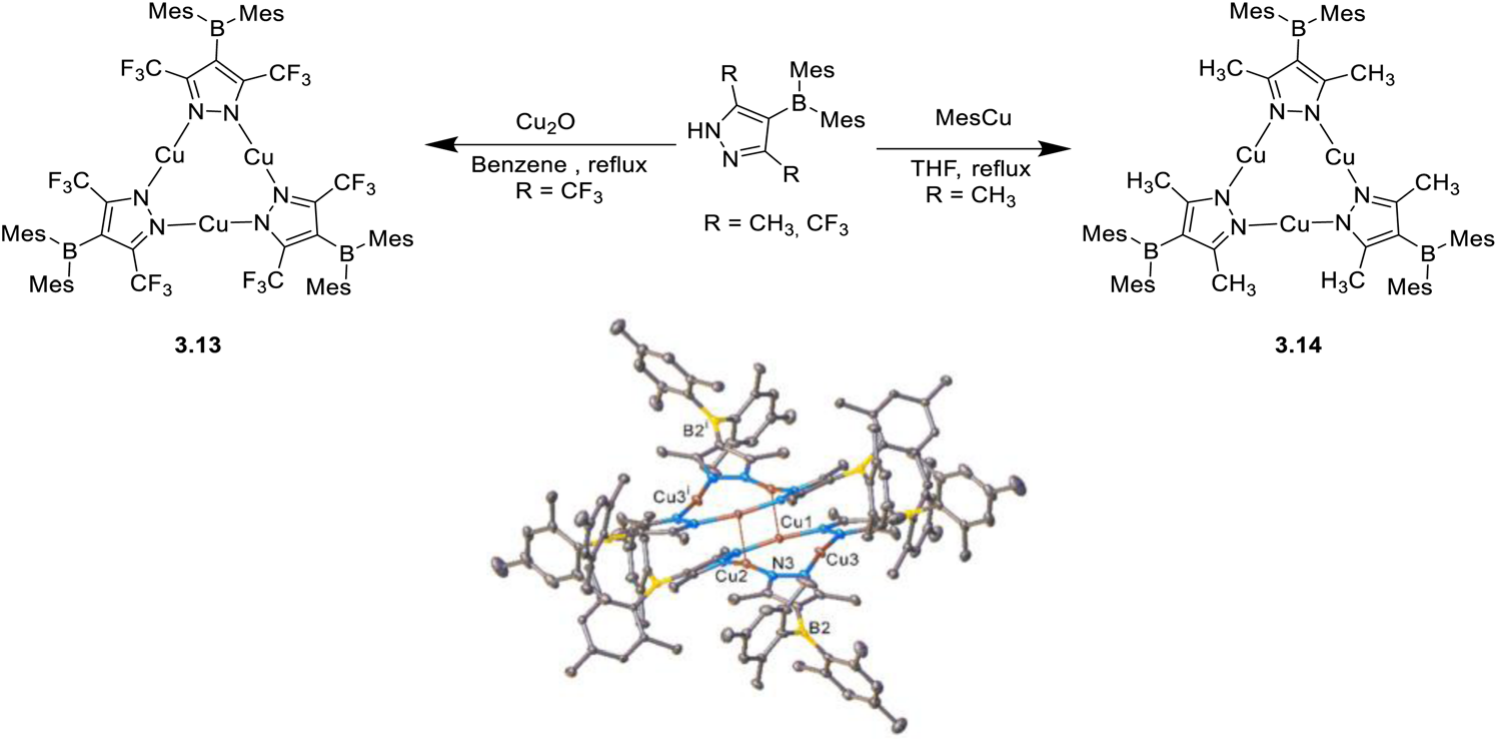
Synthesis of 3.13 and 3.14 (left) and crystal packing of 3.14 (right). Adapted with permission from ref. 121. Copyright 2023, The Royal Society of Chemistry.

Functionalization of pyrazole in the peripheral position with sterically demanding groups can effectively drive the supramolecular assembly. In this regard, Vanga *et al.* characterized two Cu(i)-CTCs with sterically hindered dimesityl-boron substituted pyrazoles, 4-Mes_2_B-3,5-(R)_2_Pz, namely 3.13 and 3.14 with R = CF_3_ and CH_3_, respectively.^121^3.13 and 3.14 were synthesized by reacting, respectively, Cu_2_O and mesityl copper(i) with the corresponding pyrazole under reflux. SCXRD studies of the two revealed a dimer-of-trimer arrangement with an intertrimer *d*_Cu⋯Cu_ of about 3.18 Å for 3.14 and a discrete molecular structure for 3.13 due to the steric repulsion between mesityl and CF_3_ groups (Fig. 16). In solution, 3.13 and 3.14 displayed similar blue fluorescence (*τ* = 0.9 and 3.6 ns, respectively) of ILCT origin. In the solid state, the presence, for 3.14, or absence, for 3.13, of cuprophilic interaction deeply affect the photoluminescent properties. In fact, 3.13 displays blue fluorescence (384 nm and 1.39 ns), while 3.14 exhibits red phosphorescence (704 nm and 16.4 μs), ascribable to the dimeric units.

**Fig. 17:**
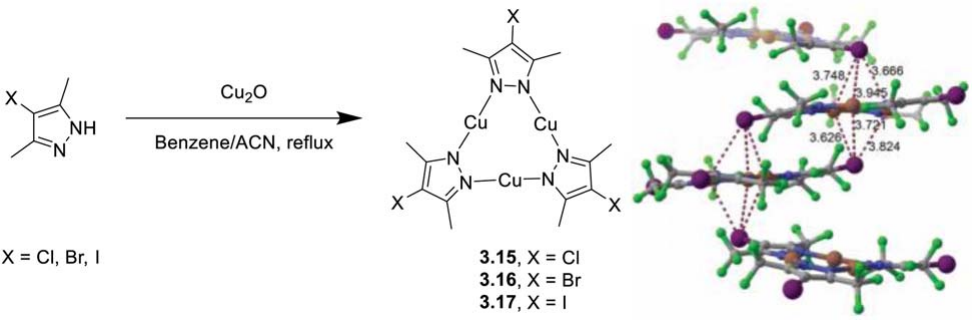
Synthesis of 3.15–17 (left) and crystal packing of 3.17 (right) with I⋯Cu_3_⋯I cluster interactions highlighted. Adapted with permission from ref. 123. Copyright 2023, The Royal Society of Chemistry.

In 2023, Lu *et al.* implemented their previous results on highly fluorinated bromo- and chloro-Cu(i) CTCs of general formula Cu_3_[4-X-3,5-(CF_3_)_2_Pz]_3_ with their iodo analogue (3.15, 3.16 and 3.17, respectively) prepared through a one pot reaction (Fig. 17).^122,123^ The photoluminescence of 3.17 was compared with that of 3.15 and 3.16, and the differences were rationalized through analysis of molecular and supramolecular features.

Crystal structure of 3.17 is characterized by I⋯Cu_3_⋯I double-capped interactions, which are stronger than the single-capped Cl/Br⋯Cu_3_ ones found in 3.15 and 3.16. In contrast to yellow phosphorescent 3.15 and 3.16 (about 575 and 590 nm, respectively), crystals of 3.17 are not emissive at r.t. but quite so at 77 K with phosphorescence centered at 570 nm (*τ* = 63.6, 68.7 and 72.4 μs for 3.17, 3.15 and 3.16, respectively). Through DFT/TDDFT calculations, the 77 K phosphorescence of 3.17 was related to the peculiar I⋯Cu_3_⋯I double-capped structure, resulting in an intertrimer through-space ^3^MLCT state. Room temperature quenching of 3.17, on the other hand, was explained by the stronger SOC value, which results in the acceleration of non-radiative decays.

**Fig. 18:**
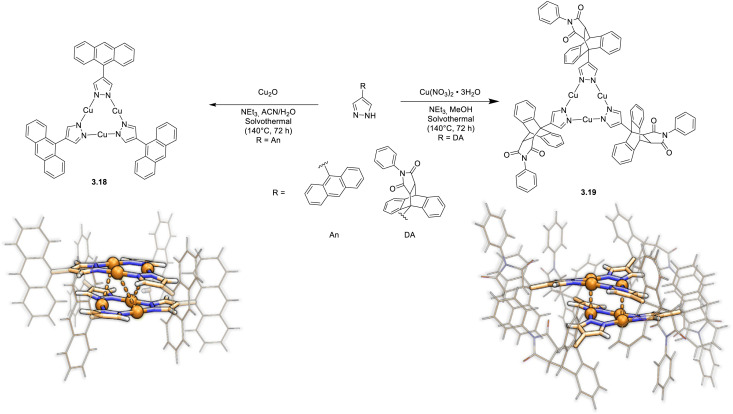
Synthesis and crystal packing at 100 K of 3.18 (left) and 3.19 (right). Adapted with permission from ref. 124. Copyright 2024, The Royal Society of Chemistry.

In 2024, Xiao *et al.* reported two Cu_3_Pz_3_ whose ligand was either 4-anthracenylpyrazole (An), 3.18, or its product of Diels–Alder [4 + 2] cycloaddition with N-phenylmaleimide (DA), 3.19 (Fig. 18).^124^3.18 and 3.19 were prepared by reaction of the corresponding ligand with either Cu_2_O or Cu(NO_3_)_2_, respectively, under solvothermal conditions in mixed solvents and Et_3_N.^124^ Crystals of both compounds show a dimeric unit with cuprophilic intertrimer interactions stronger for 3.19 with respect to 3.18 (intermolecular *d*_Cu⋯Cu_ of about 3.66 and 2.95 Å for 3.18 and 3.19, respectively).

Both compounds are emissive at r.t. in the solid state. In particular, 3.18 displays excitation dependent double fluorescence (with maxima at 570 and 520 nm) assigned, with the support of DFT/TDDFT calculations, to monomer and dimer ^1^LC states. 3.19 is a dual emitter characterized by one ligand-based fluorescence (450 nm, 1.03 ns) and one metal-sensitized ligand-localized phosphorescence (650 nm, 14.9 μs).

**Scheme 30:**
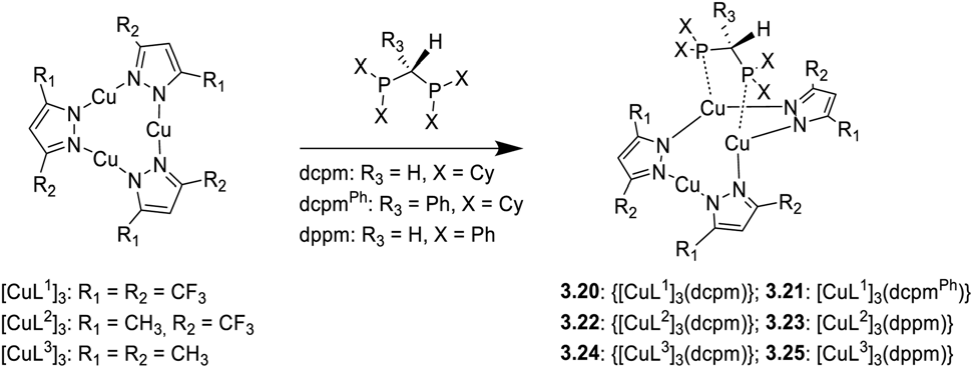
Synthesis of 3.20–25.

Baranova *et al.* decorated trinuclear copper(i) pyrazolates with short-bite phosphorus-containing ligands, as reported in Scheme 30.^125^

3.20–25 share similar structural arrangements, where the planar Cu_3_N_6_ geometry is lost due to the phosphine coordination with two Cu ions, resulting in the disruption of intertrimer cuprophilic interactions. In diluted DCM deaerated solution, they all emit phosphorescence in the 523–533 nm interval, with lifetimes ranging from 0.05 to 0.47 μs, assigned to ^3^MLCT–^3^LLCT states, as supported by DFT/TDDFT calculations. In the solid state, deactivation from MC excited states results in emission in the 500–585 nm range and lifetimes from 15 to 60 μs which, according to variable temperature experiments, was assigned to TADF.

**Scheme 31:**
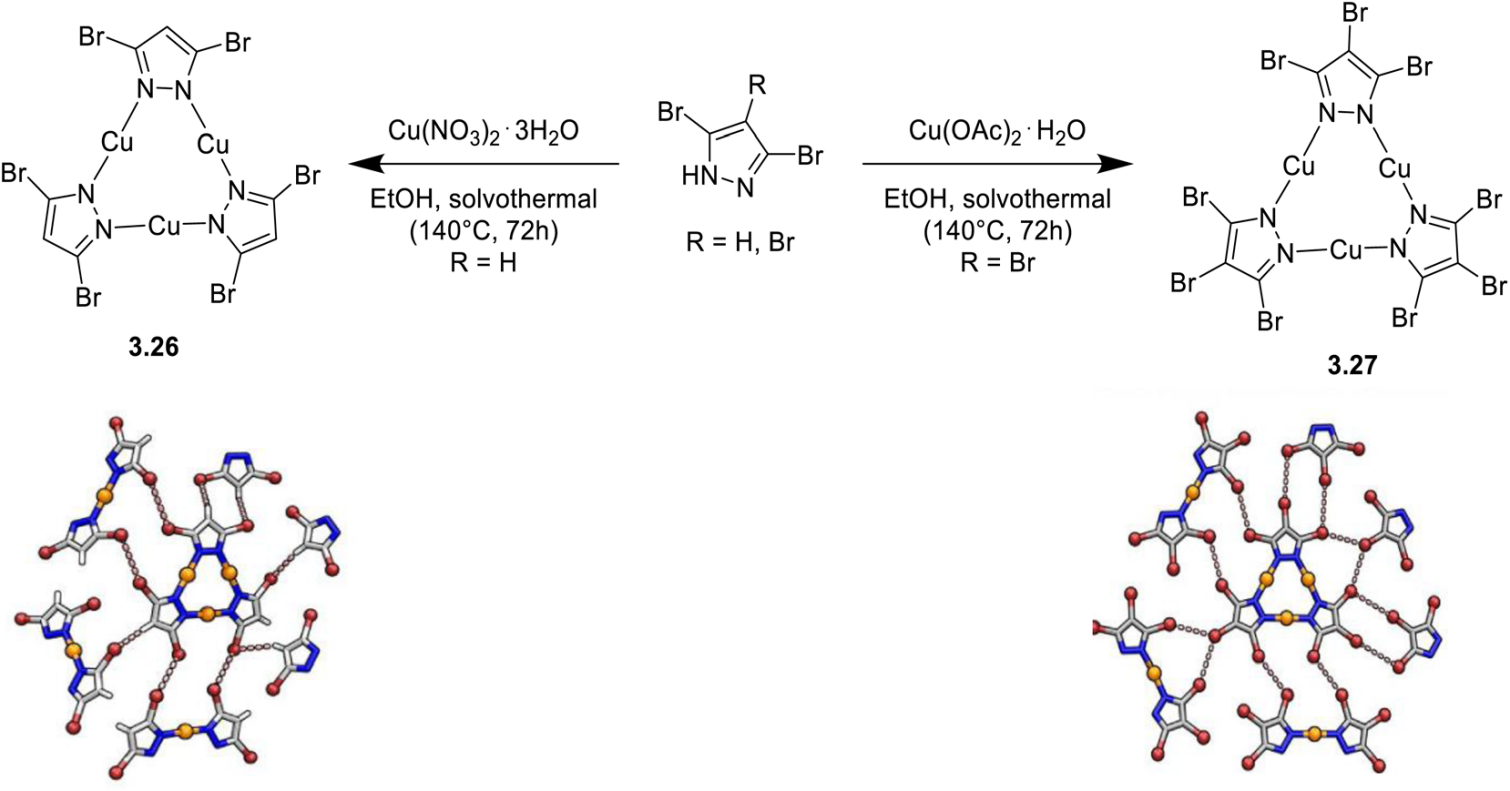
Synthesis of 3.26 and 3.27, and halogen bonding motifs in their crystal structure. Adapted with permission from ref. 126. Copyright 2025, American Chemical Society.

Huang *et al.*^126^ reported two bromo-pyrazolate-based Cu(i)-CTCs, 3.26 and 3.27, synthesized by a solvothermal reaction starting from Cu(NO_3_)_2_·3H_2_O and Cu(OAc)_2_·H_2_O, respectively (Scheme 31). The presence of six (for 3.26) or nine (for 3.27) bromine atoms leads to the formation of a halogen bond (XB) Br⋯Br network in their crystalline structure, leading to XB-dominated excimers, less distorted than the usual Cu⋯Cu ones.

**Fig. 19:**
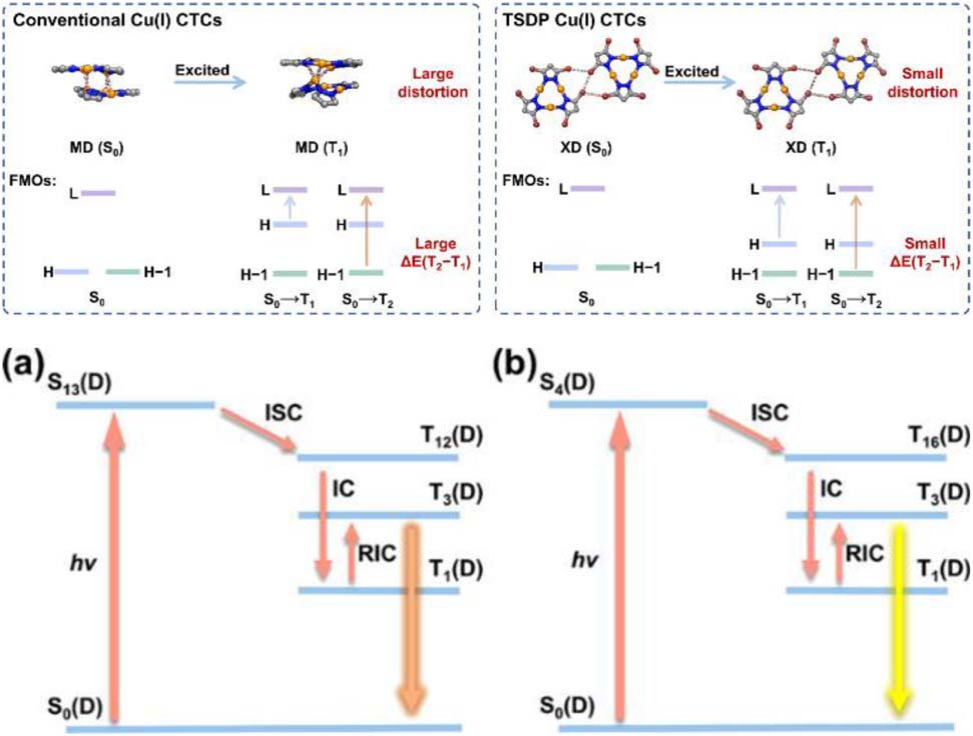
Top: proposed strategy for achieving TSDP behavior in Cu(i) CTCs. Left: conventional Cu(i) CTCs with Cu⋯Cu bonded dimers, highlighting significant excited-state distortions and large Δ*E*(T_2_ − T_1_). Right: TSDP Cu(i) CTCs with halogen-bonded dimers, highlighting less excited-state distortions and small Δ*E*(T_2_ − T_1_). Bottom: schematic representation of photophysical properties of XB dimers of 3.26 (a) and 3.27 (b). Abbreviations: S_0_ = ground state, T_1_ = lowest-energy triplet excited state, T_2_ = second-lowest-energy triplet excited state, FMOs = frontier molecular orbitals, H = HOMO, L = LUMO, MD = metal–metal-bonded dimer, XD = halogen bonded dimer, and S_*i*_(D) and T_*i*_(D) = singlet and triplet states, respectively, computed for the XB dimers. Reproduced with permission from ref. 126. Copyright 2025, American Chemical Society.

In this regard, SCXRD revealed, for both compounds, long intermolecular Cu⋯Cu distances (the shortest ones being 3.727 Å for 3.26 and 5.432 Å for 3.27), granting a nearly planar configuration of the Cu_3_Pz_3_ core and strong XB interactions between adjacent Cu(i) CTC molecules. Geometry optimization of dimeric fragments of both compounds revealed that they maintain their planarity in S_0_, T_1_, and T_2_ states, probably due to the strong XB interactions. Solid state samples of 3.26 and 3.27 display at r.t. broad orange and yellow phosphorescence, associated with XB-dominated excimers, centered at 626 nm (25.13 μs and *Φ* = 99.9%) and 596 nm (36.10 μs and *Φ* = 69.4%), respectively, and ascribed to radiative decay from an MMCT state for both complexes, with minor LMMCT and ILCT components, as indicated by theoretical studies. Variable temperature photophysical analysis unveiled an unusual negative thermal quenching (NTQ) behavior, that is, an increase in emission intensity with temperature. In addition, bathochromic shifting upon cooling to 77 K (631 and 567 nm for 3.26 and 3.27, respectively) and hypsochromic trend upon heating up to 400 K (620 and 530 nm, respectively) were observed. Moreover, 3.27 revealed dual emission below 100 K with the appearance of HE phosphorescence (about 450 nm) attributed to a molecular emission from an intramolecular Cu–Cu excited state. DFT/TDDFT calculations performed on 3.26 and 3.27 monomers, XB dimers and metal-bonded dimers, together with those performed on other common Cu(i)-CTCs and their metal-bonded dimers, allowed the exclusion of a TADF mechanism to explain the observed photoluminescence, due to the high singlet–triplet energy splitting (larger than 0.7 eV), besides the small lifetime increase observed upon cooling. A thermally stimulated delayed phosphorescence (TSDP) mechanism, involving emission from a higher triplet state, was instead invoked for 3.26 and 3.27, as supported by the small Δ*E*(T_2_ − T_1_) values computed for their XB dimers (0.031 and 0.052 eV, respectively), much smaller than those of the metal-bonded dimers, ranging from 0.162 to 0.627 eV (note that T_1_ and T_2_ refer to almost degenerate states T_1_(D), T_2_(D) and T_3_(D), T_4_(D), respectively), computed for the dimers, and Δ*E*(T_2_ − T_1_) is the energy difference between T_3_(D) and T_2_(D)). The small Δ*E*(T_2_ − T_1_) of 3.26 and 3.27 was associated with the inhibited (through XB) excited state distortion compared to the severe one caused by the enhanced Cu⋯Cu interaction Fig. 19). The slight excited-state distortion in 3.26 and 3.27 results in reduced internal reorganization energy and vibrational relaxation, explaining their high *Φ*.

### 4.2 [Cu_3_X_3_]-type trimers

The Cu_3_X_3_ scaffold is hardly found in the literature as a sub-unit of luminescent coordination polymer,^127–136^ and it is even less common in discrete complexes, being the luminescent [Cu_3_I_3_(dppep)_2_] (dppep = 2-[2-(diphenylphosphino)ethyl]pyridine),^137^ the only compound of this type reported before 2020. In the few examples reported during 2020–mid-2025 (summarized in Table S5), the Cu_3_X_3_ core adopted very different arrangements according to the ligand.

**Fig. 20:**
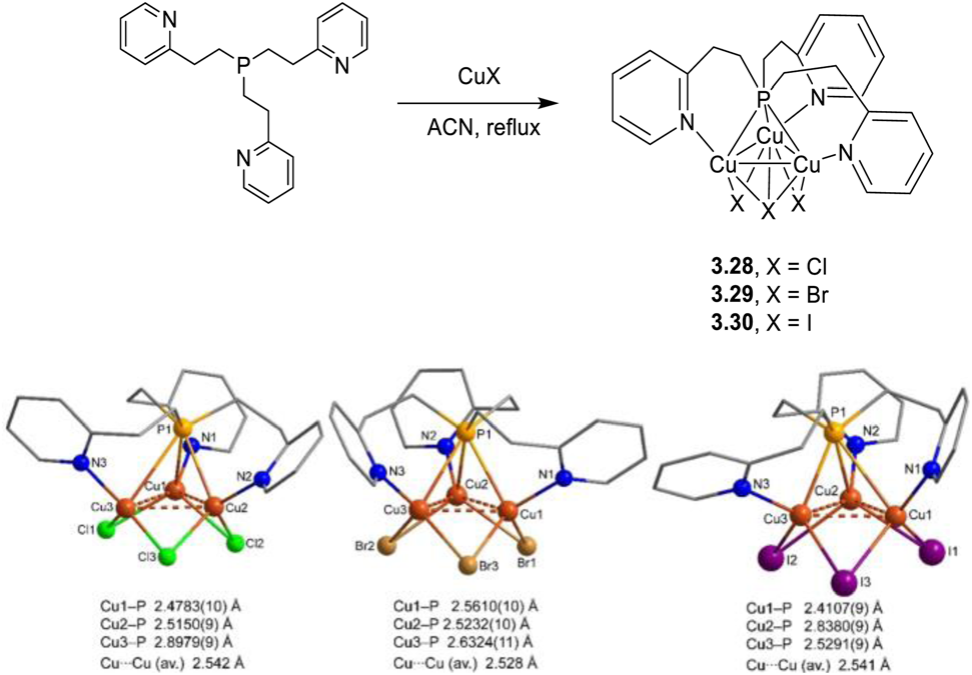
Synthesis (top) and crystal structures (bottom) of compounds 3.28–30, with the most important distances highlighted. Reproduced with permission from ref. 138. Copyright 2021, John Wiley and Sons.

In 2021, Artem'ev and coworkers reported the preparation and characterization of a series of [Cu_3_(μ_2_-X)_3_L] complexes (X = Cl, Br, or I, 3.28, 3.29, and 3.30, respectively, Fig. 20), where the tris[2-(2-pyridyl)ethyl]phosphine acts as the first example of a triply bridging phosphine ligand.^138^ The μ_3_-P-bridging pattern formed with the three copper atoms of the crown-shaped Cu_3_X_3_ unit was confirmed by both SCXRD studies, revealing Cu–P distances significantly shorter than the sum of Cu and P vdW radii, and DFT calculations, including Natural Bond Orbital Analysis, Natural Energy Decomposition Analysis, and Quantum Theory of Atoms In Molecules techniques, which evidenced a large charge-transfer contribution to the bonding between the phosphorus atom and the three Cu(i) atoms.

**Fig. 21:**
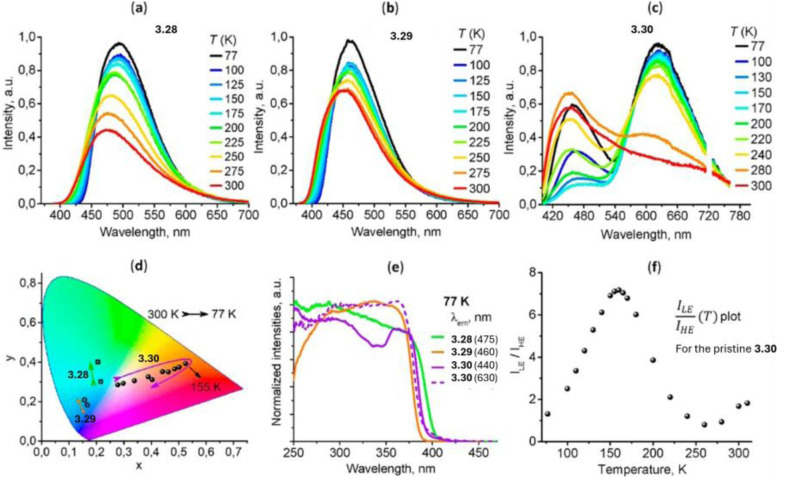
(a–c) Temperature-dependent emission spectra of pristine 3.28 (a), 3.29 (b), and 3.30 (c), recorded at *λ*_ex_ = 360 nm; (d) change in the emission chromaticity of pristine 3.28–30 upon cooling down from 300 to 77 K (*λ*_ex_ = 360 nm); (e) excitation spectra for pristine 3.28–30 at 77 K; (f) temperature dependence of the HE to LE emission band integral ratio (*I*_LE_/*I*_HE_) of pristine 3.30 (*λ*_ex_ = 360 nm). Reproduced with permission from ref. 138. Copyright 2021, John Wiley and Sons.

Moreover, strong cuprophilic intratrimer interactions were highlighted (*d*_Cu⋯Cu_ = 2.48, 2.56 and 2.57 Å, for 3.28, 3.29 and 3.30, respectively, Fig. 20 bottom). The three compounds possess in the solid state dual phosphorescence through a HE (of ^3^(M + X)LCT character) and a LE (of ^3^CC origin) emission with temperature- and excitation-dependent relative intensity and remarkable overall *Φ* (56, 100 and 50% for 3.28, 3.29 and 3.30, respectively, Fig. 21a–c). The photoluminescence of 3.28 and 3.29 is dominated, at r.t., by the HE emission (at 475 and 453 nm, respectively), while the LE one appears only at low temperature, manifesting through a 8–20 nm red shift upon cooling from 300 to 77 K. On the other hand, for 3.30, both components are visible (at 442 and 613 nm) even at r.t., and their relative intensity varies with temperature resulting in an intriguing fully reversible thermochromism. At 300 K and 300 nm excitation, the spectrum of 3.30 is dominated by the HE component resulting in its bluish-white emissive color; at about 160 K, the intensity of the LE component becomes predominant, and the emission color turns into orange; upon further cooling to 77 K, the HE intensity grows again restoring the bluish-white color (Fig. 21e). Based on the photophysical data and the results of DFT/TDDFT calculations, the HE and LE bands of 3.28–30 were assigned to phosphorescence from the ^3^(M + X)LCT and the ^3^CC states, respectively, the latter being populated from the ^3^(M + X)LCT one by overcoming a halogen-dependent energy barrier (Fig. 21).

**Fig. 22:**
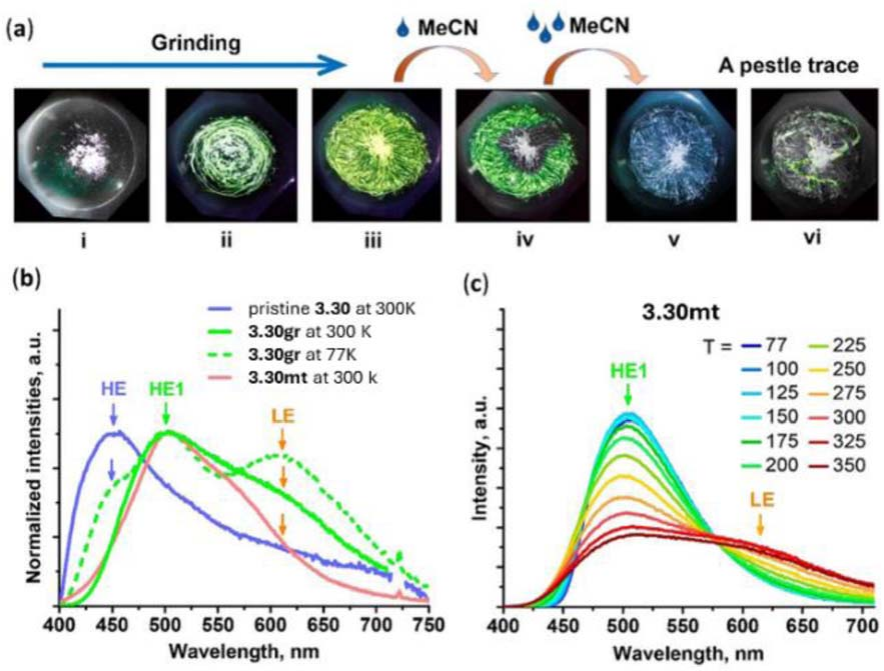
Mechanochromic luminescence of 3.30: (a) images obtained under a UV lamp: (i)–(iii) changing emission color of the intact microcrystals 3.30 upon grinding with a pestle; (iv and v) a reversion to the bluish-white emission by drop-wise treatment of 3.30gr (ground 3.30) with MeCN; (vi) scratching the powder 3.30 formed with a pestle. (b) Emission spectra of 3.30, 3.30gr, and 3.30mt (melted 3.30) at 300 and 77 K (*λ*_ex_ = 360 nm); (c) emission spectra of 3.30mt recorded in the 77–350 K window (*λ*_ex_ = 360 nm). Reproduced with permission from ref. 138. Copyright 2021, John Wiley and Sons.

Interestingly, upon gently grinding, the emission of 3.30 turns green (HE1 at 505 nm, Fig. 22), showing, at 77 K, the three distinct components (HE, HE1 and LE, Fig. 22b), and reverts to the original color upon treatment with a few drops of ACN. Moreover, the emission spectrum of the completely amorphous (melted) phase at 300 K displays overlapping bands of the dominant HE1 and LE components, while cooling to 77 K completely suppresses the LE band (Fig. 22c).

**Fig. 23:**
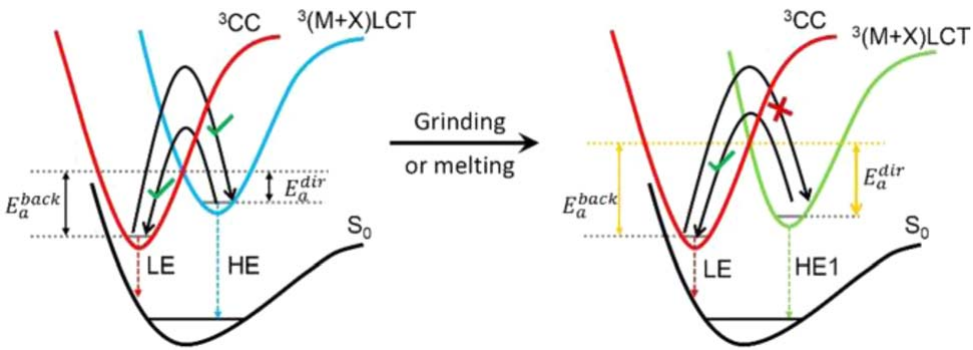
Simplified diagrams explaining the thermo- and mechanochromic luminescence of pristine (left) and fully amorphized (right) 3.30. *E*^dir^_a_ and *E*^back^_a_ represent the energy barriers for the direct and reverse population, respectively, from the ^3^(M + X)LCT to the ^3^CC states. Reproduced with permission from ref. 138. Copyright 2021, John Wiley and Sons.

The stimuli-responsive photoluminescence of 3.30 is explained according to the mechanism simplified in Fig. 23.

The ^3^CC state is thermally populated from the ^3^(M + X)LCT one already at 77 K through a small energy barrier (*E*^dir^_a_ of about 220 cm^−1^) resulting in the appearance of both LE and HE emissions (Fig. 23 left). The higher thermal population of the ^3^CC state at 160 K is at the basis of the observed increased ILE/IHE ratio (Fig. 21f), which instead decreases through further warming (160–300 K) due to the reversibility of conversion between ^3^(M + X)LCT and ^3^CC states. On the other hand, grinding or melting stabilize the ^3^(M + X)LCT state, resulting in a red shift of the HE emission towards HE1 (Fig. 23 right). The consequent increase of the energy barrier from the ^3^(M + X)LCT to the ^3^CC state (*E*^dir^_a_ of about 570 cm^−1^) is at the basis of the lack of LE emission at low temperature. In fact, thermal population of the ^3^CC state from the ^3^(M + X)LCT one occurs only above about 180 K. The amorphization induced stabilization of the ^3^(M + X)LCT state also increases the *E*^back^_a_ barrier, so that the reverse ^3^CC-^3^(M + X)LCT population does not occur even at 350 K.

**Fig. 24:**
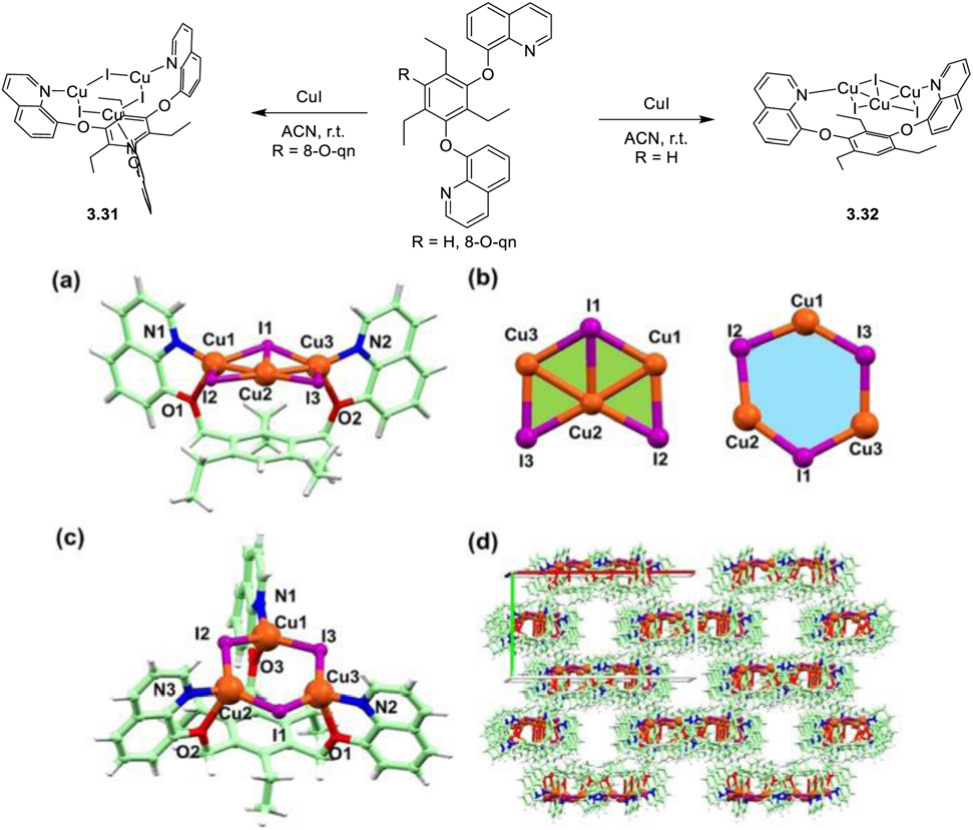
Top: synthesis of 3.31 and 3.32; bottom: SCXRD structure of 3.32 (a); coordination modes in the Cu_3_I_3_ cores of 3.32 (left) and 3.31 (right) (b); SCXRD structure of 3.31 (c) and its crystal packing viewed along the *c*-axis (d). Color codes: brown, Cu(i); purple, iodide; blue, nitrogen; red, oxygen; green, carbon; white, hydrogen. Reproduced with permission from ref. 139 Copyright 2022, American Chemical Society.

In 2022, Yang *et al.* reported two host–guest-encapsulated Cu_3_I_3_ supramolecular architectures [(Cu_3_I_3_)L] (3.31 and 3.32) using, respectively, a *syn*-conformer of flexible tripodal or dipodal quinoline (qn)-based ligands (Fig. 24).^139^

3.31 crystallizes in a propeller-like structure, with the Cu_3_I_3_ core in a chair-like arrangement and long Cu⋯Cu distances (4.05–4.31 Å), completely trapped within an ultrasmall cavity formed by the tripodal ligand (Fig. 24c and b, right). The complex is stacked into a highly ordered 3D network through weak π⋯π and C–H⋯π interactions (Fig. 24d). On the other hand, 3.32 displays a swallow-shaped Cu_3_I_3_ cluster core where the three Cu(i) atoms interact through intramolecular cuprophilic interactions (*d*_Cu⋯Cu_ = 2.53 and 2.56 Å, Fig. 24a and b, left). 3.31 and 3.32 display a broad emission in both diluted ACN solution (at 508 and 398 nm, respectively) and in the solid state (at 628 nm, 0.24 μs and 674 nm, 5.09 μs, respectively). Through DFT/TDDFT calculations, the observed emissions were assigned to radiative decay through ^3^MLCT states, the LUMO being composed of a mixture of dπ(Cu) orbitals and iodide π-orbitals, and the HOMO centered on the π* orbitals of the qn group.

**Scheme 32:**
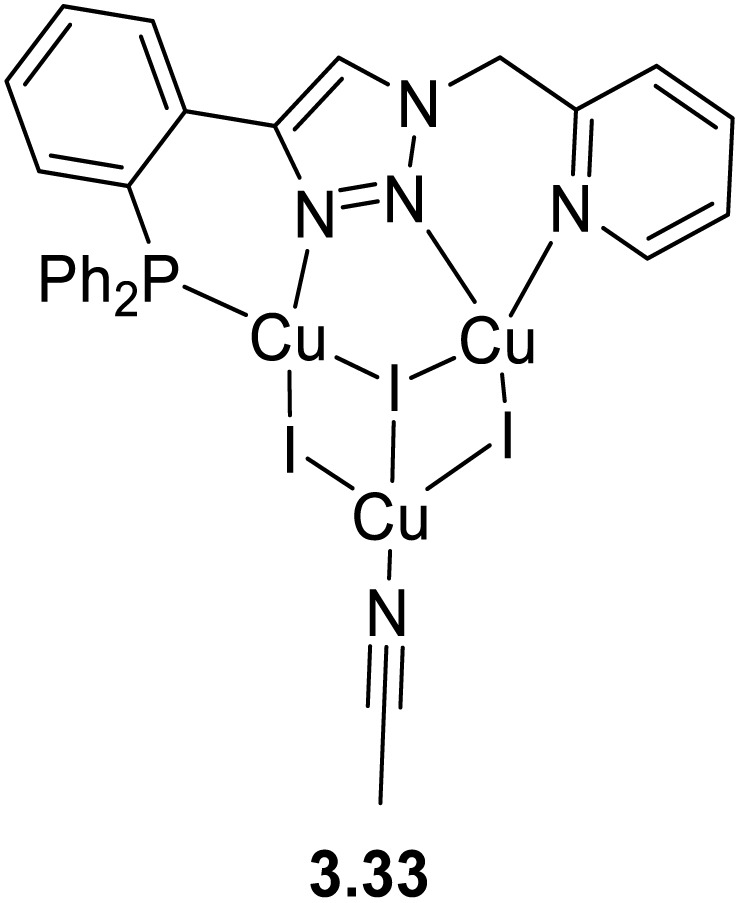
Chemical structure of 3.33.

In 2024, Balakrishna *et al.*,^140^ in preparing and characterizing a series of cationic Cu(i) derivatives containing a triazole-based ligand, namely *o*-Ph_2_P(C_6_H_4_)C(CH)-1,2,3-N_3_(CH_2_)(Py), having four available coordinating sites, also isolated the neutral trimeric complex 3.33, either by a one pot reaction between the ligand and CuI or through a cationic mononuclear intermediate (Scheme 32).

SCXRD revealed the presence of a Cu_3_I_3_ unit with two μ_2_- and one μ_3_-iodide and having two Cu atoms bridged by the tetradentate ligand and the third one coordinated to an ACN molecule. The Cu⋯Cu distances span from 2.86 to 3.55 Å. 3.33 displays phosphorescence at 488 nm (*Φ* = 68% and *τ* = 447 μs) which, with the support of DFT/TDDFT calculations, was assigned to deactivation from a T_1_ level of ^3^MLCT character, with the HOMO localized on the CuI cluster and the LUMO on the phenyl ring.

**Scheme 33:**
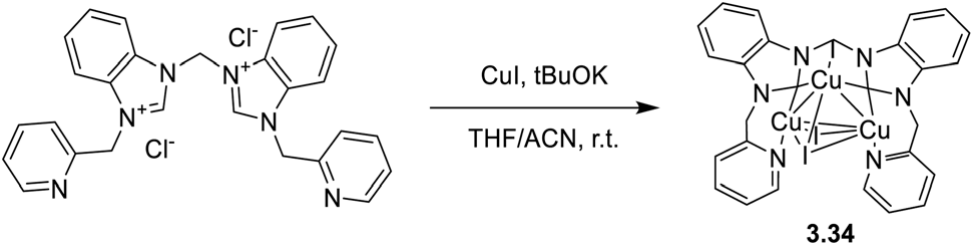
Synthesis of 3.34.

As mentioned before, the stabilization of Cu_*n*_X_*n*_ clusters can also occur with the aid of NHC ligands. In this regard, Yang *et al.* designed a family of picolyl-substituted NHC ligands to construct Cu_*n*_I_*n*_ clusters (see Section 5) featured by photoluminescence. By properly fashioning the 2-picoline precursor, the authors prepared and crystallized the trinuclear derivative Cu_3_I_3_(bisNHC^Me^), 3.34 (Scheme 33).^94^

As revealed by SCXRD analysis, each NHC ligand interacts with two Cu(i) atoms through one short σ-bonding involving hybridized C(sp^2^) and one long π-interaction with non-hybridized C(p_*z*_), with average distances *d*_Cu–C_ = 1.97 and 2.34 Å, respectively, and *d*_Cu⋯Cu_ = 2.57 Å. Moreover, the Cu coordination sphere is completed by iodine which links metal center in three different ways: one terminal coordination and two emerging modes comprising one μ_2_-I and one μ_3_-I bridge. The complex is non-emissive in solution but emissive in the solid state through 590 nm phosphorescence (*τ*_av_ = 30.2 μs and *Φ* = 31%) mainly assigned, with the support of DFT/TDDFT analysis, to a ^3^MLCT T_1_ level. Indeed, the HOMO is localized on the CuI cluster, while the LUMO on the Py ring. The TADF mechanism is excluded due to the slight increase of lifetimes at 77 K (33 μs), not enough to justify this mechanism.

## 5. Tetrameric complexes

**Scheme 34:**
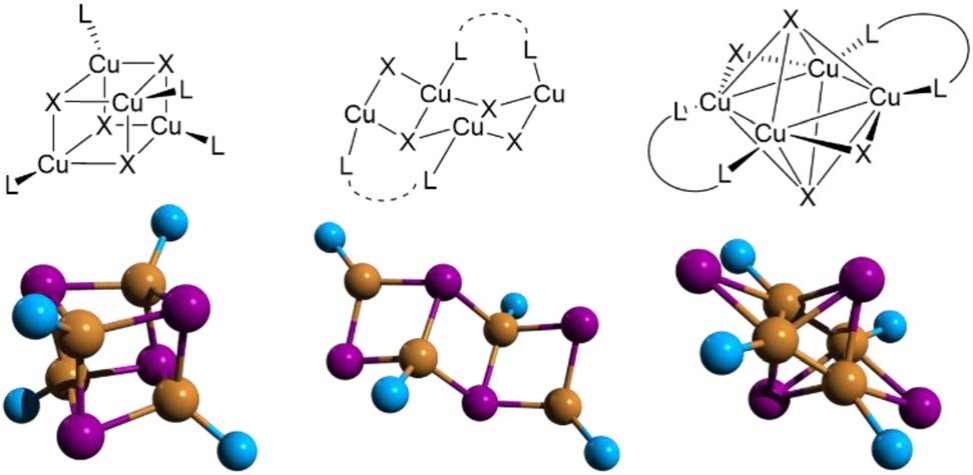
Possible Cu_4_X_4_ structures.

Among the Cu_4_ tetranuclear clusters, the most common structures are based on the Cu_4_X_4_ unit (X = I, Br, Cl) which can show the cubane (Scheme 34 left), the chair-like or stair-step (Scheme 34 center) and, more rarely, the octahedral motif (Scheme 34 right), the latter also displaying the ‘fox-type’ and ‘butterfly’ variants. The cubane unit consists of two Cu_4_ and X_4_ overlapping tetrahedra with each face capped, respectively, by three-coordinated X or Cu atoms. It can also be viewed as a pair of Cu_2_X_2_ fragments with Cu–Cu axes oriented perpendicular to each other.

The tetra-coordination around the metal center is completed generally by a monodentate pnictogen (N, P, As, and Sb) ligand, resulting in [Cu_4_X_4_L_4_] structures, or less commonly by bidentate ligands (*e.g.* bisphosphines) generating [Cu_4_X_4_L_2_] complexes. Both [Cu_4_X_4_L_4_] and [Cu_4_X_4_L_2_] cubane-like clusters can be synthesized by one pot reactions in solution, or by post-synthetic ligand exchange.

The Cu_4_X_4_ chair-like motif is formed by two Cu_2_X_2_ units joined along the Cu–X edges, and it is preferentially obtained from bulky ligands due to its less compact structure. It has been observed with bidentate (*e.g.* N^N-,^141–144^ P^P-,^145^ P^N-bidentate^146,147^ and more rarely, monodentate^148–150^ or N^N^N-tridentate^132,151–154^ ligands). The Cu(i) atoms generally display tetrahedral geometry except for some cases where both tetrahedral and trigonal coordination are observed,^155–159^ according to the coordinating ability of the ligands involved. These compounds are typically obtained by one pot reactions in solution reactions under mild conditions.

In the octahedral Cu_4_X_4_ motif, the copper atoms are arranged in a planar or pseudo-planar, more or less stretched parallelogram, with μ_4_-halogenides above and below the copper plane and μ_2_-halogenides bridging the Cu(i) on the two short edges of the parallelogram. These complexes are commonly supported by bidentate ligands, such as N^N-,^160^ P^N-,^161–169^ and P^P-donors^170,171^ which bridge the long Cu–Cu edges in an antiparallel or, more rarely, parallel fashion, but very few cases obtained with monodentate N-donor ligands have also been reported.^172,173^ Their number is much smaller than that of Cu_4_I_4_ cubane derivatives due to the lower stability of the octahedral core.^164^ They are generally obtained by reaction of the ligand with the appropriate amount of copper halide in solution. Their formation, with respect to more thermodynamically stable binuclear complexes, can be addressed by the ligand-to-metal molar ratio, under almost the same reaction conditions,^166^ or by using different (polar or apolar) solvents.^174^ Even more rare variants to the classical octahedral arrangement are the ‘fox-type’ and ‘butterfly’ geometries. In the former, the μ_2_-halogenides and bridging ligands are in *cis*- rather than in *trans*-positions, with the ligands still lying at the long side of the Cu_4_ parallelogram.^175^ In the latter, the four Cu atoms assume a butterfly disposition, so that, besides the two μ_2_-halogenides bridging adjacent copper atoms, two additional μ_2_-halogenides (instead of the μ_4_ ones) bridge opposing copper atoms from either side of the butterfly.^162^

**Scheme 35:**
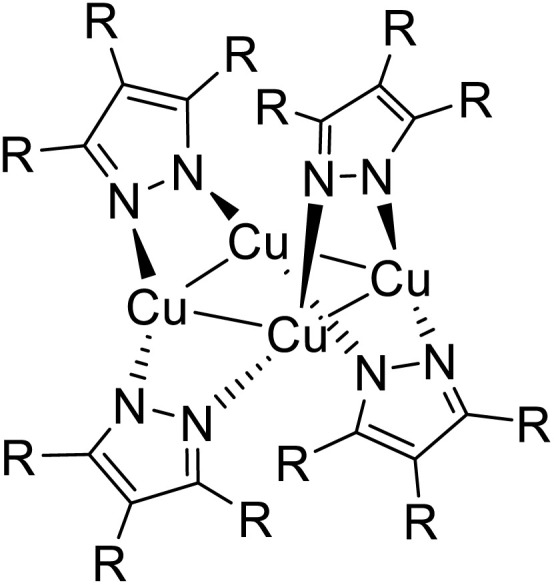
Cu_4_L_4_ structure.

Unlike Cu_4_X_4_ clusters, halogen-free tetranuclear Cu(i) compounds have been much less investigated. They are mainly supported by pyrazolate derivatives (Scheme 35) and can be obtained from CuX, Cu_2_O or [Cu(CH_3_CN)_4_]X (X = BF_4_ or PF_6_) with the sodium salt of differently substituted pyrazoles in the appropriate solvent. The different complexes are obtained by properly manipulating the substituents on the pyrazolyl ring. In general, they are formed preferentially, against the corresponding trinuclear Cu_3_Pz_3_ counterpart, using larger or more numerous substituents on pyrazole, to reduce the steric hindrance between adjacent pyrazolates.^176^ Structural studies on Cu_4_Pz_4_ complexes were initially performed mainly in view of their catalytic activity,^105,177–181^ and only more recently they have attracted further attention because of their photophysical properties.^176,182^

Different from trinuclear planar Cu_3_Pz_3_ structures, Cu_4_Pz_4_ complexes assume, in most cases, a saddle-shaped ‘basket’ structure, where copper atoms are two-coordinated in a nearly linear arrangement and are strictly or almost coplanar forming a square or rhombic array. The intramolecular Cu⋯Cu shorter distances are generally above 2.9 Å, becoming longer with increasing steric bulkiness of the ligand and do not change significantly by cooling the sample. The pyrazolate ligands lie alternately above and below the Cu_4_ plane, forming through its two nitrogen atoms a –[Cu–N–N]_4_– repeating unit in a cyclic arrangement, and therefore featuring a 12-MC-4 azametallacrown structure according to the nomenclature proposed by Mezei *et al.*^183^ A summary of the photophysical properties of the mentioned compounds is reported in Table S6.

### 5.1 Cu_4_X_4_L_*n*_ motifs

The first studies on the luminescence properties of [Cu_4_I_4_L_*n*_] cubane clusters (where L is Py or an amine-based ligand) were reported in the 70s^184,185^ and further extended in the 80s^186,187^ and 90s.^30,188–190^ Often, these compounds are characterized by high (>50%) values of *Φ*, sometimes exceeding 90%, and the presence of two distinct, non-equilibrated HE (at *ca.* 400–450 nm) and LE (at *ca.* 520–650 nm) emission bands, originating from two distinct triplet excited states, whose relative intensity varies with temperature. At r.t., the predominant LE band is attributed to ^3^CC transition, as supported by the observation that little to no LE emission originated for compounds with intramolecular Cu⋯Cu distances longer than the sum of the vdW radii of two Cu-atoms. Through calculations and experimental evidence,^191–195^ this transition is assigned to charge transfer from halogen to copper, followed by electronic and geometric reorganization within the inorganic core. The ^3^CC state is in fact characterized by significant distortion of the cluster geometry with respect to the ground state one, therefore generating LE emission with a large Stokes shift.

Upon cooling, the LE band of cubane clusters generally red shifts due to the shrinkage of the Cu_4_ tetrahedron. This effect, however, is offset or sometimes overwhelmed by the inhibition of the excited state relaxation in the rigid matrix at low temperature, a phenomenon known as rigidochromism, leading to blue-shift of the emission.^191^ The resulting trend depends on the outer environment, as determined by ligands and packing modes, which can exert a positive or negative ‘pressure’ on the central core, leading, respectively, to its contraction or expansion when lowering the temperature.^192^

The HE band, on the other hand, often outweighs the LE one at low temperature. It was associated with a mixed ^3^(M + X)LCT excited state, therefore leaving the cluster structure relatively unperturbed and providing, owing to its different nature with respect to the ^3^CC state, an alternative route to radiative deactivation.^191^ The ^3^(M + X)LCT transition energy clearly depends on the electronic structure of the ligand, offering a possible route towards full visible spectrum tunability of the emission color from blue to red, as demonstrated by a plethora of investigations. Besides temperature, the effect of size, shape, denticity and relative orientation of the ligands, as well as the rigidity of the medium (all factors affecting the structural distortion of the inorganic core), on the relative intensity of the two bands was also highlighted. Moreover, it was proposed that for some ligands (*e.g.* 2-(diphenylmethyl)pyridine), the two states responsible for the two emission bands can be in thermal equilibrium owing to a relatively low barrier separating the two excited states.^196,197^

In contrast to cubane compounds, Cu_4_X_4_L_*n*_ chair-like 0D clusters, first reported in 2000s,^153,159^ display only emission from ^3^XLCT and/or ^3^MLCT excited states, at positions strongly dependent on the ligand's nature, while no evidence of emission from the ^3^CC state is observed even for the few structures with Cu⋯Cu distances in the 2.65–2.80 Å range.^153^

In complexes with an octahedral Cu_4_X_4_ core, photophysically investigated only recently,^164^ the rigidity and steric hindrance of the ligands, together with the associated distortion of the Cu_4_X_4_ core, play a significant role in the emissive properties. These complexes display both short (even below 2.50 Å) and long (>2.80 Å) Cu⋯Cu distances, generally reflecting, in the solid state, in dual emission from both ^3^(M + X)LCT and ^3^CC excited states with associated thermochromic effects.^164,166,168^ In some cases, however, only one emission from the ^3^(M + X)LCT excited state, observed at HE^164^ or LE,^162^ or dual emission from two different ^3^(M + X)LCT excited states^168,169^ were observed. Single emission from ^3^ILCT or mixed ^3^ILCT/^3^(M + X)LCT excited states was also recognized in the presence of zwitterionic ligands.^160^ In these cases, the absence (or scarce importance) of the ^3^CC emission band, even in the presence of very short Cu⋯Cu distances, was ascribed to the presence of bulky and/or rigid ligands, impeding the structural relaxation of the ^3^CC excited state and therefore reducing the intensity of the associated emission.^164^ This hypothesis was confirmed by structural studies on octahedral complexes supported by rigid pyrazolate-type ligands, for which no emission from ^3^CC states was highlighted.^160^ These compounds, different from cubane clusters, do not show appreciable shortening of the Cu⋯Cu distances when lowering the temperature.

Systematic spectroscopic and computational studies on isostructural Cu_4_X_4_L_*n*_ clusters bearing different halogens (X = I, Br, and Cl) demonstrated their scarce influence on the luminescence properties. More precisely, while excitation profiles are nearly superimposable, the emission energies are only slightly red-shifted with increasing the size of the halogen.^162,196,197^

Copper(i) iodide, however, is generally preferred to bromide and chloride due to its better oxidation stability, as well as for the higher luminescence of its Cu(i) complexes with respect to bromide and chloride analogs.^174^

#### 5.1.1 Advancements during 2020–mid-2025

**Fig. 25:**
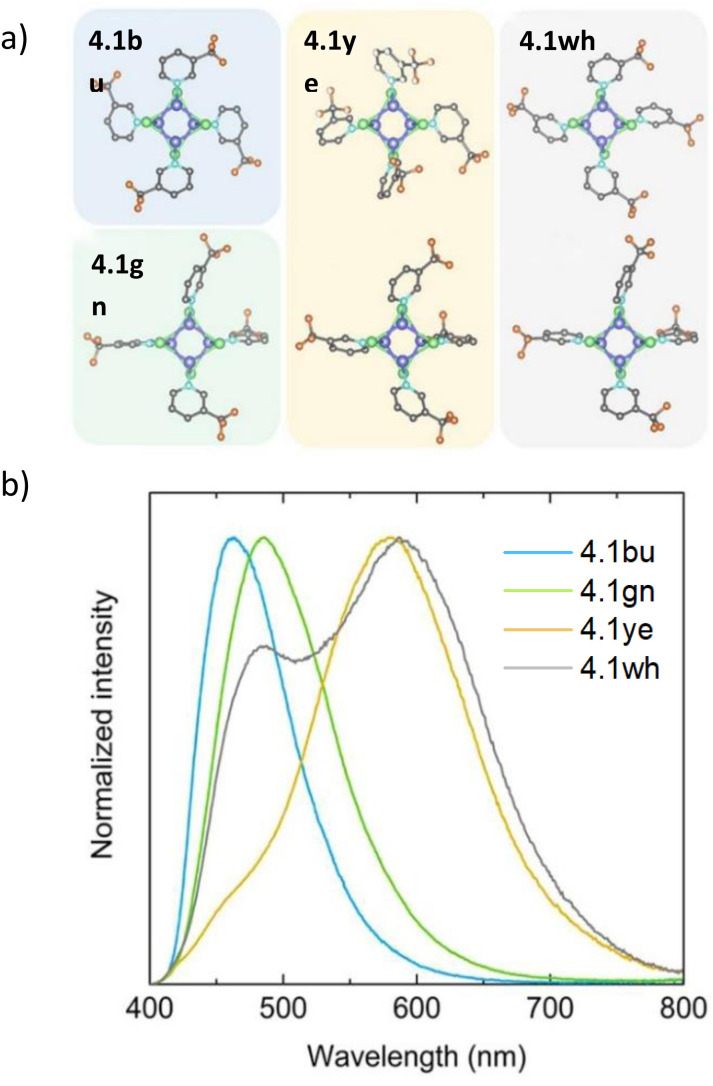
(a) Structures of basic units of 4.1bu, 4.1gn, 4.1ye and 4.1wh; (b) normalized emission spectra of compounds 4.1. Adapted with permission from ref. 198. Copyright 2024, American Chemical Society.

In recent years, several [Cu_4_X_4_L_*n*_] clusters with cubane, chair-like and octahedral motifs were prepared and photophysically characterized, making increasingly clear the role of the ligands not only in the emissive properties of the complexes but also in their structural arrangement. Concerning the first aspect, particularly intriguing is the case of four [Cu_4_I_4_L_4_] cubane polymorphs having 3-trifluoromethylpyridine as the ligand, which show either single or dual emission.^198^ The four polymorphs were obtained simultaneously by slow diffusion of ethanol solution of the ligand into KI saturated aqueous solution of CuI, varying the CuI : ligand ratio from 1 : 0.6 to 1 : 2.^198^ Only one of them (4.1wh) was prepared in the pure phase using the two components in a 1 : 4 ratio (Fig. 25a). The molecular conformation of the four polymorphs differs essentially for the orientation of the Py ring with respect to the cubane core, the latter characterized in all cases by Cu⋯Cu intramolecular distances slightly below two times the Cu vdW radius. The polymorphs pack in different space groups with weak π–π interactions and close F⋯F contacts, the shorter ones being tentatively described as rare examples of “Type-II” F⋯F halogen bonds.^199^ The different structural features of the four polymorphs result in different stability in air, possible single-crystal-to-single-crystal transformations and quite dissimilar photoluminescence properties.^199^ They exhibit blue (4.1bu), green (4.1gn), yellow (4.1ye), and white (4.1wh) light emission under UV excitation, all with near-unity *Φ* (Fig. 25b). Moreover, while 4.1bu and 4.1gn display in their excitation spectrum a single peak in the 460–490 nm range, dual emission is observed for 4.1ye and 4.1wh, whose LE, more intense peak is centered at about 580 nm and the HE one (which is a weak shoulder for 4.1ye) falls in the same range of 4.1bu and 4.1gn. The emissive features of 4.1wh correspond to pure white emission. DFT periodic calculations allowed assigning the emission of all 4.1 polymorphs to radiative decay from one (4.1bu and 4.1gn) or two (4.1ye and 4.1wh) ^3^CC states, whose energies depend on the distortion degree of the [Cu_4_I_4_] excited state geometry with respect to the ground state one, as dictated by the different constraints imposed by the ligand.

**Scheme 36:**
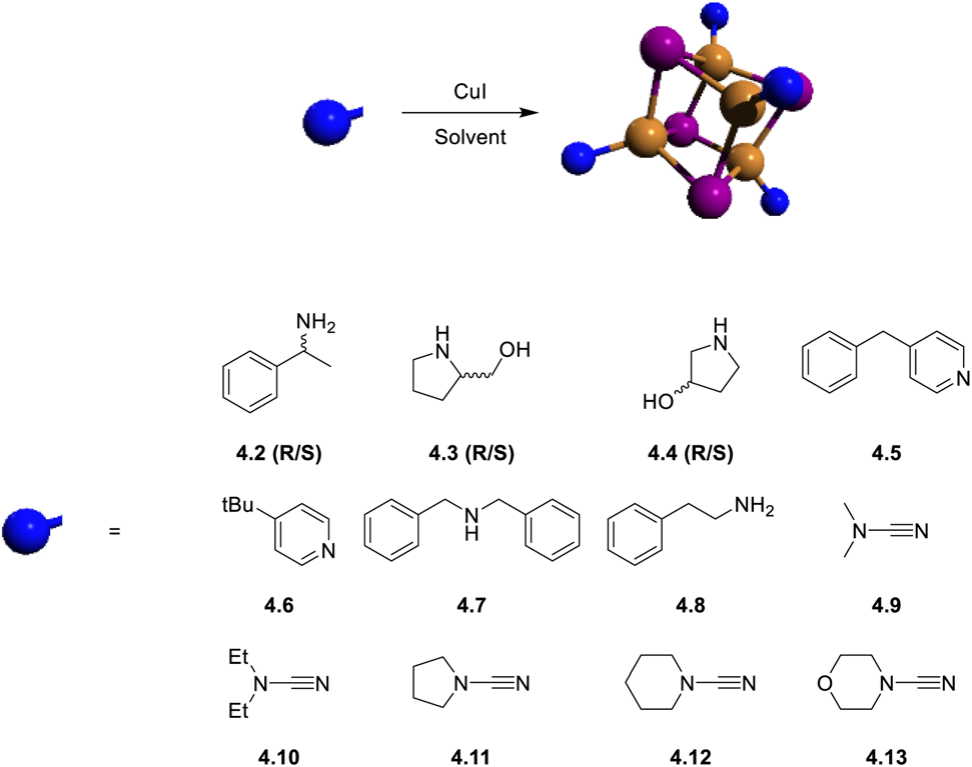
Synthesis and the exemplified structure of 4.2–13.

Single emission at low energy was recently reported for several [Cu_4_I_4_L_4_] cubane clusters with monodentate ligands such as *R*/*S*-methylbenzylammonium (4.2),^200^*R*/*S*-2-pyrrolidinemethanol (4.3) and *R*/*S*-3-hydroxypyrrolidine (4.4),^201^ all showing CPL activity, and 4-benzylpyridine (4.5), 4-*tert*-butylpyridine (4.6),^202^ dibenzylamine (4.7),^203^ and phenylethylamine (4.8) (Scheme 36).^204^

Based on both experimental evidence, *i.e.* long lifetimes (often >15 μs), a large Stokes shift (>1.2 eV) and a wide photoluminescent full width at half maximum (FWHM, >100 nm), and, in most cases, computational studies, emission of these compounds was assigned to radiative decay from a ^3^CC excited state in almost all cases (4.2, 4.3, 4.4, 4.7, and 4.8). Only for the series of cubane clusters based on dialkylcyanamide derivatives, NCNR_2_, with R_2_ = Me_2_ (4.9), Et_2_ (4.10), C_4_H_8_ (4.11), C_5_H_10_ (4.12), and C_4_H_8_O (4.13), a weak HE fluorescence was observed, in addition to the ^3^CC LE phosphorescence.^205^ On the other hand, for 4.5–6, characterized by slightly lower (0.64 and 0.95 eV, respectively) Stokes shift, DFT calculations indicated a ^3^(M + X)LCT excited state as responsible for their emission, though no structural data were reported to rationalize the different behavior of these compounds with respect to the other cubane clusters. Some of these complexes revealed very appealing properties useful for a range of applications. For example, 4.8 displays improved stability towards oxygen, light, and water, besides high *Φ* (68%), making the compound competitive with traditional perovskites and suitable for LED fabrication. Importantly, 4.5–6 and 4.7, featuring very high *Φ* (90% and more) at r.t., were successfully used to realize high-performance scintillation screens with high light yields, low detection limits, excellent radiation resistance and high spatial resolution, showing great potential in X-ray imaging applications. Moreover, 4.7 revealed NTQ behavior which has not originated from TADF, as proven by time-resolved measurements.^203^ This phenomenon was associated with exciton de-trapping processes from shallow defect states derived from the [Cu_4_I_4_] distortion, followed by radiative recombination resulting in enhanced emission. The efficiency of the process increases with temperature, explaining the observed NTQ effect.

**Scheme 37:**
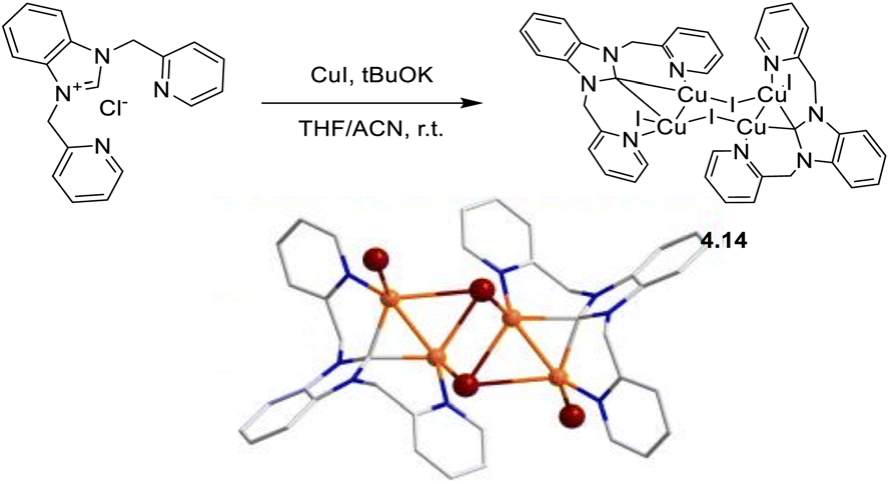
Synthesis and crystal structure of 4.14. Adapted with permission from ref. 94. Copyright 2024, The Royal Society of Chemistry.

A chair-like tetramer was reported in 2024 by Yang *et al.*,^94^ who obtained clusters of nuclearity equal to 2 (2.54), 3 (3.34) or 4 (4.14) by subtle changes in the substituent groups in NHC ligands.^94^ In the tetranuclear complex, 4.14, obtained from NHC bearing two picolyl groups (Scheme 37), the Cu⋯Cu distance (2.44 Å on average) is shorter than those found in the di- and trinuclear analogues (2.51 and 2.57 Å, respectively, on average) and significantly shorter than the sum of the vdW radii of Cu atoms, suggesting strong cuprophilic interaction. In spite of this, the HOMOs and LUMOs obtained through DFT calculations are mostly located on the inorganic centers and on the pyridyl groups, respectively, indicating a MLCT character for the electronic transitions, as obtained for the di- and trinuclear analogues. Interestingly, though the HOMO–LUMO gap decreases in the order di- > tri- > tetranuclear species, the maximum emission of the latter in the solid state (at 526 nm) is slightly red-shifted only with respect to the dinuclear complex.

The large red-shift observed for the trimer (maximum at 590 nm) is therefore an effect of the strong intermolecular stacking interactions characterizing this complex, as evidenced by the Independent Gradient Model based on Hirshfeld partition analysis on dimers of the three systems.

**Fig. 26:**
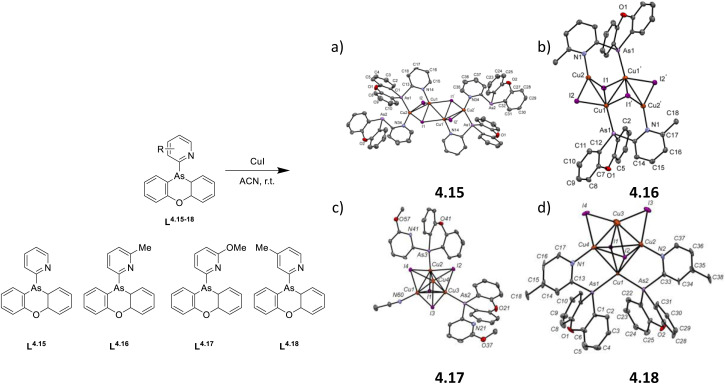
Synthesis and crystal structures of 4.15 (a), 4.16 (b), the complex obtained by slow evaporation of the solvent from a saturated solution of 4.17 in ACN (c) and 4.18 (d). Hydrogen atoms are not shown for clarity. Adapted with permission from ref. 175. Copyright 2024, The Royal Society of Chemistry.

Examples of distorted chair-like tetramers were reported in a paper from 2024,^175^ illustrating how the size and position of ligand's substituents can affect both the complex composition and Cu_4_X_4_ core structure. This work reports on a series of luminescent Cu_4_I_4_ clusters supported by a bidentate As^N-ligand, namely *R*-6-(10*H*-phenoxarsinin-10-yl)pyridine. By varying the substituent on the Py ring (Fig. 26), from H (L^4.15^) to 2-Me (L^4.16^), 2-OMe (L^4.17^) and 4-Me (L^4.18^), clusters with ligand : copper stoichiometry equal to 1 : 1 (4.15and 4.17) and 1 : 2 (4.16and 4.18), having distorted stair-step (4.15and 4.16), cubane (4.17) or octahedral ‘fox-type’ (4.18) geometries, were obtained (Fig. 26).

**Fig. 27:**
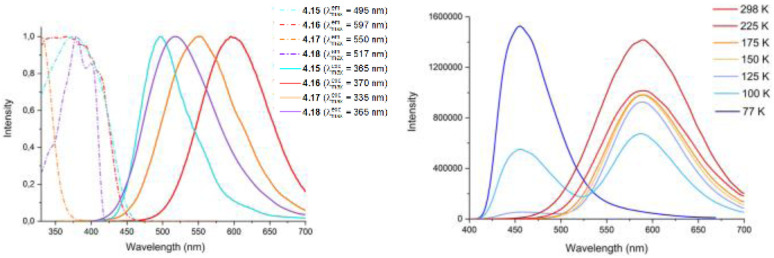
Left: solid-state excitation and emission spectra of 4.15–18 at r.t. Right: solid-state emission spectra of 4.17 in the 298–77 K temperature range. Adapted with permission from ref. 175. Copyright 2024, The Royal Society of Chemistry.

In these complexes, the As^N-ligand acts as bidentate (in 4.16 and 4.18) or monodentate, *via* As (in 4.18, because the nitrogen atom of the pyridyl fragment is fully shielded) or even as both mono- (*via* N) and bidentate (in 4.15). Solid samples of 4.15–18 show at r.t. a single emission in a broad spectral range (495–597 nm, Fig. 27a), whose assignment was established through DFT/TDDFT calculations. According to its cubane structure, the single emission of 4.17 was described as a ^3^CC one, slightly red-shifted at low temperature, in agreement with a contraction of the Cu_4_I_4_ core. Emission of 4.15 and 4.18 was assigned to a ^3^(M + X)LCT state. In particular, while 4.15 behaves as typical stair-step 0D clusters, with temperature independent emission, 4.18 represents an anomaly with respect to the thermochromic behavior normally observed for octahedral clusters (see below). In fact, its emission is only slightly shifted to blue by cooling the sample to 77 K according to a prevailing rigidochromic effect, probably due to its original ‘fox-type’ core arrangement. The other stair-step complex 4.16, whose Cu_4_I_4_ core structure approaches that of octahedral tetramers, displays a strong thermochromism with appearance of a HE emission when lowering the temperature and complete quenching of the LE one at 77 K. Unlike other octahedral complexes, however, the dual emission was attributed to two ^3^(M + X)LCT states (a symmetrical one at HE and its less symmetrical counterpart at LE) deriving from a structural distortion centered on one ligand (Fig. 27b).

**Scheme 38:**
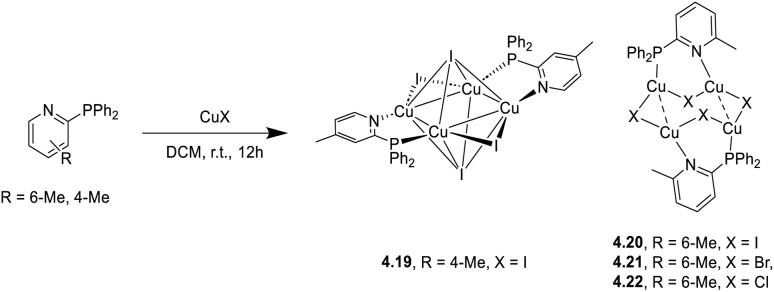
Synthesis of 4.19–22.

Typical thermochromic behavior was reported for other Cu_4_X_4_ clusters of octahedral shape. A deep investigation in this direction, including a systematic analysis of the dependence of the luminescent properties on the bridging halides, was conducted on four [Cu_4_X_4_L_2_] (X = I, Br or Cl) compounds bearing 2-(diphenylphosphino)pyridines as bridging ligands, 4.19–22 (Scheme 38).^161^

**Fig. 28:**
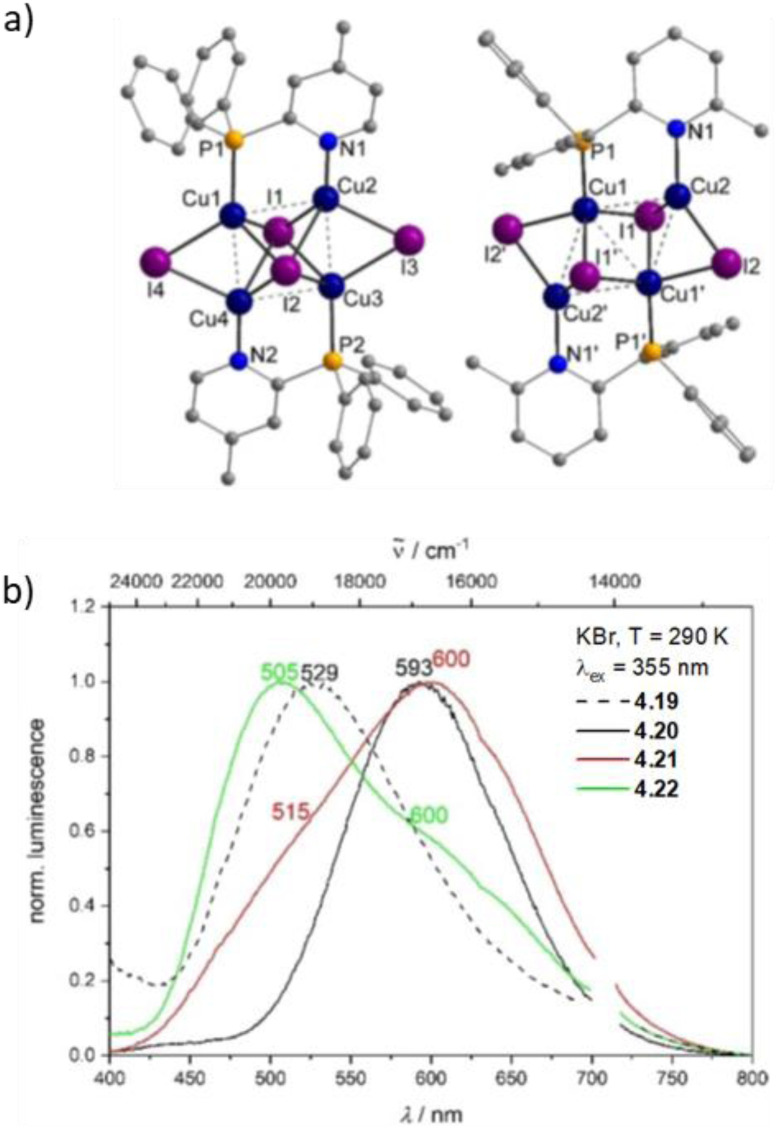
(a) X-ray structures of 4.19 (left) and 4.20 (right); (b) r.t. solid state emission spectra (KBr) of 4.19–22 recorded at 290 K with *λ*_exc_ = 355 nm. Adapted with permission from ref. 161 under the terms of the Creative Commons license. Copyright 2021, John Wiley and Sons.

SCXRD studies on 4.19 and the isostructural 4.20–22 (Fig. 28a) showed that these compounds possess a *C*_i_ symmetry (only approximate for 4.19 and 4.22), with a Cu_4_ parallelogram much more stretched in 4.20–22 with respect to 4.19, a difference imputable to crystal packing effects. Moreover, the Cu⋯Cu distances in 4.19 (2.54–2.76 Å) are slightly shorter than those of 4.20 (2.67–2.78 Å), which in turn are shorter than its chloride analogue (2.78–2.92 Å).

**Fig. 29:**
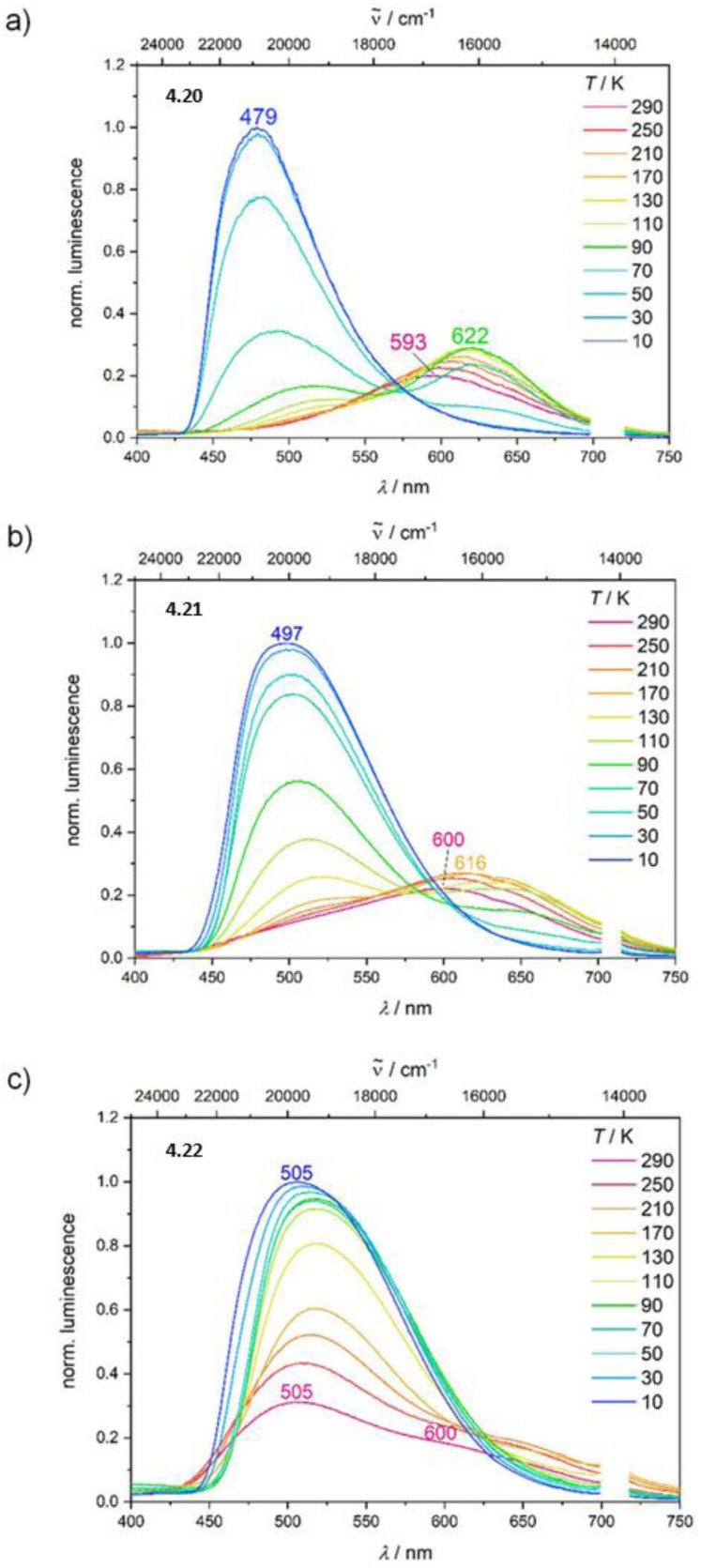
(a) Emission spectra (KBr) of (a) 4.20, (b) 4.21 and (c) 4.22 in the 10–290 K temperature range with *λ*_ex_ = 355 nm. Adapted with permission from ref. 161 under the terms of the Creative Commons license. Copyright 2021, John Wiley and Sons.

4.19 displays, in the KBr matrix, a broad unstructured emission at 529 nm characterized by a slight rigidochromism (blue shift of 6 nm) and a doubling of intensity upon cooling to 10 K (Fig. 28b). In contrast, 4.20–22 show a remarkable and reversible temperature-dependent phosphorescence (Fig. 28b and 29). In particular, 4.20 displays single emission (593 nm) at r.t., which redshifts to 621 nm with a slight increase in intensity when the sample is cooled to 90 K, indicating shrinkage of the Cu_4_I_4_ core. Upon further cooling, this emission disappears, overwhelmed by a blue emission which reaches a maximum intensity at 10 K (479 nm). Quite similar behavior is shown by 4.21, though the blue emission is already visible at r.t., while for 4.22 the blue emission dominates the spectrum already at r.t. Measurements performed on neat films, EtOH solutions and powders resulted in very similar spectra, denoting scarce influence of the matrix on the emissive properties.

**Fig. 30:**
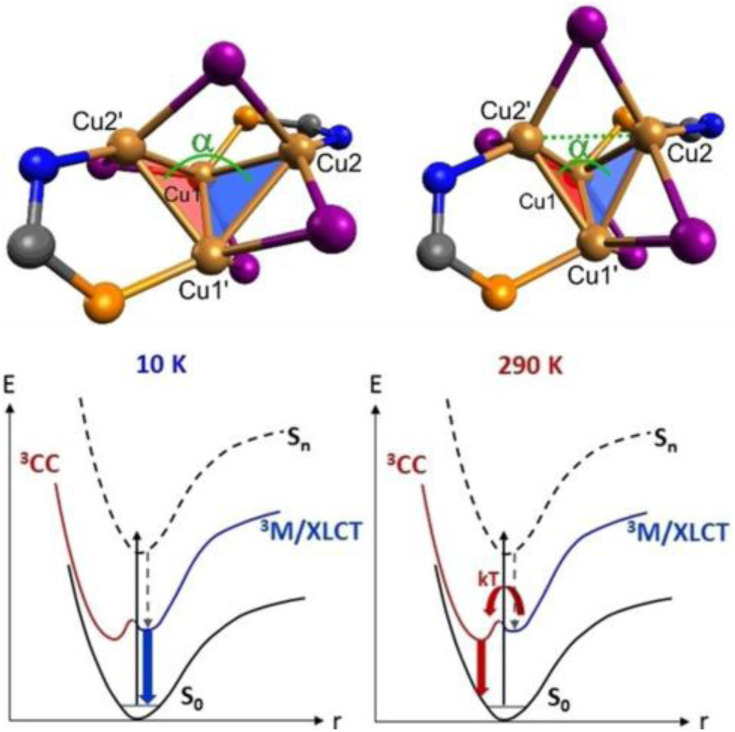
Top: fragments of the optimized T_1_ state of 4.19 in the open (left) and closed (right) butterfly conformations; bottom: qualitative energy diagram drawn for complexes 4.20–22, reporting the blue ^3^M/XLCT emission at 10 K (left) and the red ^3^CC emission at 290 K. Adapted with permission from ref. 161 under the terms of the Creative Commons license. Copyright 2021, John Wiley and Sons.

The temperature-dependent emission properties observed for 4.20–22 underline the presence of two distinct emissive excited states separated by an energy barrier, since at 10 K only one emission (the HE one) is observed. Moreover, the magnitude of the barrier increases in the direction I < Br < Cl, in agreement with the trapping of the chlorine derivative in the HE state. Intriguingly, these emissive species do not correspond to different triplet states, as generally proposed, but to two different minima in the T_1_ potential energy surface. According to unrestricted DFT calculations, in fact, compounds 4.20–22 assume in the T_1_ state a butterfly shape in either an open (similar to the X-ray *C*_i_ structure) or a close form (Fig. 30 top left), whose energies agree, respectively, with the HE and LE emissive states. The two minima correspond to different characters of the associated transitions, *i.e.*^3^(M + X)LCT (open form) and ^3^CC (close form), as schematically depicted in Fig. 30 bottom. The similarity between the T_1_ open geometry and the ground state one explains the kinetic trapping at low temperature. Moreover, the computed energy profile connecting the open and closed butterfly structures provides barrier heights correlating well with the observed relative emission intensities. Interestingly, the proposed mechanism was further supported by time-resolved step-scan FTIR measurements performed at 20 and 290 K, which perfectly match the IR spectra computed, respectively, for the open and close butterfly conformations. As an additional proof of the consistency of the model, calculations on 4.19 provided only one minimum in the T_1_ surface, corresponding to the close butterfly-shaped conformation, *i.e.* the ^3^CC state, in agreement with the observed temperature independence of the emission spectrum and the almost unvaried FTIR spectra at 20 and 290 K.

**Fig. 31:**
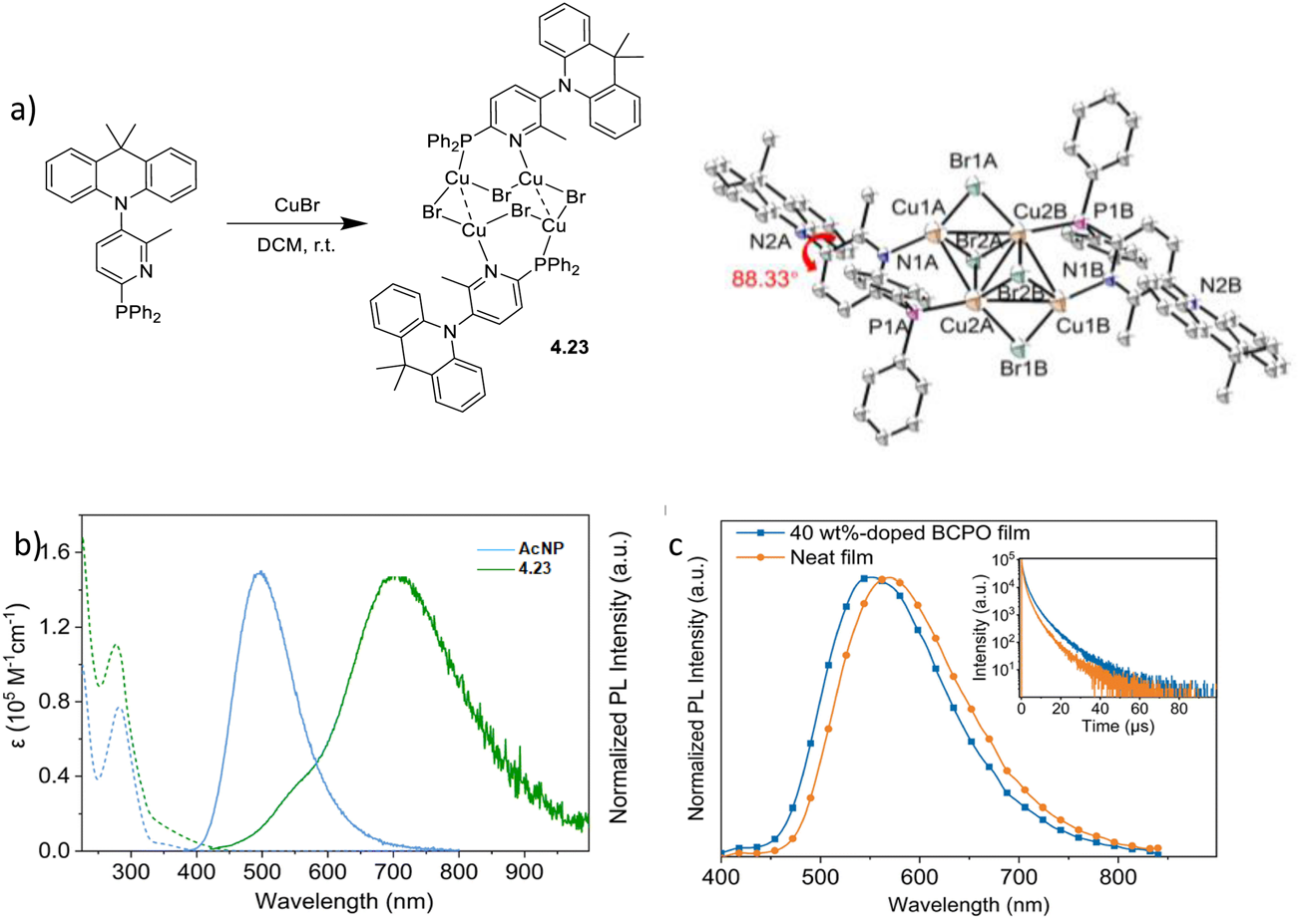
(a) Synthesis and crystal structure of 4.23; (b) absorption (dotted line) and emission (solid line) spectra of AcNP and 4.23 in DCM (2 × 10^−5^ M); (c) PL spectra and decay curves (inset) of 4.23. Adapted with permission from ref. 206 Copyright 2024, The Royal Society of Chemistry.

Another example of distorted octahedral Cu_4_X_4_ tetramer was reported in 2024 by Lu *et al.*^206^ [Cu_4_Br_4_(N^P)_2_] (4.23, Fig. 31a) (where N^P = 10-(6-(diphenylphosphaneyl)-2-methylpyridin-3-yl)-9,9-dimethyl-9,10-dihydroacridine, AcNP, is a D–A bidentate ligand) exhibits a *C*_i_ symmetric structure (Fig. 31b) very similar to that of 4.20–22 series, with the four coordinated copper atoms forming a very stretched parallelogram and Cu⋯Cu distances (2.81–2.91 Å) almost overlapping those of 4.22. The emission spectrum of 4.23 in DCM at r.t. (Fig. 31b), with a weak and broad band at about 700 nm and a shoulder at about 550 nm, resembles that of 4.21 in EtOH, though shifted at much lower energy owing to the D–A nature of the ligand. Despite these similarities, the authors, based on DFT/TDDFT calculations, assigned the LE emission to a mixed ^3^(M + X)LCT plus ILCT transition, rather than a ^3^CC one, as proposed for 4.21. In both doped (in bis-(4-(*N*-carbazolyl)phenyl)-phenylphosphine oxide, BCPO, host) and evaporated neat films, the emission maximum blue-shifts to 552 and 567 nm, respectively, and emission becomes much more efficient (*Φ* = 47 and 38%, respectively, Fig. 31c). Such results were explained considering, on the one hand, the partially suppressed excited-state distortion in a rigid environment and, on the other hand, the highly twisted structure of the D–A ligand (the dihedral angle between the acridine unit and the Py ring measures 88.33°). This conformation, in fact, effectively separates the emission centers of adjacent molecules preventing short-distance Dexter energy transfer, which is primarily responsible for concentration quenching effects. Analysis of the temperature dependence of emission spectra and decay times of 4.23 in both doped and neat films, with a slight red-shifted emission spectra and considerably prolonged lifetimes going from 300 to 77 K, suggested a TADF mechanism at r.t., a hypothesis supported by the CT character of the S_1_ and T_1_ excited states, ensuring a small S_1_–T_1_ splitting (0.15 from calculations and 0.055 eV from measurements on the doped film, respectively). Such appealing properties, together with the high stability of the compound, decomposing at 365 °C, prompted its use in vacuum deposition OLEDs, demonstrating high EQEs (12.8% and 10.2% for 40 wt% doped and non-doped devices, respectively).

**Fig. 32:**
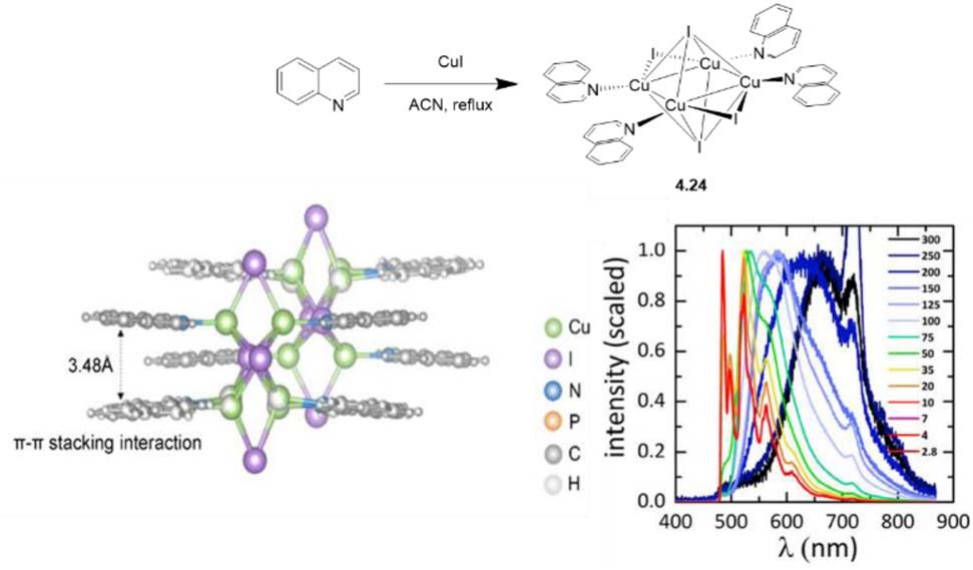
Top: synthesis; bottom: crystal structure (left) and temperature-dependent photoluminescence (PL) spectra recorded between 2.8 and 300 K under 360 nm excitation (right) of 4.24. Adapted with permission from ref. 173. Copyright 2022, American Chemical Society.

Single and strongly thermochromic emission was reported for the octahedral tetramer [Cu_4_I_4_L_4_] with qn as the organic ligand, 4.24, characterized by very close (2.158 Å) Cu⋯Cu contact and a short centroid–centroid distance between aromatic rings (Fig. 32).^173^ The large Stokes shift (1.36 eV) and FWHM (151 nm) at r.t., together with the blue shift and the appearance of vibronic components with decreasing temperature, indicate a combination of ^3^CC and ^3^(M + X)LCT origin, with the first contribution dominating at r.t. and the second one at low temperature (Fig. 32).

**Fig. 33:**
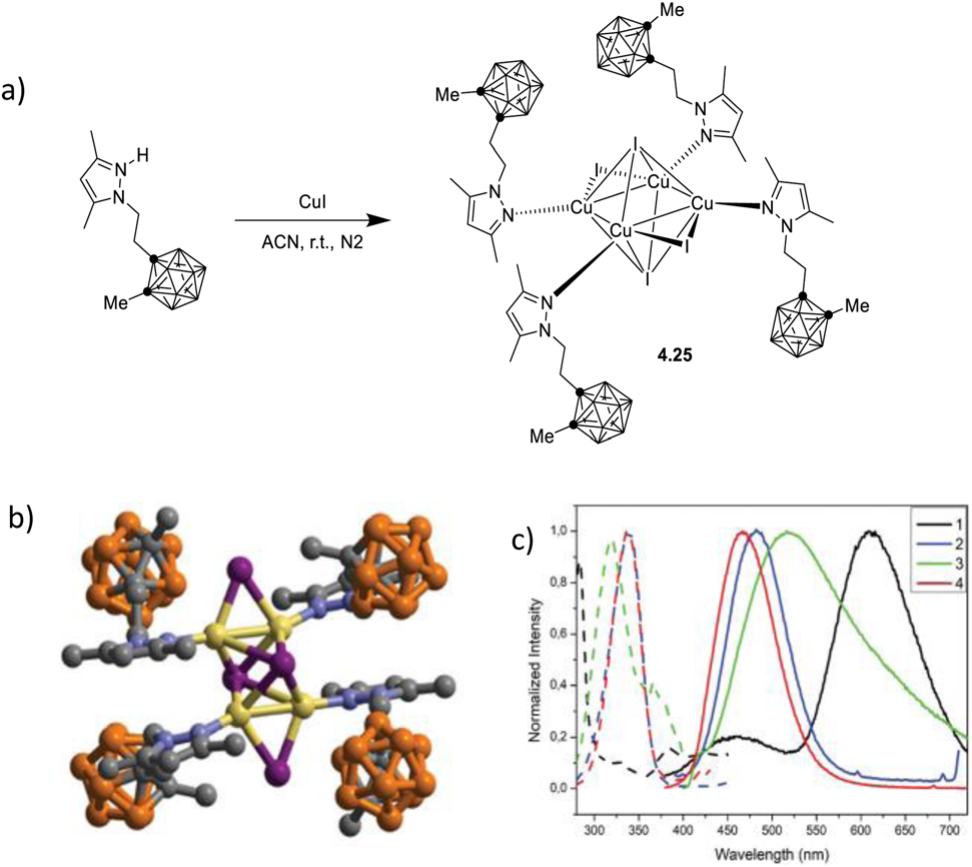
Synthesis (a) and crystal structure, with H atoms omitted for clarity (b) and excitation (dashed lines) and emission (solid lines) spectra (c) for compound 4.25 (green curves) in the solid state at 298 K. Adapted with permission from ref. 172 under the terms of the Creative Commons license. Copyright 2021, The Royal Society of Chemistry.

An additional [Cu_4_I_4_L_4_] octahedral cluster, 4.25, based on monodentate ligands was obtained using an N-substituted carboranyl pyrazole derivative (Fig. 33a).^172^ Interestingly, the corresponding ligand without the methyl group in the *ortho* position of the carboranyl group gave a dinuclear Cu_2_I_2_ complex (see Section 3).^172^ In the crystal structure of 4.25, the Cu atoms are perfectly coplanar, forming an almost rectangular, quite elongated array (Cu⋯Cu distances of 2.592 and 2.929 Å). The crystalline solid shows, at r.t., a single very broad emission with the maximum at 517 nm, *i.e.* about halfway the HE and LE bands reported for dual emitting octahedral clusters (Fig. 33c). Quite unexpectedly, the corresponding *Φ*, 4.9%, was much lower than those reported for octahedral tetramers, despite the presence of the spherical-shaped carboranyl group, able to prevent the formation of intermolecular π–π stacking interactions reducing possible concentration quenching effects.^207^ According to DFT/TDDFT calculations, 4.25 appears to follow the mechanism depicted in Fig. 30, bottom right. The LE absorption bands, in fact, display a (M + X)LCT character, but UDFT optimization of T_1_ leads to a strongly distorted geometry, poorly reminiscent of the S_0_ one, with associated deexcitation transitions of ^3^CC character, denoting that the system should overcome an energy barrier. However, no photoluminescence measurements were performed at low temperature, aimed at ascertaining a blue-shift of the emission band (Fig. 30, bottom left), in agreement with the mechanism proposed by Boden *et al.*^161^

**Scheme 39:**
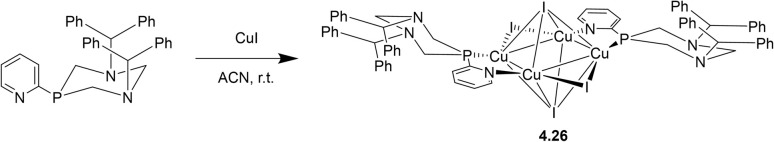
Synthesis of 4.26.

An octahedral [Cu_4_I_4_(P^N)_2_] complex was obtained by Strelnik *et al.*^169^ using 1,3-diaza-5-phosphacycloxehane as the ligand with limited conformational flexibility. 4.26 (Scheme 39) crystallizes in different solvate forms according to the crystallization solvent or the vapor exposure. Essentially, two conformers are individuated, a “compact”, 4.26c, or a “stretched”, 4.26s, one, characterized by Cu_4_ parallelograms slightly or strongly stretched, respectively. Intriguingly, crystalline powders of 4.26c, devoid of cocrystallized solvent, display at r.t. intense green phosphorescence centred at 492 nm, while those of 4.26s emit in the red region at 680 nm and display thermochromism with the appearance, upon cooling the sample down to 83 K, of an additional HE band at 490 nm. Based on DFT/TDDFT calculations, well reproducing the two ground state conformations and the associated emissions, both green and red emissions of 4.26 were assigned to excited states of ^3^(M + X)LCT character, with a silent ^3^CC state. The observed vapochromism of this compound, described in more detail in the dedicated section (see Section 6.3), makes it suitable as a sensor for volatile organic compounds (VOCs).

### 5.2 Halogen free Cu_4_ clusters

The variety of emissive triplet states accessible to tetranuclear Cu_4_X_4_ clusters and their possible dependence on temperature and external stimuli may represent a potential drawback in view of generating single-mode emissions with specific energy. The few reports on halogen free photoluminescent tetrameric clusters that appeared in the literature were mainly based on pyrazolate derivatives. Cu_4_Pz_4_ compounds display at r.t. a single, bright emission whose energy strongly depends on the substituents on the pyrazole ring. Importantly, while the planarity of the trinuclear Cu_3_Pz_3_ structures often leads to intermolecular stacking which strongly affects their emissive states, the saddle shape of Cu_4_Pz_4_ complexes prevents any significant intermolecular interaction, and the associated photophysical behavior is therefore unaffected by neighbouring molecules.^195^ Moreover, comparison between tri- and tetranuclear pyrazolate complexes bearing analogous alkyl substituents in 3,5 positions of pyrazole indicates that emissions are considerably stronger for the latter, in agreement with the shorter intramolecular Cu⋯Cu distances in the tetranuclear complexes compared with the intermolecular ones in the trinuclear complexes.^182,195^

**Scheme 40:**
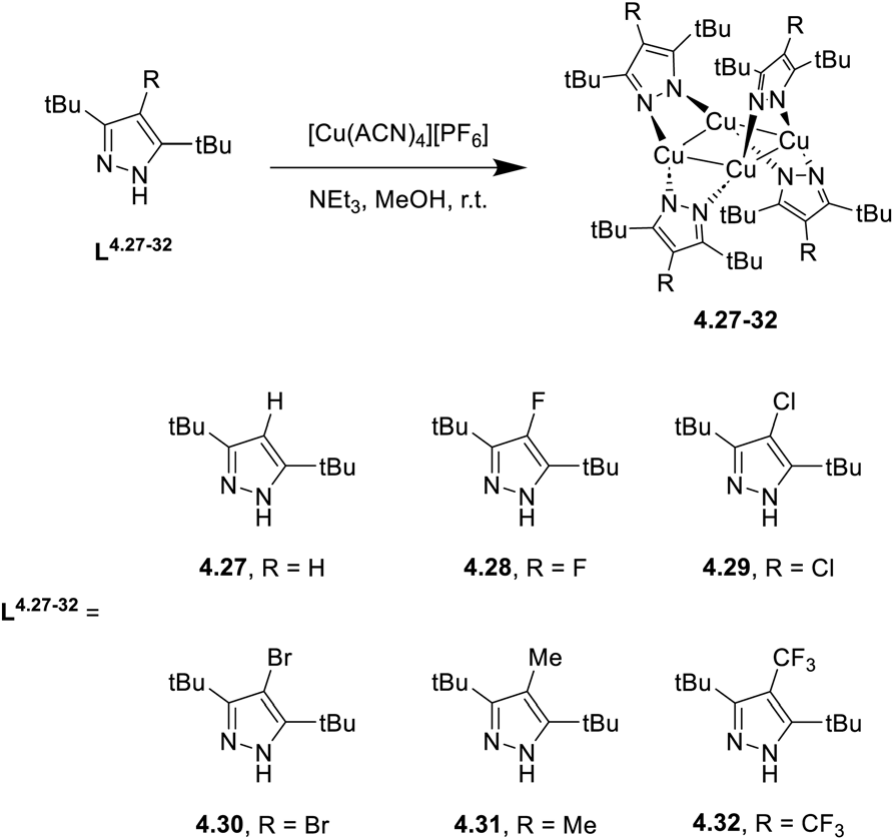
Synthesis of 4.27–32.

A comprehensive investigation on Cu_4_Pz_4_ complexes was reported by Wei and coworkers,^208^ focusing on a series of derivatives with fixed 3,5-di-^*t*^Bu-pyrazole substitution and variable groups (R = H, F, Cl, Br, and CH_3_, 4.27–31) in the C4 position (Scheme 40).

**Fig. 34:**
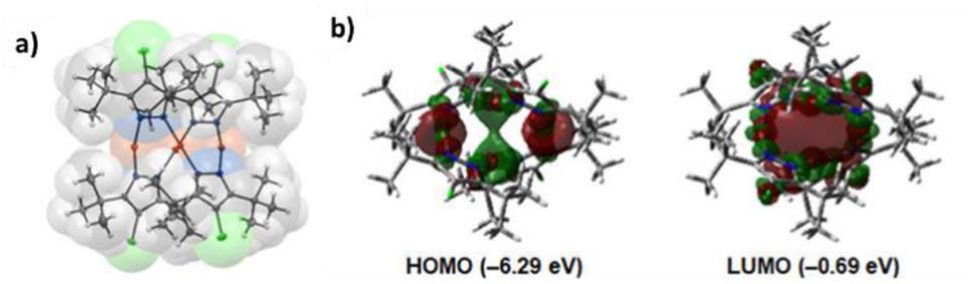
(a) ORTEP representation of the X-ray crystal structure (thermal ellipsoids are drawn at 50% probability level) and (b) HOMO and LUMO plots of complex 4.29. Adapted with permission from ref. 208. Copyright 2022, American Chemical Society.

According to SCXRD analysis and computational studies, these complexes are characterized by relatively short (about 2.9 Å) distances between adjacent Cu atoms and, consequently, well-defined CC nature for both the HOMO and LUMO (Fig. 34b), with the only exception of the bromine derivative whose LUMO displays significant contributions from the halogen.

**Fig. 35:**
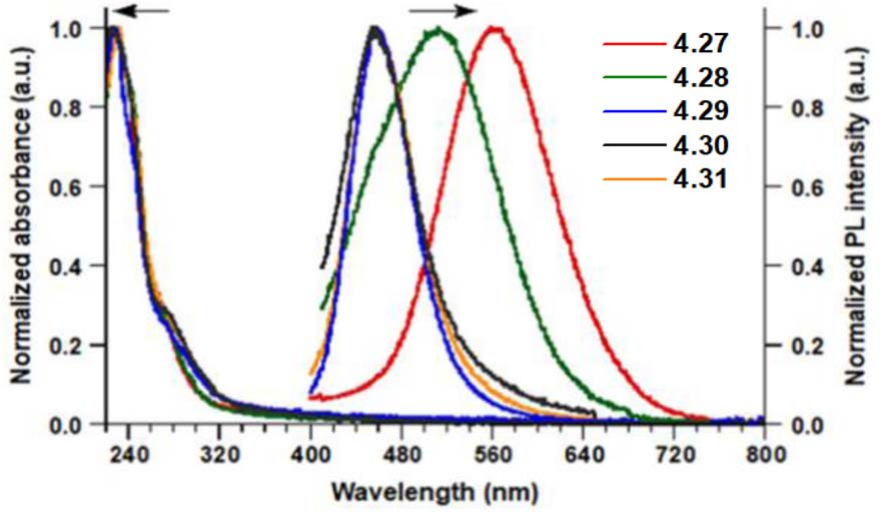
Absorption and solid-state photoluminescence spectra of complexes 4.27–31. Adapted with permission from ref. 208. Copyright 2022, American Chemical Society.

Photoluminescence studies on thin films of 4.27–31 at r.t. revealed in almost all cases very intense phosphorescence (Fig. 35), ascribed to radiative decay from a ^3^CC state, with *Φ* approaching unity for 4.29 and 4.31. Only for 4.30, a much lower (8%) *Φ* was observed, probably due to a competitive ^3^MLCT process, as supported by DFT calculations (see above), which increases the *K*_nr_. Essentially, identical results were obtained from microcrystalline powders, further demonstrating the molecular nature of the solid-state photoluminescence of Cu_4_Pz_4_ complexes. Remarkably, the emission wavelengths shift from yellow (4.27) to green (4.28) and then to deep blue (4.29–31), demonstrating a strong correlation with the van der Waals volumes, *V*_vdW_, of the substituents in the C4 position (Fig. 35b). Such dependence was explained, through DFT calculations, considering the greater rigidity of 4.29–31, having R groups with a larger *V*_vdW_ with respect to 4.27 and 4.28. In fact, the R substituent can block the 3,5-^*t*^Bu groups in the former compounds but not in the latter, where they are free to rotate in solution. Their SCXRD structure reflects the different hindrance of R, showing not only different conformations of the 3,5-^*t*^Bu groups (bisecting in 4.29–31 and eclipsed in 4.27 and 4.28) but also the shape of the Cu_4_ rhombus, which is more stretched for 4.29–31. As computationally demonstrated, both factors grant greater compactness and rigidity to these structures, preventing their reorganization in the excited state and therefore resulting in the rather unexpected (owing to its ^3^CC nature) deep-blue phosphorescence. This hypothesis is further supported by the results previously reported for the analogous Cu_4_Pz_4_ derivative with 3,5-^i^Pr, rather than 3,5-^*t*^Bu groups,^195^ displaying at r.t. an intense emission at much lower energy (550 nm) compared to 4.30 (457 nm).^195^ While the mechanism underlying the two processes, *i.e.* radiative decay through a ^3^CC state, is the same for the two compounds, the smaller ^i^Pr substituents in C3 and C5, compared to ^*t*^Bu, reduce the rigidity of the structure resulting in the observed red shifted emission.

Shortly after, an additional derivative (R = CF_3_, 4.32, Scheme 40) was reported by the same research group,^209^ aimed at exploring the photophysical behavior of a Cu_4_Pz_4_ complex with a group in the C4 position even larger than CH_3_. Against the prediction of a further enforcement of the conformational rigidity shown by 4.29–31 and the associated deep-blue emission, both powder and thin film samples of 4.32 display at 295 K a main emission centered at 519 nm, besides a shoulder at higher energy. Both emissions correspond to the same absorption band having, according to DFT calculations, ^3^CC character as found for 4.27–31. The behavior of 4.32 was explained by analysis of its crystal structure, evidencing several conformers in the unit cell, all characterized by severe distortion involving both the ^*t*^Bu groups and the Cu_4_ core, which strongly deviates from planarity. According to DFT/TDDFT calculations, the presence of multiple structures results in both rigidochromic and red-shifted emissions, the latter induced by excited-state Cu_4_ distortion. This study therefore underlines the importance of a careful choice of substituents on the pyrazolate ring to support conformations that minimize structural reorganization in the excited state.

**Scheme 41:**
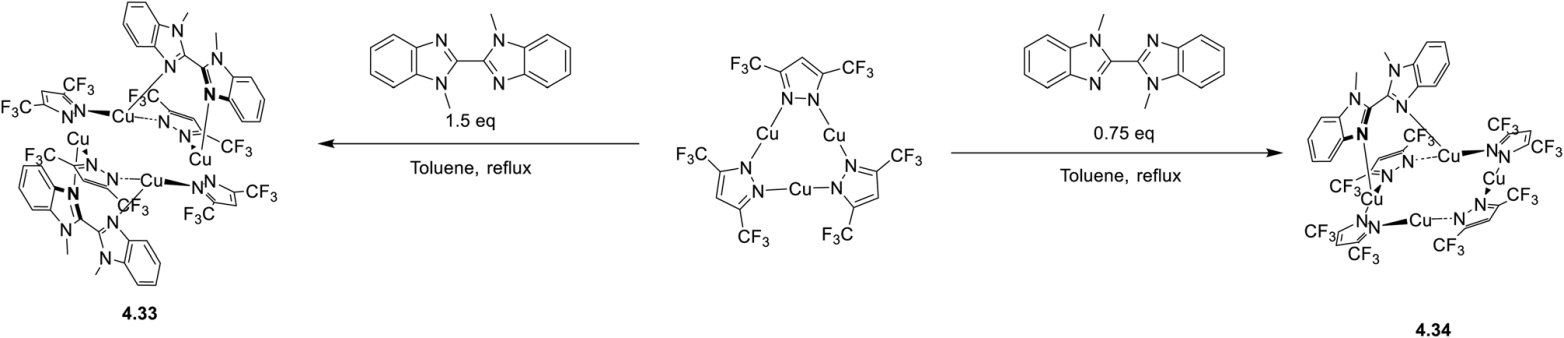
Synthesis and chemical structures of complexes 4.33 and 4.34.

In 2023, Yakovlev *et al.* obtained two different tetranuclear heteroleptic pyrazolates by treating the trinuclear 3,5-bis(trifluoromethyl)pyrazolate Cu(i) complex with 1,10-dimethyl-2,2′-bibenzimidazole, in excess or in defect with respect to the macrocycle (Scheme 41).^210^ In the former case, the tetranuclear complex contains two molecules of bridging ligand (4.33), whereas in the latter it contains only one ligand (4.34).^210^ The coordination of dibenzimidazole to the pyrazolate complex leads to the elongation of the Cu⋯Cu distances (about 3.3 Å) with respect to Cu_4_Pz_4_ complexes. These features, together with the high fluorescence efficiency of dibenzimidazole, result in the dual-emissive behavior of the two complexes in the solid state at r.t., with a HE fast LC contribution and a LE phosphorescent tail of ^3^MLCT nature at about 520 nm. At 77 K, such long-lived component is greatly intensified only for 4.34, while it is weakened in 4.33, in agreement with the higher ligand to macrocycle ratio in the latter.

## 6. Stimuli responsiveness

### 6.1 Thermochromism

Luminescent thermochromism refers to temperature-induced changes in the photoluminescent behavior including emission maxima, intensity and lifetime. Temperature can affect photoluminescence through a plethora of different mechanisms spanning from molecular dynamics^211^ to structural rearrangement^212^ and loss of coordinated solvent molecules.^68^ Thermo-responsive light emitting materials have attracted great attention for their potential applications in various fields comprising temperature sensing, bioimaging, optoelectronics, and information processing.^213^

Recent examples of Cu(i) thermochromic materials of different nuclearities have been reported and discussed in previous sections. It is however useful to recall here that Cu(i) complexes are particularly suitable as luminescent thermometers, thanks to their ability to exhibit dual band emission with relative intensity varying with temperature.^214,215^ This feature enables ratiometric temperature sensing, making copper complexes highly effective for precise thermal monitoring applications.^216^

As reported in Section 5.1, tetrameric cubane-like Cu_4_I_4_ clusters, especially those containing tertiary phosphines and/or N-donating ligands,^217,218^ often showing dual emission of MLCT and CC origins, are traditional Cu(i) thermochromic materials. In this regard, octahedral Cu_4_I_4_ analogues and trinuclear derivatives have been less investigated even though their ability to display dual band emission has already been established (see, for example, ref. 138 and 161).

### 6.2 Mechanochromism

Mechanochromic luminescence, MCL, is related to changes in the photophysical properties of a material in response to an external stimulus such as grinding, shearing, rubbing or high pressure. Compounds exhibiting reversible MCL have attracted great attention from the scientific community due to their high sensitivity with practical applicability in different fields spanning from data storage to strain detection and anticounterfeiting.^219–222^ Compared with π-conjugated organic molecules, the development of transition-metal complexes with MCL properties is less advanced and mainly based on Pt(II) and Au(i) systems, although a few examples with Zn(ii), Al(iii), Ir(iii) and Ag(i) derivatives have also been reported.^223^ Concerning Cu(i) complexes, the first reports date back to 2010, when the MCL of a tetranuclear cubane-type cluster^224^ and trimeric Cu_3_pz_3_ pyrazolate complexes^225^ was investigated. Since then, various examples of trimeric and tetranuclear Cu(i) complexes or coordination polymers have been reported, in which either the alteration of Cu⋯Cu distances and/or π–π interactions or crystal-to-amorphous conversions have been envisaged as the main origin of their response to the mechanical stimulus.^226,227^

Even more rare are MCL dinuclear and mononuclear complexes, the latter appeared in the literature only in 2020. While for dinuclear derivatives, changes in the Cu⋯Cu distances play a crucial role in the modification of the emitting states, for mononuclear complexes, the MCL response is usually related to the disruption of weak intra- and intermolecular interactions (see below).

#### 6.2.1 Advancements during 2020–mid-2025

**Scheme 42:**
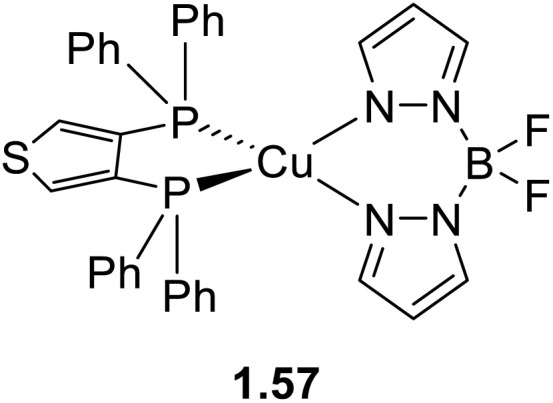
Chemical structure of 1.57.

A mononuclear Cu(i) complex (1.57, Scheme 42) displaying reversible MCL was prepared by the synergic combination of a flexible and a rigid ligand, bis(pyrazol-1-yl)borohydrate (Pz_2_BH_2_) and 3,4-bis(diphenylphosphino)thiophene (3,4-dppTp), respectively.^228^ Such design was aimed at facilitating the formation of a loose structure beneficial to MLC while maintaining the emissive properties of the compound by suppression of non-radiative decays. 1.57 was isolated as two polymorphs, characterized by distinctive blue and yellow emissive features at 472 nm (*τ* = 22 μs and *Φ* = 59%) and 528 nm (*τ* = 19 μs and *Φ* = 78%), respectively, both assigned to TADF. A crystallographic investigation disclosed in both compounds the presence of strong intermolecular interactions and co-crystallized DCM molecules, and, only for the blue polymorph, of a porous structure accommodating the solvent molecules linked through strong C–H⋯Cl bonds. Interestingly, mechanical grinding of the blue polymorph produced an 80 nm red-shift resulting in a yellow emission. The blue luminescence is completely restored by fuming the yellow powder with DCM or diethyl ether vapors, a conversion cycle that can be performed only three times. Otherwise, restoration of the original emission by soaking in diethyl ether can be accomplished for up to ten cycles without degradation. A detailed photophysical investigation of the pristine and ground-fumed/soaked phases together with IR and crystallographic analysis of the samples disclosed that the MCL behavior is related to crystal-to-amorphous phase transition upon grinding. Application of external forces modifies the flexible and porous structure of the crystal, characterized by C–H⋯π intermolecular interactions and C–H⋯Cl bonds with DCM, resulting in a less rigid amorphous environment. Accordingly, quenching of the emission (*Φ* equal to 59% and 34% before and after grinding, respectively, see Table S7) was observed and associated with an increased number of non-radiative transitions in the ground sample.

**Scheme 43:**
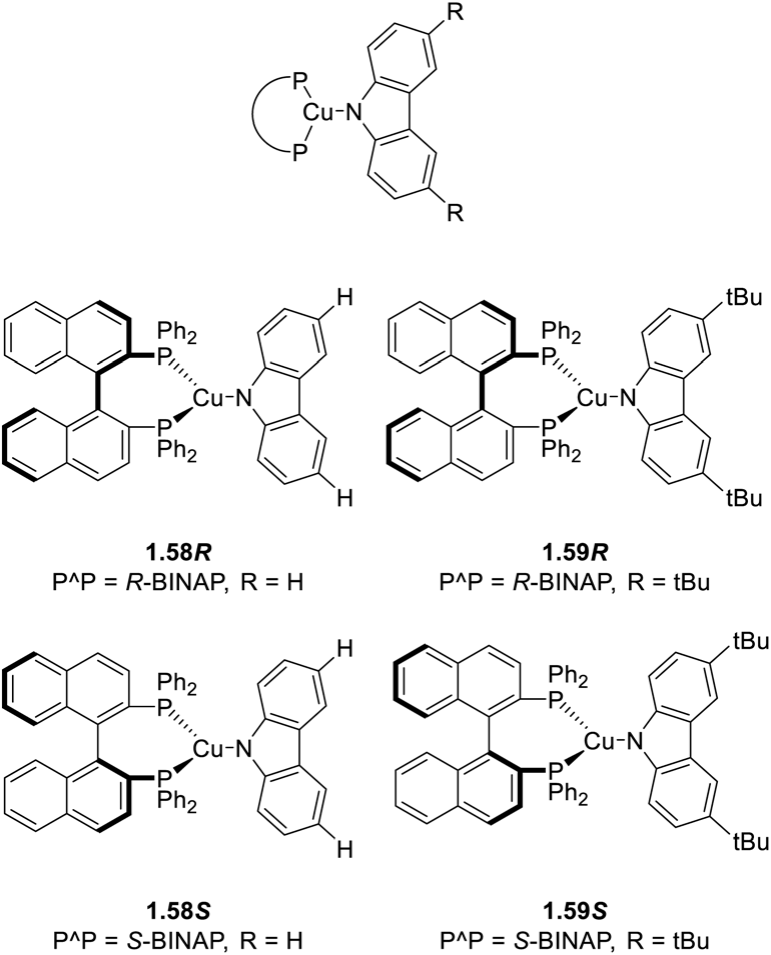
Chemical structures of 1.58*R*/*S* and 1.59*R*/*S*.

In view of optimizing CPL behavior (concomitantly high *K*_r_ and *g*_lum_ values) for device application, Muthig *et al.* investigated TADF emissive mononuclear Cu(i) complexes featuring chiral ligands.^53,229^ In particular, the family of enantiomerically pure trigonally coordinated copper(i) carbazolate (donor ligand) complexes bearing (*S*/*R*)-BINAP (BINAP: 2,2′-bis(diphenylphosphino)-1,1′-binaphthyl) as chiral acceptor ligands was investigated as CP-TADF emitters (Scheme 43).^229^

Except for their chiroptical properties, {Cu(Cbz^R^)[(*S*/*R*)-BINAP]} [R = H (1.58*R*/*S*), 3,6-^*t*^Bu (1.59*R*/*S*)] enantiomers are characterized by identical emissive features. In THF, 1.58*R*/*S* and 1.59*R*/*S* display a broad emission at 585 nm (*τ* = 190 ns and *Φ* = 1.3%) and 614 nm (*τ* = 170 ns and *Φ* = 17%), respectively, which, in agreement with variable temperature experiments, was assigned to TADF from thermally equilibrated ^1/3^LLCT states. At r.t., crystals of 1.58*R*/*S* and 1.59*R*/*S* display a broad phosphorescence from ^3^LLCT (Cz) excited states at 564 nm (*τ* = 8.7 μs and *Φ* = 22%) and 549 nm (*τ* = 30.5 μs and *Φ* = 25%), respectively. Upon grinding, a red-shift was observed with a parallel increase of *Φ* for both compounds (579 nm, *τ* = 2.5 μs, and *Φ* = 55%; 606 nm, *τ* = 1.7 μs, and *Φ* = 53%, for 1.58*R*/*S* and 1.59*R*/*S*, respectively). Such MCL was ascribed to the partial disruption of the intermolecular C–H⋯π interactions between the BINAP ligand and the PPh_2_ and Cz moieties. By grinding the crystalline material, a significant reduction of the energy splitting between the ^1/3^LLCT states and a larger energetic separation from the ^3^LC states were produced, resulting in efficient TADF. The combination of CPL and triplet/TADF emissions of these Cu(i) complexes was further exploited for the realization of a proof-of-concept CP-OLED.

A relevant bathochromic shift upon grinding was displayed by mononuclear carbene complexes 1.12a–f and 1.13a, as reported by the same research group and previously discussed in Section 2.1.1.^53^ The phenomenon was ascribed to a partial disruption of the crystalline order under mechanical stimulus, resulting in an increase of the surface area leading to efficient TADF similar to the one observed for the isolated molecules.

**Scheme 44:**
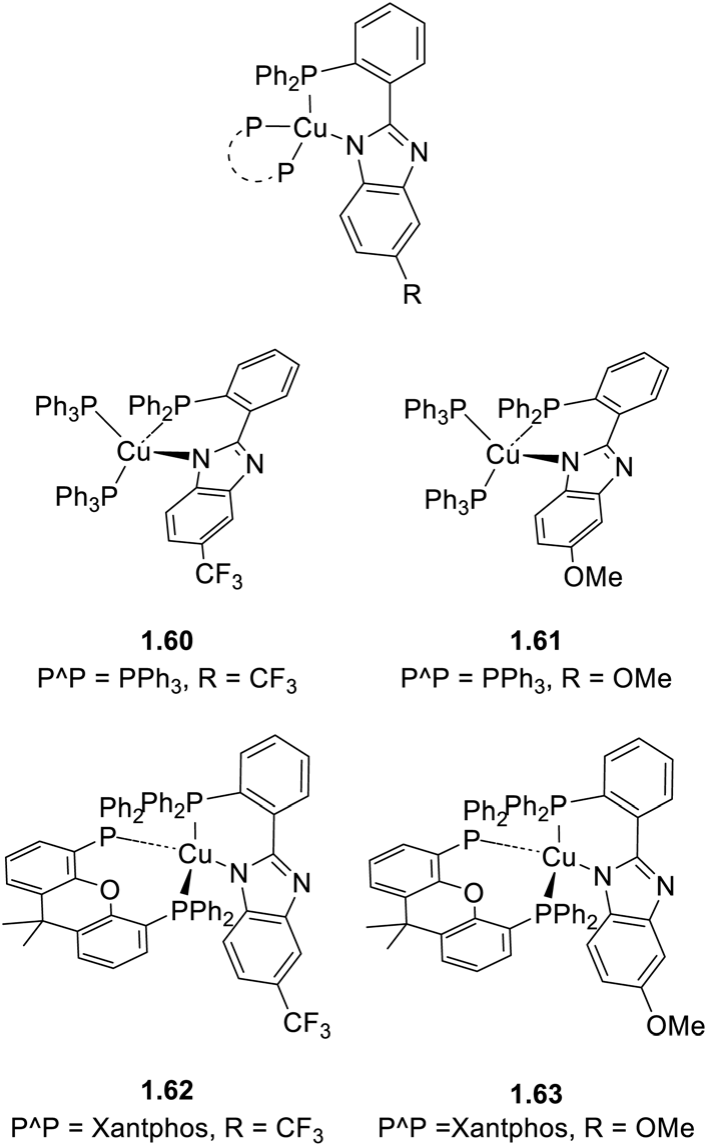
Chemical structures of 1.60–63.

Yan *et al.* reported in 2023 the MCL of four neutral [(P^P)(N^P)Cu] complexes (1.60–63, see Scheme 44) obtained by combining N^P ligands (2-(2-(diphenylphosphaneyl)phenyl)-6-R-1*H*-benzo[*d*]imidazole, where R = OMe or CF_3_) and P^P (PPh_3_ or xantphos).^230^

Complexes 1.60–63 are characterized by a broad structureless emission, respectively, at 496 (*τ* = 12.4 μs and *Φ* = 43.5%), 524 (*τ* = 6.8 μs and *Φ* = 32.6%), 503 (*τ* = 5.9 μs and *Φ* = 24.5%) and 542 nm (*τ* = 6.4 μs and *Φ* = 20.0%), assigned to TADF on the basis of theoretical calculations and variable temperature experiments. Interestingly, the higher *Φ* values of 1.60 and 1.61 suggest a positive effect of the bulkier triphenyl phosphine *versus* xantphos associated also with the formation of hydrogen bond (HB) interactions that enhance the structural rigidity. Grinding in a mortar resulted in decreased *Φ* and red-shifting of the emission to 546 (*τ* = 12.4 μs and *Φ* = 26.2%), 560 (*τ* = 6.8 μs and *Φ* = 20.6%), 534 (*τ* = 5.9 μs and *Φ* = 7.5%) and 557 nm (*τ* = 6.4 μs and *Φ* = 9.8%) for 1.60–63, respectively. XRPD studies revealed amorphization of the ground samples associated with a reduction of HB interactions and disruption of the crystal lattice. Interestingly, the original diffraction patterns and emission features can be restored by exposure to methanol vapors.

**Scheme 45:**
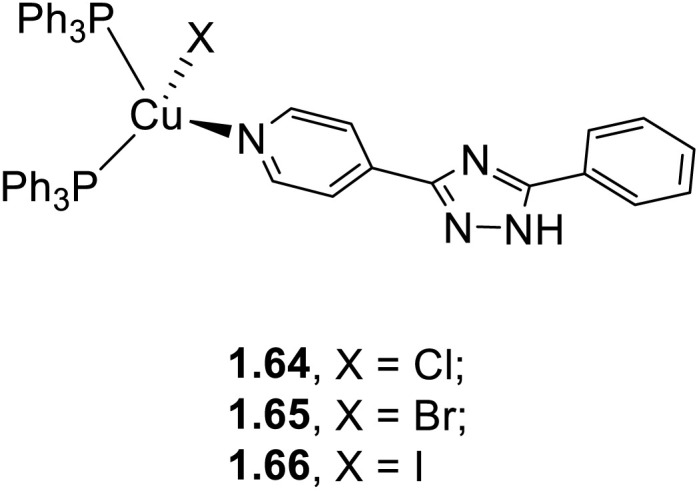
Chemical structures of 1.64–66.

In 2023, Gusev *et al.* isolated three neutral mononuclear Cu(i) halide complexes of formula [CuX(PPh_3_)_2_(L)] (where X = Cl, Br, and I; 1.64, 1.65 and 1.66, respectively, and L = 3-phenyl-5-(pyridin-4-yl)-1,2,4-triazole) (Scheme 45).^231^

In the solid state, the compounds are characterized by a broad efficient TADF (from the ^1^(M + X)LCT excited state thermally populated by a ^3^(M + X)LCT one), as indicated by variable temperature measurements, theoretical calculations and small Δ*E*_ST_ values. Moreover, their grinding in a mortar resulted in a red shift and concomitant reduction of *Φ*. In particular, the broad and unstructured green emissions of 1.64 (544 nm; *τ* = 13.2 μs and *Φ* = 58.8%), 1.65 (528 nm; *τ* = 3.72 μs and *Φ* = 70.3%) and 1.66 (522 nm; *τ* = 3.0 μs and *Φ* = 29.1%) turned orange (596 nm, *Φ* = 7%; 600 nm, *Φ* = 34%; 605 nm, *Φ* = 16%, for 1.64, 1.65 and 1.66, respectively) after grinding. The process is reversible, and the original green emission was restored by exposure to ACN vapors or gentle heating. Through a combined PXRD, DSC and IR analysis of the pristine and ground samples, this reversible MCL was related to a crystal to amorphous transition induced by disruption of intermolecular interactions, in particular HBs, upon grinding.

**Fig. 36:**
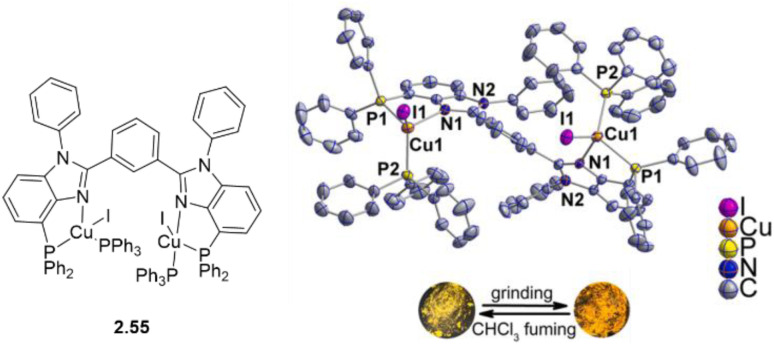
Chemical and crystal structures of 2.55. Adapted with permission from ref. 232. Copyright 2021, Elsevier.

In 2021, Lu *et al.* employed an iminephosphine tetradentate chelating ligand (P^N^N^P, having two iminephosphine N^P groups bridged through a benzene ring) for the preparation by a one pot reaction of two dinuclear Cu(i) complexes, one of which, [CuIPPh_3_]_2_(P^N^N^P) (2.55), is characterized by reversible MCL (Fig. 36).^232^

The bulky and rigid ligand inhibits non-radiative decays from the excited states, providing the compound with efficient r.t. luminescence (556 nm; *τ* = 2.32 μs and *Φ* 66.8%), which was assigned to (M + X)LCT states. A red shift (571 nm) and decreased efficiency were observed after grinding in a mortar, while the original emission was restored by exposure to chloroform vapors. PXRD analysis revealed that MCL is related to a crystalline to amorphous transition due to a reversible loss/uptake of chloroform molecules accommodated in the porous crystal structure.

**Fig. 37:**
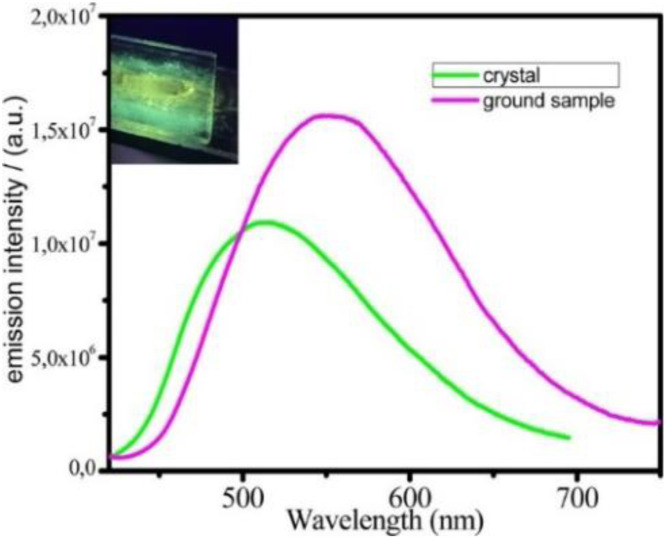
Emission spectra of unground and ground crystalline powders of 2.9. Reprinted with permission from ref. 80 under the terms of the Creative Commons license. Copyright 2023, The Royal Society of Chemistry.

Among the series of dinuclear pyridyltriazole Cu(i) derivatives reported by Gusev *et al.* and described in Section 3.1.1, a stimuli-responsive behaviour was reported for complex 2.9.^80^ In particular, the broad and greenish emission of the crystals at 507 nm red-shifted to an intense yellow band at 551 nm upon grinding in a mortar (Fig. 37). The transformation is totally reversible, and the initial emission can be restored by thermal treatment at 100 °C. Such MCL was associated with amorphization, as confirmed by XRPD, showing weaker and broader diffraction peaks after grinding. The authors suggested, by analysis of the emission lifetime and *Φ* and by analogy with other dinuclear Cu(i) complexes, that in the ground sample a partial removal of co-crystallized solvent molecules occurs, resulting in a lower energy ^3^MLTC emitting state.

**Scheme 46:**
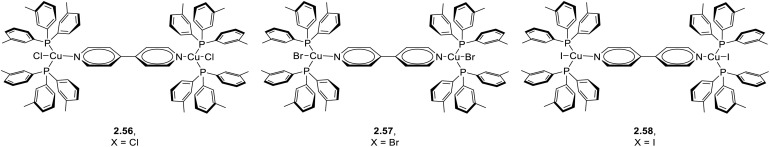
Chemical structures of 2.56–58.

Chen *et al.* reported on the MCL properties of three isostructural dinuclear Cu(i) complexes of general formula [Cu_2_X_2_(tmp)_4_(bpy)]·CH_3_CN (where X = Cl (2.56), Br (2.57), and I (2.58); tmp = tri-*m*-tolylphosphine; bpy = 4,4′-bipyridine as a bridging ligand) prepared by a one pot reaction in solution (Scheme 46).^233^

**Fig. 38:**
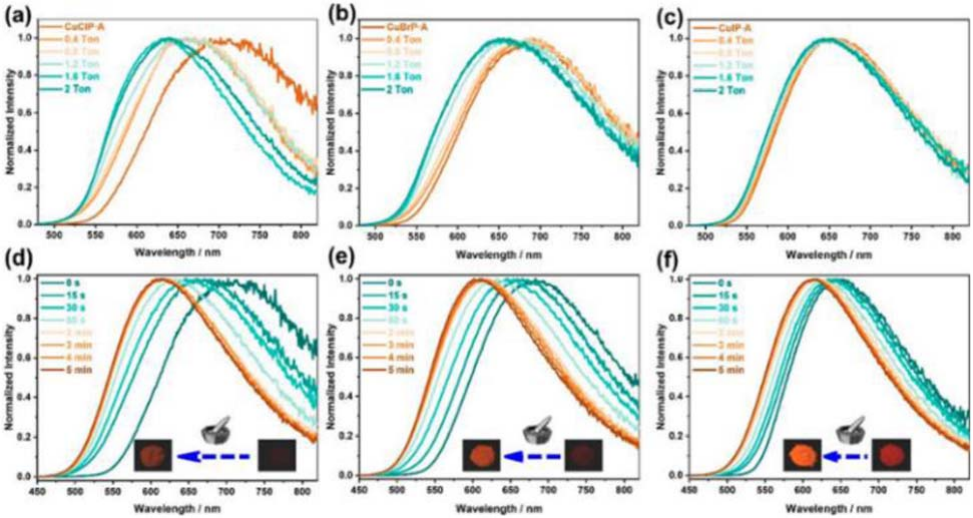
Emission spectra (*λ*_exc_ = 365 nm) of 2.56 (a), 2.57 (b), and 2.58 (c) under different pressures. Emission spectra (*λ*_exc_ = 365 nm) taken at different grinding times for 2.56 (d), 2.57 (e), and 2.58 (f). Inset: photographs of the compounds before and after fully grinding, under 365 nm UV light. Reprinted with permission from ref. 233. Copyright 2024, American Chemical Society.

Single crystal and powder X-ray diffraction, elemental and thermogravimetric analyses highlighted the presence of ACN guest molecules tightly encapsulated in the crystal structures. Solid state emission spectra of the complexes are characterized by a broad band with maxima at 702, 685 and 652 nm, for X = Cl, Br, and I, respectively (Fig. 38a–c), which were attributed to a ^3^(M + X)LCT excited state, as supported by theoretical calculations. The chlorine derivative emits close to the near-infrared region and is therefore prone to deactivation paths through non-radiative transitions, while, by increasing the halogen size, the blue shift is accompanied by intensification of luminescence. All compounds, both as crystals or powders, are characterized upon grinding by hypsochromic shift (up to 90 nm for the chlorine derivative) with emission maxima at 612 nm for all derivatives and an increase of lifetimes and *Φ*. Interestingly, this is one of the rare examples of MCL materials displaying a blue-shifted emission as a consequence of the mechanical stimulus (Fig. 38d–f). This behavior was rationalized by combined FT-IR, Raman, TGA and PXRD investigations that disclosed the release of the solvent guest molecule with concomitant crystal to amorphous transition through mechanical stimulus.

The process is fully reversible, and the original emissions can be restored by exposure to ACN vapors with good repeatability of the process up to three cycles. In addition, as suggested by theoretical analysis, the emission is affected by the solvent polarity due to the predominant role of the MLCT transition in the excited state. To this extent, a comparison between [Cu_2_I_2_(tmp)_4_(bpy)] complexes with guest solvents of different polarity confirmed that ambient polarity is crucial for MCL, observed for propanone as the guest and lacking with non-polar tetrahydrofuran.

**Scheme 47:**
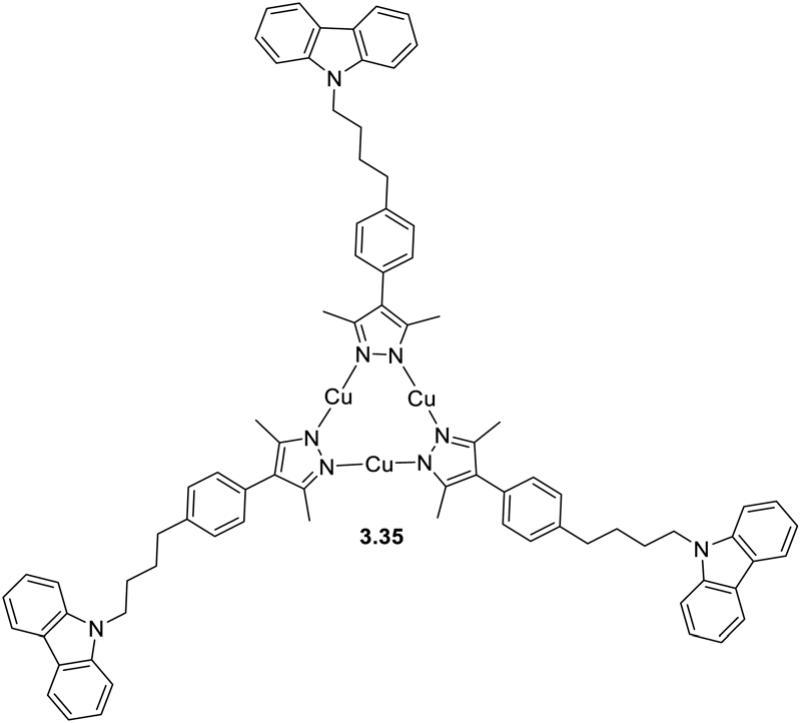
Chemical structures of 3.35.

A trimeric Cu_3_Pz_3_ derivative in which the pyrazole moiety is decorated with a Cz-functionality through a flexible *n*-butyl chain was described in 2021 by Xie *et al.* (Scheme 47).^234^

**Fig. 39:**
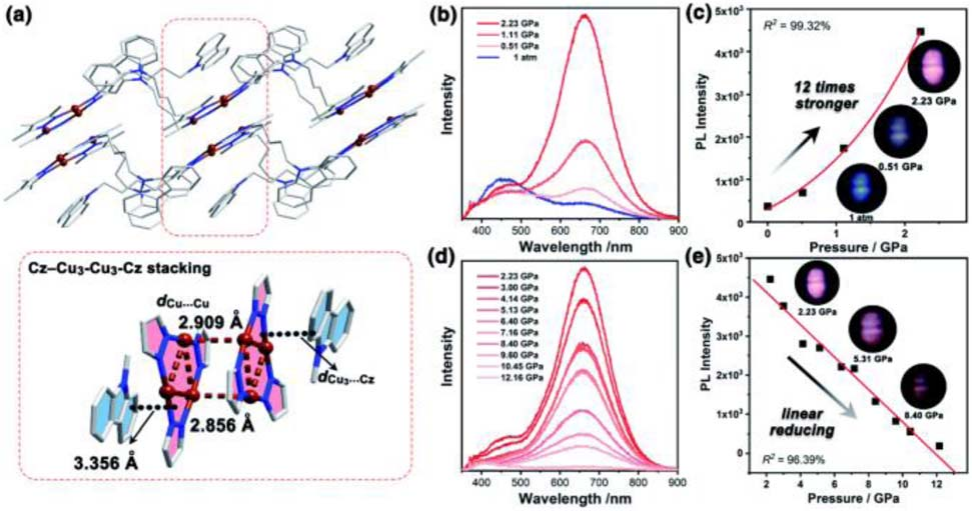
(a) The crystal packing of 3.35a at 100 K, showing discrete Cz–Cu_3_–Cu_3_–Cz stacking (Cu: brown, C: grey, and N: blue. H atoms are omitted for clarity). Emission spectra of 3.35a in a range of external pressures from (b) 1 atm–2.23 GPa and (d) 2.23–12.16 GPa at an excitation wavelength of 355 nm. The curve fitting function [*y* = *A*_1_ × exp(−*x*/*t*_1_) + *y*_0_, *R*^2^ = 99.32%] and linear fitting function [*y* = *A*_0_ + *k*_*x*_, *R*^2^ = 96.39%] of pressure and photoluminescence intensity for 3.35a in pressure ranges of (c) 1 atm–2.23 GPa and (e) 2.23–12.16 GPa (insets of (c) and (e): photoluminescence photographs at representative pressure points). Reprinted with permission from ref. 234 under the terms of the Creative Commons license. Copyright 2021, The Royal Society of Chemistry.

**Fig. 40:**
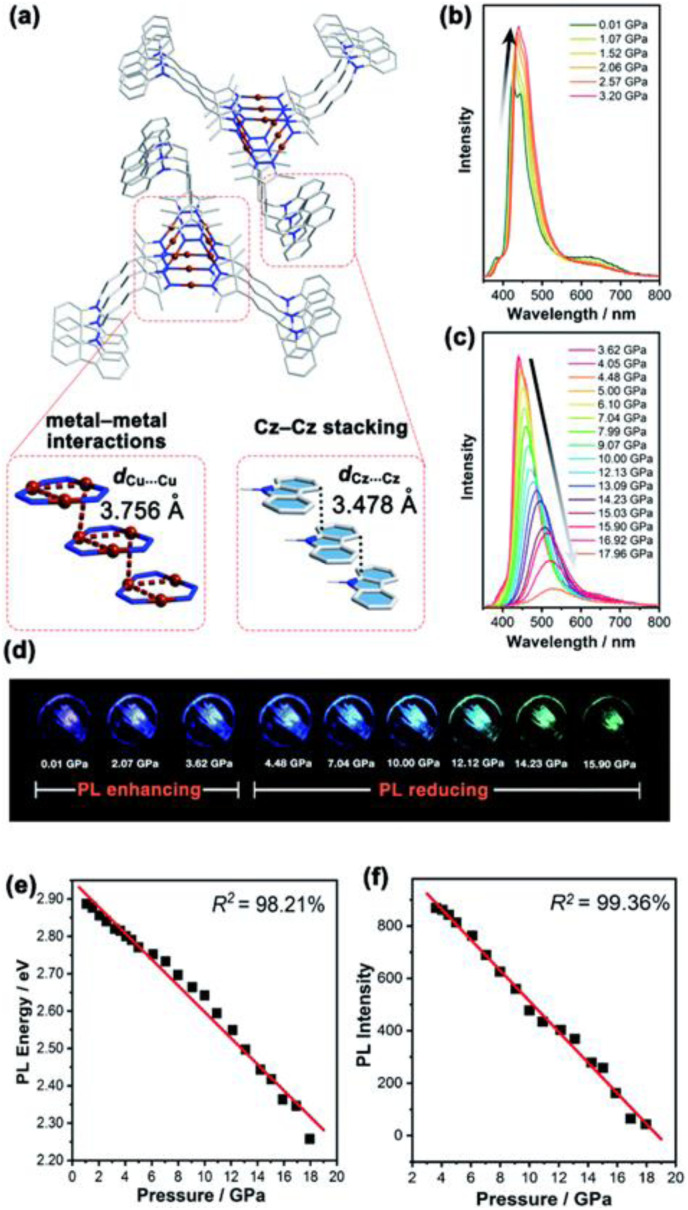
(a) The crystal packing of 3.35b at 298 K, showing the infinite chain stacking model of Cz and Cu_3_. The emission spectra of 3.35b in a range of external pressures from (b) 0.01–3.20 GPa and (c) 3.62–17.96 GPa at an excitation wavelength of 355 nm. (d) Photoluminescence photographs of the 3.35b crystal under compression up to 15.90 GPa. Linear fitting of the external pressure and (e) photoluminescence energy and (f) intensity of 3.35b. Reprinted with permission from ref. 234 under the terms of the Creative Commons license. Copyright 2021, The Royal Society of Chemistry.

The compound, prepared under solvothermal conditions, crystallized in two different polymorphs, 3.35a and 3.35b, exhibiting different molecular stacking. In particular, 3.35a is characterized by a packing motif showing dimers of trimers through strong intermolecular cuprophilic interactions (Cu⋯Cu distances of 2.85 and 2.91 Å) and weak Cz-Cu_3_ ones (3.36 Å) (Fig. 39a). Polymorph 3.35b, on the other hand, crystallized in stair-step infinite chains with weak intertrimer Cu⋯Cu interactions, relatively long intermolecular Cu⋯Cu distances of 3.76 Å at 298 K and the Cz units tightly stacked in a J-type aggregation along the *a*-axis through π–π interactions (Fig. 40a). The solid-state photoluminescence of 3.35a shows at r.t. a dual emission with a HE fluorescence at about 400 nm, originating from singlet excited states mainly localized on the Cz moieties, and a broad and more intense LE phosphorescence (Fig. 39b). The latter band comprises two components disclosed as the ^3^LC of the monomer (560 nm), whose intensity increases upon cooling to 200 K or lower temperature, and a metal-sensitized ligand centered phosphorescence mixed with the ^3^MLCT phosphorescence of the excimer (680 nm). Interestingly, when applying an isotropic hydrostatic pressure using a diamond anvil cell on 3.35a crystals, a rare example of pressure-induced phosphorescence enhancement (PIPE) was observed. Under 2.23 GPa pressure, the LE band intensity increases up to 12 times than under ambient conditions (10^−4^ GPa), while the HE emission intensity slightly diminishes, resulting in a remarkable color change from weak blue to bright pink. The process is fully reversible and, with the support of theoretical calculations, was interpreted as a result of an increase of the excimer contribution in the excited state due to the strengthening of the Cu_3_–Cz interactions and shortening of Cu_3_⋯Cz distances at high pressure (Fig. 39c–e).

For polymorph 3.35b, the packing features of the organic chromophore govern the emissive behavior of the crystals, quite resembling that of the proligand. Accordingly, the reversible piezochromic effect, characterized by a continuous red shift under external pressure, is similar to the piezoluminochromism displayed by Cz and confirmed the Cz-centred characteristics of 3.35b luminescence (Fig. 40b–f).

**Scheme 48:**
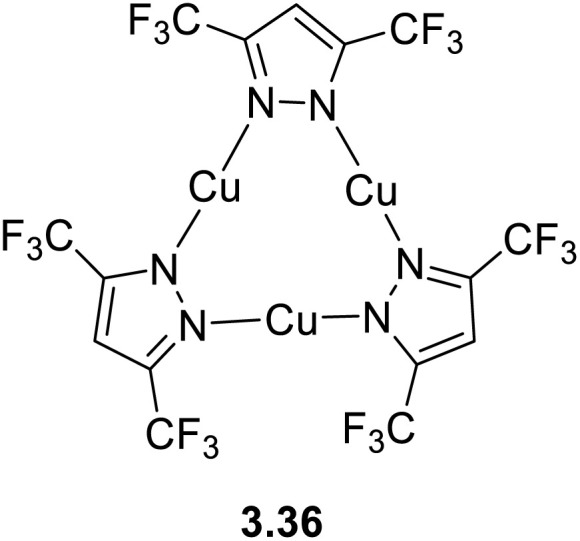
Chemical structure of 3.36.

The use of the diamond anvil cell was exploited by Lu *et al.*^235^ for the trimeric Cu(i) complex (tris[3,5-bis(trifluoromethyl)pyrazolatocopper(i)], Cu_3_Pz_3_, 3.36, Scheme 48), to modulate under isotropic pressure the cuprophilic interactions and define structure–property relationships associated with intramolecular and intermolecular Cu⋯Cu contacts. 3.36 was prepared by reacting copper(i) oxide and the corresponding pyrazole in solution.^236^

**Fig. 41:**
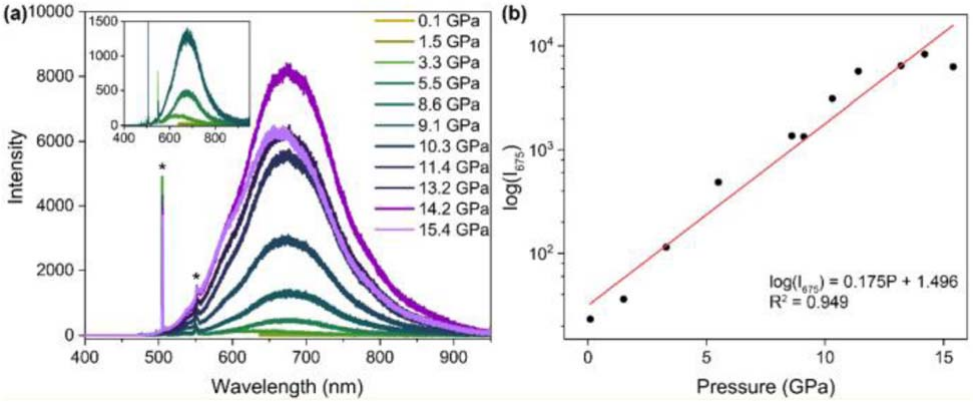
(a) *In situ* emission spectra of solid 3.36 under different pressures. Excitation wavelength: 473 nm. The sharp peaks near 500 and 550 nm (asterisk) arise from diamond and C–H stretching Raman modes, respectively. Inset: zoom-in emission spectra of 3.36 under variable pressures from 0.1 to 9.1 GPa. (b) Logarithmic plot of the intensity at 675 nm as a function of pressure. Reprinted with permission from ref. 235. Copyright 2023, American Chemical Society.

High-pressure PXRD studies on 3.36, supported by FT-IR and Raman measurements and DFT calculations, disclosed a distinctive contraction of the intertrimeric Cu⋯Cu distances from 3.88 Å under ambient conditions to estimated 2.66/2.67 Å, well below the sum of Cu(i) vdW radii, under compression. The process is reversible, and the original structure is fully recovered upon releasing the pressure. Moreover, experimental and theoretical results revealed a slight increase of the intramolecular Cu⋯Cu contacts under pressure, highlighting the anisotropic nature of intratrimeric Cu⋯Cu interactions. The emissive properties of 3.36, characterized by a metal-centered (^3^MC) phosphorescence of intertrimer origin, are strongly affected by such changes in the cuprophilic interactions, revealing PIPE features under pressure. In particular, the weak and broad emission at 675 nm increased up to 3 orders of magnitude under ambient pressure to 15 GPa, displaying a reversible turn on/off photoluminescence. Such behavior was ascribed to a reduction of non-radiative decays in the more rigid structure under pressure (Fig. 41).

### 6.3 Vapochromism

A luminescent change in response to exposure to VOCs or gases is indicated as vapochromism or, more specifically, vapoluminescence. The interaction with the vapor molecule can alter the electronic transitions of the host material through either a modification of the pattern of intermolecular interactions (including π stacking, hydrogen-bonding and metallophilic interactions), a change in the coordination sphere *via* coordinated substitution or vapor mediated structural interconversion.^237^ Vapoluminescent materials are of great significance due to their potential application in different fields spanning from air quality assessment to disease diagnosis.^237^ In this regard, Ford *et al.* reported the first Cu(i) vapochromic compounds, namely [CuI(4-pic)]_*n*_ (*n* = 4; ∞; 4-pic = 4-picoline), whose tetramer–polymer reversible interconversion was accomplished by exposure to solvent vapors.^238^

#### 6.3.1 Advancements during 2020–mid-2025

**Scheme 49:**
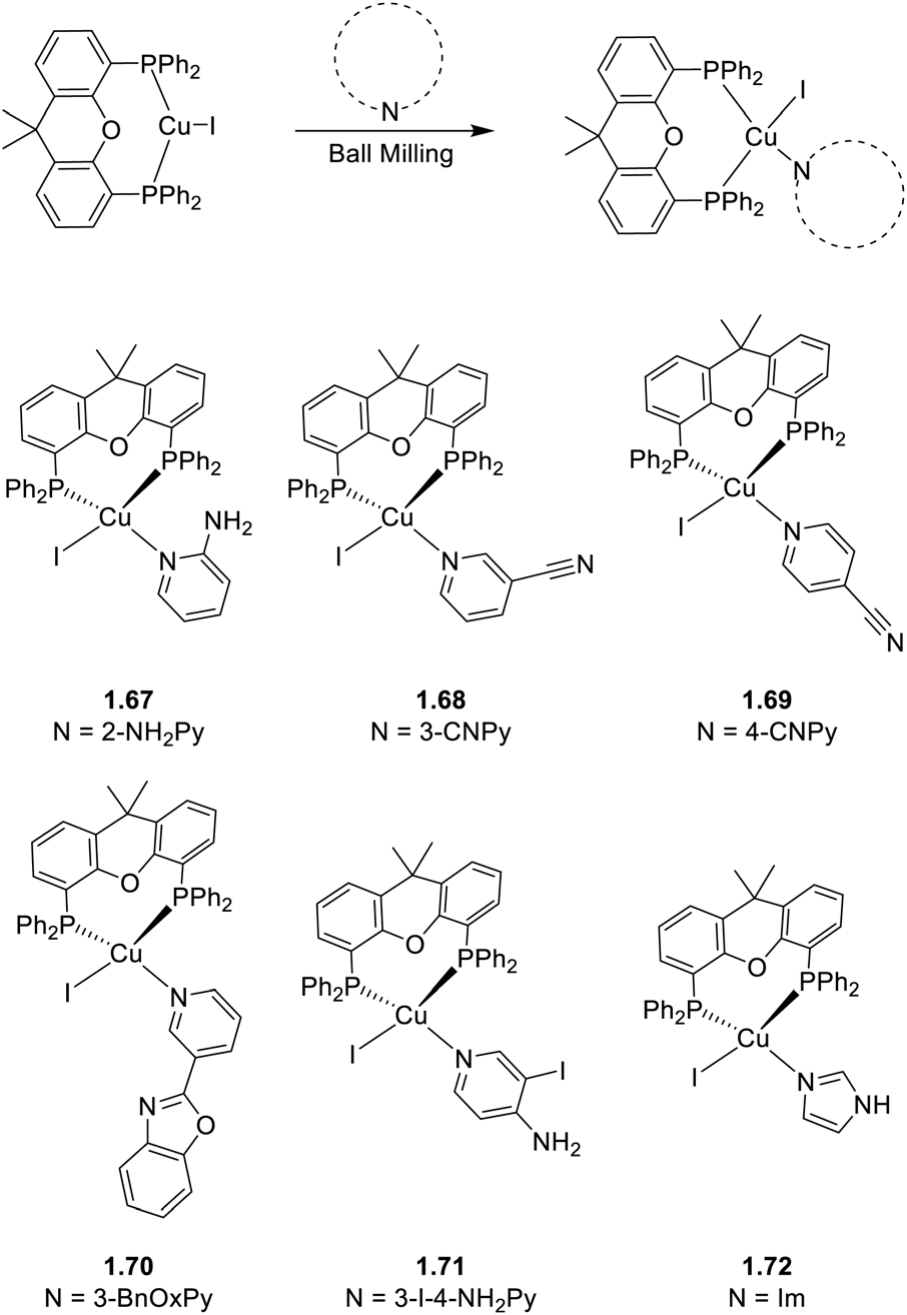
Synthesis of 1.67–72.

A series of mononuclear complexes of general formula [CuIL_*N*_(P^P)] (P^P = xantphos; L_*N*_ = 2-aminopyridine, 1.67, 3-cyanopyridine, 1.68, 4-cyanopyridine, 1.69, 2-(3′-pyridyl)-benzoxazole, 1.70, 3-I-4-aminopyridine, 1.71, and Im, 1.72), were prepared by a solvent assisted ball milling reaction (Scheme 49).^239^

The compounds, both as powders or dispersed PMMA films, are characterized by broad 3(M + X)LCT phosphorescence, with maxima in the 490–650 nm interval according to the electron donating or withdrawing ability of the N-ligands. Among the series, structural rigidity was found to increase *Φ* (up to 44.29% for complex 1.70), while the presence of the iodine atom in complex 1.71 facilitates ISC, leading to the weakest emission (*Φ* = 0.11%). The vapochromic features of all derivatives were investigated by exposing paper strips embedded with the Cu(i) complexes dispersed in PMMA to vapors of different VOCs. Photoluminescence enhancement was observed for all compounds in a Py or 4-pic atmosphere, with 1.71 performing as the best sensor, showing *I*/*I*_0_ values of up to 50 and 140 and LODs of 2.51 and 7.33 ppm, respectively. It has to be noted that the response sensitivity of the materials is related to their initial luminescent efficiency with a significant “lighting-up” effect for 1.71 due to its initial weak luminescence. On the basis of TDDFT calculation and by analogy with the related model complex [CuI(P^P)] (P^P = xantphos), the mechanism for Py detection was identified in ligand substitution with formation of the strongly emissive [CuIPy(P^P)] species. Complex 1.71 resulted as an efficient sensor also for TEA with a luminescence enhancement of 15 times after 2.5 min, a saturation value of up to 25 after 15 minutes and a concomitant blue shift from 620 nm to 510 nm. In the latter case, the mechanism was rationalized with the formation of a supramolecular structure between 1.71 and TEA that inhibits the nonradiative decay of the original complex. All complexes, except for 1.71 which is too weakly emissive for a clear response, display significant quenching and a blue shift of their emission in the detection of cyclohexylamine (CHA) and THF, through a substitution mechanism similar to the one proposed for Py. The most significant response was observed for 1.69, characterized by the appearance, within a few seconds, of a band at 480 nm due to the formation of substituted products or a supramolecular adduct with the VOC species, providing a ratiometric detection mode by comparison with the original peak at 630 nm. Sensing of non-coordinating substances such as DCM and acetone produced a fast decrease in the luminescence intensity, especially for 1.68 and 1.69, which was ascribed to the vapor molecules acting as quenchers.

**Scheme 50:**
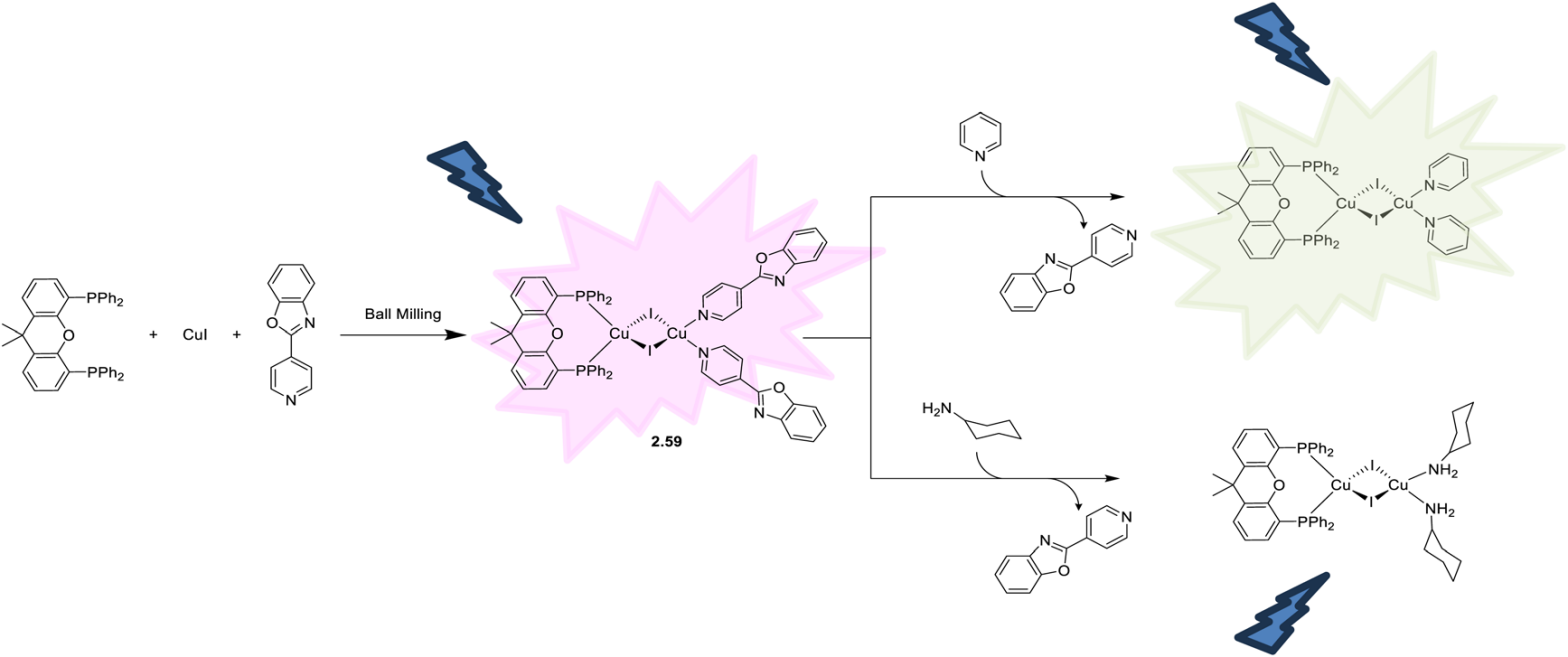
Synthesis of 2.59 and the proposed mechanism for Py and CHA sensing.

The same research group also reported the dimeric [(Cu_2_I_2_)(N^N)(P^P)] (P^P = xantphos; N^N = 4-(2-benzoxazole)pyridine, 4-PBO), 2.59, prepared by the ball-milling method (Scheme 50).^240^ At r.t., powders of 2.59 possess bright red emission at 645 nm which, according to TDDFT calculations, was assigned to ^1/3^(M + X)LCT (from the cluster core to the 4-PBO ligand) excited states with Δ*E*_ST_ energy splitting compatible with TADF (0.0725 eV). Doped (5%) PMMA films show emission at 620 nm shifted to 635 nm when loaded onto a paper strip, which was used to develop vapochromic sensors towards Py and CHA. In the latter case, a substitutional response mechanism results in quenching of the emission which reaches its maximum (90%) after 15 min of exposure with an assessed LOD of about 16.714 ppm and the appearance of two weak emissions at 600 and 450 nm assigned to the CHA-substituent and free 4-PBO ligand, respectively. Response to Py occurs through a more complicated trend first characterized by quenching and, successively, by intensification of the new emission at 510 nm again assigned to a substituted product. LODs for the photoluminescence-quenching and enhancement intervals are about 344 and 630 ppm, respectively. Moreover, experiments with other common VOCs (formaldehyde, benzene, alcohols, ammonia, other amines and other pyridines) revealed good selectivity towards CHA and Py.

**Fig. 42:**
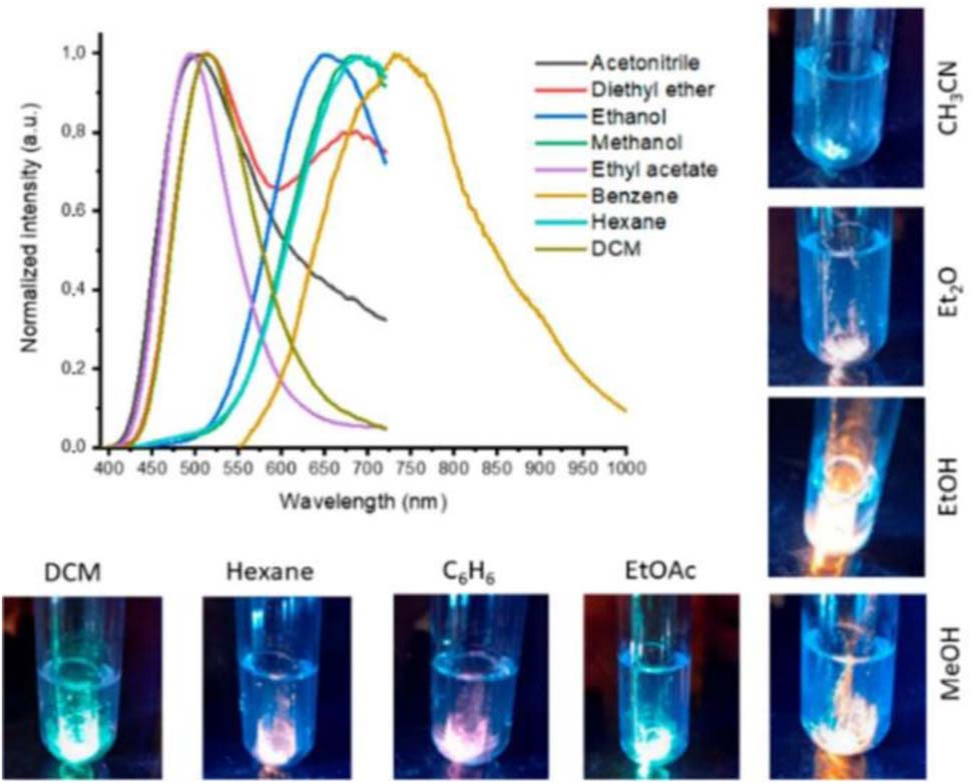
Emission spectra of the crystalline phase 4.26 saturated with various solvents and photos of the samples under UV light. Reprinted with permission from ref. 169. Copyright 2023, John Wiley and Sons.

An example of vaporesponsive tetranuclear octahedral Cu(i) derivative, 4.26, was reported in 2023.^169^ The compound, whose structure/photophysical properties relationship has been discussed in Section 5.1, displays strong vapochromic response by exposure to a wide range of VOCs (Fig. 42).^169^ This behavior was ascribed to the conformational lability of the complex, which undergoes, as deduced by XRD studies, interconversion between a “compact” and a “stretched” form of the Cu_4_I_4_ core according to different co-crystallized solvent molecules. The two conformations result in quite different emission colors, the green one being assigned to the “compact” and the red one to the “stretched” form.

### 6.4 Multistimuli responsiveness

**Scheme 51:**
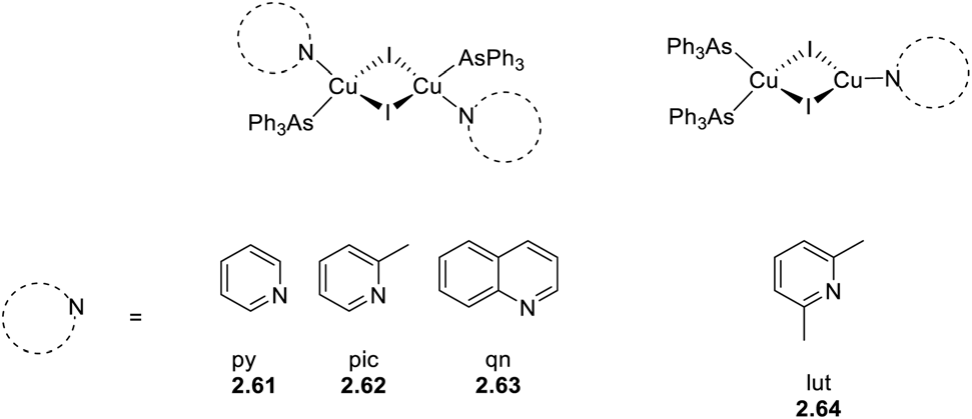
Chemical structure of 2.61–64.

**Fig. 43:**
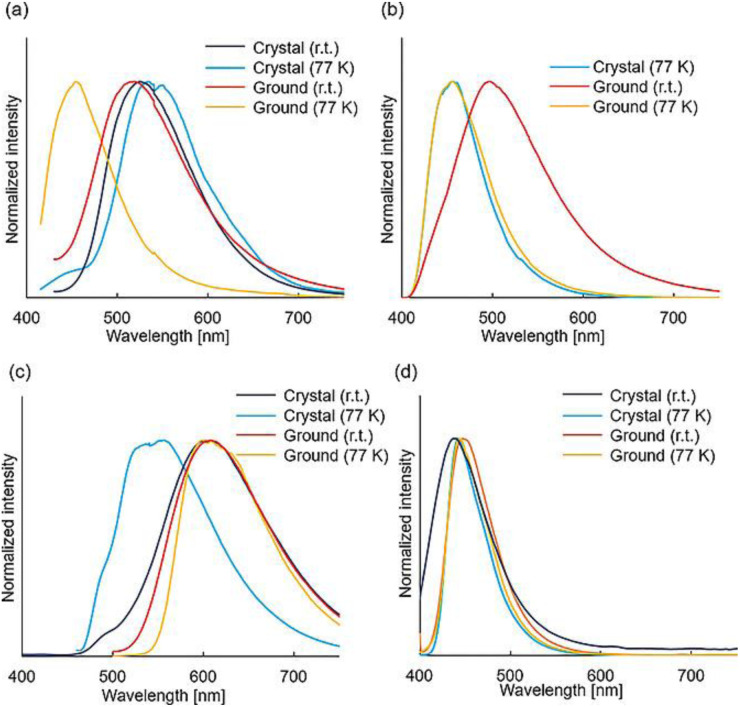
Photoluminescence spectra of (a) 2.61, (b) 2.62, (c) 2.63, and (d) 2.64 in the solid state. Reprinted with permission from ref. 241. Copyright 2020, John Wiley and Sons.

Multistimuli-responsive derivatives of Cu(i), although highly desirable, are still limited, and their number becomes even lower when excluding polymeric and ionic complexes. Among these, a family of thermo- and mechanochromic dinuclear rhomboid Cu_2_I_2_ complexes of general formula [(Cu_2_I_2_)(AsPh_3_)_2_L_2_] (L = py, 2.61, 2-pic, 2.62, qn, 2.63, and 2,6-lutidine (lut), 2.64, Scheme 51) was reported in 2020 by Kobayashi *et al.*^241^ SCXRD analysis revealed that the Cu⋯Cu distances are affected by the steric hindrance of the N-heteroaromatic co-ligand, with the bulkier co-ligand giving the longer distances due to steric repulsion: 2.62 (3.24 Å) > 2.63 (2.90 Å) > 2.61 (2.78 Å). On the other hand, the shortest Cu⋯Cu distance (2.69 Å) is observed for 2.64, in which only one N-ligand is coordinated to a Cu(i) atom. In addition, a comparison with the Cu⋯Cu distance of 2.61 (2.97 Å), pointed out the positive effect of a weaker σ-donor like arsenic, with respect to phosphorus, for the formation of cuprophilic interaction. Accordingly, at r.t., 2.61 is characterized by LE phosphorescence (525 nm; *τ* = 14.4 μs and *Φ* = 8.0%) from a ^3^CC excited state attributed to cuprophilic interactions (Fig. 43a). This band, although slightly red-shifted due to a shrinkage of the Cu⋯Cu distance, is still the predominant one at 77 K, together with the appearance of a HE emission at 450 nm due to the population of a ^3^(M + X)LCT state. A stronger thermochromic effect is recorded for 2.63, showing emissions from ^3^CC (601 nm; *Φ* = 12.0%) and ^3^(M + X)LCT (557 nm; *Φ* = 40.0%) excited states at r.t. and 77 K, respectively (Fig. 43c). For 2.62, characterized by a significantly long Cu⋯Cu distance, no emission is observed at r.t., while a band of ^3^(M + X)LCT origin (455 nm; *Φ* = 65.0%) appears at 77 K (Fig. 43b). Interestingly, a different behavior is observed for 2.64 in which the mono-coordination seems to favor the emission from ^3^(M + X)LCT by hampering the IC between the ^3^(M + X)LCT and the ^3^CC excited states (Fig. 43d). Moreover, the Cu⋯Cu distances can be modified by grinding, affecting the energy levels of the ^3^CC excited states and unveiling the mechanochromic behavior of these compounds. For all derivatives, a crystal-to amorphous transition was disclosed by means of PXRD analysis that shows weaker and broader diffraction peaks for the ground samples. In particular, ground 2.61 displays at 77 K only the HE emission instead of the dual emission observed for the pristine material. This result was interpreted on the basis of the enhancement of the energy barrier between ^3^(M + X)LCT and ^3^CC excited states caused by mechanical forces. Non-emissive crystals of 2.62 become quite emissive (496 nm and *Φ* = 7.0%) at r.t. after grinding, probably due to a shortening of the Cu⋯Cu distance. Ground 2.63 displays a red-shifted, intensified emission band. In contrast, 2.64, characterized by the shortest Cu⋯Cu distances, does not show MCL either at room or low temperature.

**Fig. 44:**
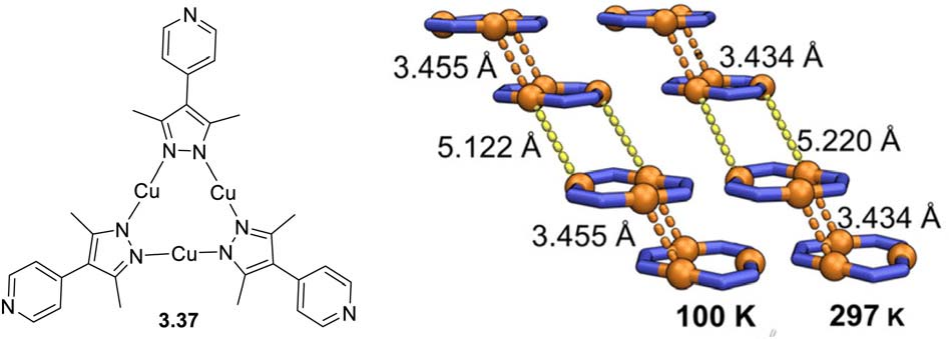
Left: chemical structure of 3.37. Right: the face-to-face stacking diagrams shown by Cu_3_N_6_ rings and intermolecular Cu⋯Cu distances at 100 K and 297 K. Colour code: orange, Cu; blue, N. Adapted with permission from ref. 242. Copyright 2023, The Royal Society of Chemistry.

Based on the observation that emissive properties of CTCs strongly depend on intermolecular interactions (see Section 4), in 2023, Tang *et al.*^242^ re-investigated the complex [Cu(^4^PyPz)]_3_ (3.37) (first reported by Huang in 2008),^243^ as a promising candidate for multistimuli-responsive luminescence (Fig. 44). According to this investigation, the already reported thermochromism of 3.37 was implemented with mechanochromic, solventochromic (change in luminescence of a solid by treatment with a non-solvent liquid),^238^ vapochromic and excitation-wavelength-dependent emissive properties. Moreover, interpretation of the reversible thermochromic phenomenon was supported by SCXRD analysis performed at 297 and 100 K, which revealed significant elongation of intermolecular Cu⋯Cu distances at low temperature (from 3.434 to 3.455 Å), suggesting concomitant dimer dissociation.

**Fig. 45:**
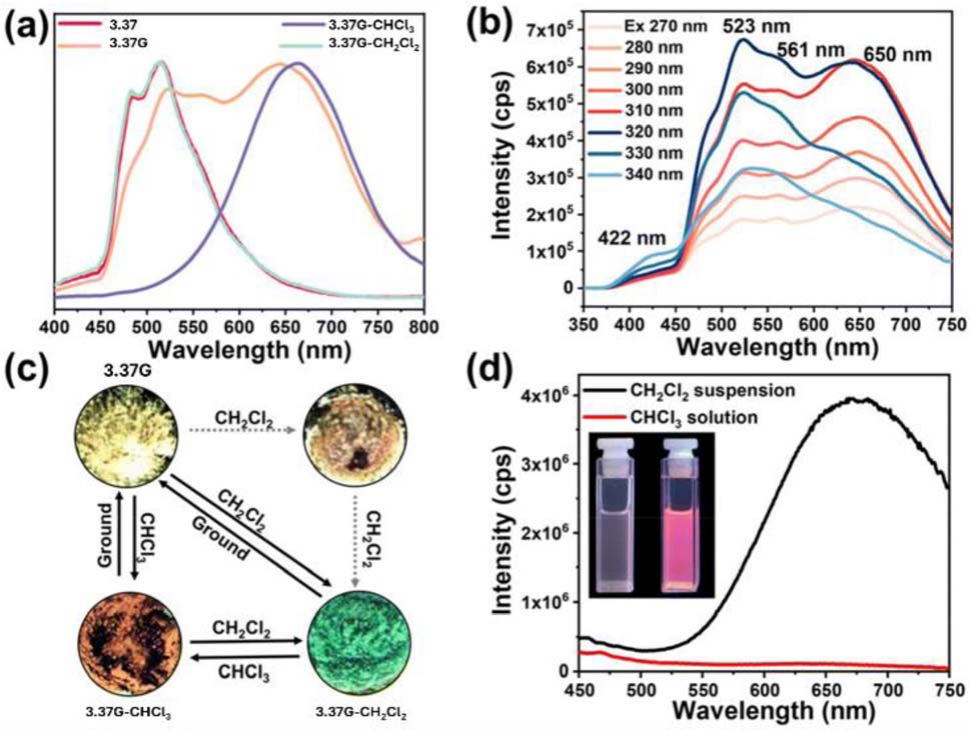
(a) Normalized room-temperature emission spectra of crystalline 3.37, 3.37G, 3.37G-CH_2_Cl_2_ and 3.37G-CHCl_3_ upon excitation at 320 nm. (b) The excitation-wavelength-dependent emission spectra of 3.37G at r.t. (c) Photographs demonstrating a series of treatments to solid-state 3.37 under excitation of UV light (320 nm). The solid line represents the final state of transformation, while the dashed line means there exists an intermediate state during the transformation. The dashed line indicates the process of rapid transformation in 3–5 seconds, while the solid line represents the normal transformation. (d) Room-temperature emission spectra of the suspension of 3.37 in DCM and CHCl_3_. Photographs demonstrating the emission colours of 3.37 in CHCl_3_ and DCM suspension (inset, left: CHCl_3_ suspension; right: DCM suspension). Adapted with permission from ref. 242. Copyright 2023, The Royal Society of Chemistry.

At r.t., crystals of 3.37 exhibit excitation dependent dual emission comprising a minor contribution at 415 nm and a prevailing phosphorescence at 485–515 nm, resulting in a green coloured emission (Fig. 45a). The LE emission was assigned, in agreement with experimental results and DFT/TDDFT calculations, to deactivation from a metal-sensitized ligand-localized monomeric excited state. At 77 K, emission peaks are red-shifted by about 60 nm (at 523 nm and 570 nm, respectively), and their original positions are restored upon warming the sample. It was also observed that mechanical stress (through grinding) induces a broadening and a bathochromic shifting of the emissions (Fig. 45a and b).

The ground sample, 3.37G, displays an excitation dependent dual emissive behavior with components at 523 and 650 nm resulting in yellow emission. At high energy excitation (270–320 nm), the 650 nm emission prevails, while lowering the excitation energy (340 nm) results in intensification of the 523 nm component. The reversibility of the grinding process was accomplished through exposure of 3.37G to organic solvents or vapours, in particular DCM and chloroform, demonstrating the formation of metastable intermediate red emissive species 3.37G-CH_2_Cl_2_ and 3.37G-CHCl_3_, respectively (Fig. 45a and b). Their effective existence was confirmed by DFT calculations and subsequent electrostatic potential and energy decomposition analyses, revealing the establishment of explicit C–H⋯N HB interaction between a solvent molecule and the pyridinic nitrogen atom of 3.37, which is stronger with chloroform than with DCM. TDDFT calculations on the optimized 3.37G-CH_2_Cl_2_ and 3.37G-CHCl_3_ species revealed a stabilization, greater for the latter, of both S_1_ and T_1_, explaining the red-shifted emission of the solvated forms. The stress-induced responsive behaviour of 3.37 was exploited to prepare two luminescent logic gate and microarray data for information writing and erasing and for anti-counterfeit devices.

## 7. Conclusions

Cu(i)-based materials exhibit remarkably rich photophysical behavior, stemming not only from their diverse structural architectures but also from their responsiveness to various external stimuli. These distinctive features, combined with copper's natural abundance, low cost, and minimal toxicity, make Cu(i) derivatives especially attractive for a wide range of applications. This is evidenced by the growing number of Cu(i)-based materials developed for optoelectronic technologies. While earlier studies predominantly focused on tetranuclear photoluminescent clusters, recent years have seen a significant rise in the exploration of lower-nuclearity complexes. This shift has unlocked a broad spectrum of applications in optoelectronic devices, including OLEDs, LECs, chemical sensors, organic laser resonators, and X-ray scintillators.

This review highlights advances during 2020–mid-2025 in neutral 0D Cu(i) complexes coordinated with N-donor ligands, which play a pivotal role in tuning the structural, electronic, and optical properties, and in some cases, influencing the nuclearity of the complexes. For each family, details relative to synthetic methods, structural features and the nature of the excited states involved in the emissive processes (the latter is also summarized in tables reported in the SI) are collectively discussed, offering a comprehensive overview of the challenges addressed in this field. Common issues associated with Cu(i) systems, such as structural rearrangements in the excited state that diminish emission efficiency and facile interconversion between nearly isoenergetic isomers that complicates synthesis, can be effectively mitigated through strategic ligand design. Approaches exploiting rigid chelating frameworks, steric hindrance, and tailored electronic properties have proven effective in overcoming these limitations.

## Author contributions

All authors wrote, revised and finalized the manuscript.

## Conflicts of interest

There are no conflicts to declare.

## Supplementary Material

SC-016-D5SC04685H-s001

## Data Availability

No primary research results, software or code have been included and no new data were generated or analysed as part of this review. Supplementary information: Tables S1–S7 with a summary of the photophysical properties of the described compounds. See DOI: https://doi.org/10.1039/d5sc04685h.
