# Supplementary material for: Recent advances in mono- and multi-nuclear photoluminescent Cu(i) complexes with nitrogen containing ligands and their stimuli responsiveness
Source: Chem Sci. 2025 Oct 17;16(44):20755–805. doi: 10.1039/d5sc04685h (PMC12581026; doi:10.1039/d5sc04685h)
Supplement: SC-016-D5SC04685H-s001 [file SC-016-D5SC04685H-s001.pdf]

Supplementary Information (SI) for Chemical Science.
This journal is © The Royal Society of Chemistry 2025

Recent advances in mono- and multi-nuclear photoluminescent Cu(I) complexes with nitrogen containing ligands and their stimuli responsiveness

Alessandra Forni,[a] Daniele Malpicci,*[a,b] Elena Lucenti,[a] Luca Zecchinello,[a,b] Alessia Colombo,[b] and Elena Cariati*[a,b]

Table S1: Summary of the photophysical properties of NHC-Cu-L compounds.

Carbene	Amide	Physical State	λ_{em} (nm)	τ_{av} (μs)	Φ (%)	HOMO (eV)	LUMO (eV)	Ref
**1.1**	**a**	2-MeTHF[a]	542	1.1	55	-4.17	-1.99	1, 2
		PS Film	506	1.4	90	-	-	
	**d**	Toluene[a]	574	0.72	34	-4.08	-1.99	3
	**h**	Toluene[a]	517	0.53	40	-5.6	-1.98	4
		PMMA Film	483	4.5	94	-	-	
	**i**	Toluene[a]	482	0.85	32	-5.28	-1.91	4
		PMMA Film	462	114	80	-	-	
	**kO**	Toluene[a]	552	0.37	29	-5.49 (-4.57 calc)	-2.85 (-1.94 calc)	5
		mCBP film	514	0.84	47	-	-	
	**kS**	Toluene[a]	569	0.29	32	-5.38 (-4.47 calc)	-2.88 (-2.01 calc)	5
		mCBP film	525	0.79	70	-	-	
	**m**	PS film	505	0.55	19	-	-	6
	**r**	Toluene[a]	638	0.11	12	-5.27	-3.43	7
		PS Film	609	0.42	24			
	**aa**	Toluene[a]	556	0.71	70	-4.23	-2.08	3
	**ab**	Toluene[a]	607	0.088		-5.47	-2.86	8
		mCP Film	559	0.4	40	-	-	
	**ac**	Toluene[a]	551	0.254		-5.54	-2.64	8
		mCP Film	518	27.73	48	-	-	
	**adO**	Toluene[a]	552	0.37	29	-5.49 (-4.57 calc)	-2.85 (-1.94 calc)	5
		mCP Film	514	0.84	47	-	-	
	**adS**	Toluene[a]	569	0.29	32	-5.38 (-4.47 calc)	-2.88 (-2.01 calc)	5
		mCBP film	525	0.79	70	-	-	
	**ae**	PS Film	560	0.19	5	-	-	6
	**af**	PS Film	560	0.18	8	-	-	6
	**ag**	PS Film	-	0.82	12	-	-	6
	**ah**	PS Film	459	1.9	74	-	-	6
**1.2**	**a**	PS Film	534	1.5	58	-	-	9
	**b**	PS Film	482	1.4	77	-	-	9
**1.3**	**a**	PS Film	556	0.97	70	-	-	9
	**b**	PS Film	500	1.1	83	-	-	9
**1.4**	**a**	Toluene[a]	502	0.71	>95	-	-	10
		PS Film	459	1.24	93	-	-	
**1.5**	**a**	Toluene[a]	595	0.56	47	-	-	10
		PS Film	594	0.95	75	-	-	

**1.6**	**a**	Solid State	400	0.016	32.6	-	-	11
			550	0.024		-	-	
			550-750	55000		-	-	
	**n**	Solid State	450	0.0045	10	-	-	12
			504	1440		-	-	
	**o**	Solid State	452	0.0043	20	-	-	12
			507	2100		-	-	
	**p**	Solid State	Non emissive	-	-	-	-	12
	**q**	Solid State	Non emissive	-	-	-	-	12
**1.7**	**a**	Toluene[a]	450	1.5	75	-4.92	-1.47	13
		2-MeTHF[a]	458	2.06	35	-	-	
		PS Film	434	4.47	86	-	-	
**1.8**	**a**	Solid State	400	0.011	12.7	-	-	11
			550	0.024		-	-	
			750	32000		-	-	
**1.9**	**a**	Toluene[a]	511	0.58	37	-5.08	-2.72	7
		Crystals	471	2.6	74			
	**c**	Toluene[a]	517	0.75	42	-5.09	-2.81	7
		Crystals	478	1.9	82			
**1.10**	**a**	Toluene[a]	508	0.36	41	-5.15	-2.78	7
		Crystals	467	1.9	68			
	**c**	Toluene[a]	513	0.85	79	-5.12	-2.82	7
		Crystals	474	2.1	84			
**1.11**	**a**	Toluene[a]	521	0.46	39	-5.16	-2.82	7
		Crystals	479	2.1	71			
	**c**	Toluene[a]	527	0.41	23	-	-	7
		Crystals	484	1.6	78			
	**d**	Toluene[a]	549	0.72	73	-5.02	-2.83	7
		Crystals	499	1.6	73			
	**e**	Toluene[a]	554	0.76	76	-4.96	-2.83	7
		Crystals	509	1.2	86			
**1.12**	**a**	THF[a]	509	2.7	88	-	-	14
		PMMA	472	15.19	53			
	**d**	THF[a]	537	1.8	65	-	-	14
		PMMA	485	7.89	13			
	**f**	THF[a]	537	1.5	47	-	-	14
		PMMA	485	3.12	39			
	**g**	THF[a]	540	0.86	38	-	-	14
		PMMA	484	3.63	65			
	**t**		Not stable	-	-	-	-	14
	**u**	THF[a]	715	0.032	1	-	-	14
		Toluene[a]	698	0.048	1			
		PMMA	601	0.68	29			
**1.13**	**v**	THF[a]	530	1.6	52	-	-	14
	**ai**	THF[a]	550	1.3	36	-	-	14
		PMMA	498	2.81	13			
**1.14**	**a**	Toluene	621	-	-	-4.9	-2.58	15

This journal is © The Royal Society of Chemistry 20xx

		DMIC-TRZ Film	574	0.44	76			
	**l**	Toluene	617	-	-	-4.96	-2.61	15
		DMIC-TRZ Film	550	1.01	86			
	**aj**	Toluene	616	-	-	-4.96	-2.65	15
		DMIC-TRZ Film	562	12.4	69			
**1.15**	**a**	Toluene[a]	627	-	-	-4.96	-2.65	15, 16
		DMIC-TRZ Film	564	0.65	84			
		Toluene[a]	624	0.18	29	-4.84	-2.59	16
		mCBP Film	567	0.41	88			
	**b**	Toluene[a]	555	0.36	58	-5.01	-2.49	16
		DMIC-TRZ Film	508	0.41	89			
	**l**	Toluene	627	-	-	-4.96	-2.65	15
		DMIC-TRZ Film	564	0.65	84			
	**d**	Toluene[a]	660	0.11	14	-4.7	-2.51	16
		DMIC-TRZ Film	581	0.37	66			
	**aa**	Toluene[a]	635	0.12	15	-4.79	-2.52	16
		DMIC-TRZ Film	568	0.36	76			
**1.16**	**a**	Toluene[a]	502	0.55	74	-4.84	-1.8	16
		DMIC-TRZ Film	470	0.47	52			
**1.17**	**j**	Toluene[a]	500	3.8	12	-5.62	-2.5	17
		oCBP Film	477	3	56			
		Crystals	483	22.5	41			
	**u**	Toluene[a]	661	0.11	-	-4.8	-2.72	18
		PS Film	614	-	-			
	**ak**	Toluene[a]	489	1.13	49.5	-5.45	-2.78	18
		PS Film	458	8.52	25.6			
	**al**	Toluene[a]	589	0.58	19.5	-4.96	-2.55	18
		PS Film	528	9.6	36.8			
	**am**	Toluene[a]	607	0.43	3	-5.11	-2.61	18
		PS Film	490	4.1	1.7			
**1.18**	**a**	Toluene	556	1.37	3.6	-	-	19
		Neat film	557	16	10			
		1% Zeonex Matrix	519	9.8	6			
**1.19**	**a**	Toluene	502	3.3	100	-5.42	-2.51	19
		Neat Film	488	4	23			
		1% Zeonex Matrix	493	5	64			
**1.20**	**a**	Toluene	660	0.187				
		1 % PS matrix	565	1.88	65	-5.49	-3.08	20
		MeTHF glasses	500	109.45				
**1.21**	**a**	Solid State	621	0.42	32	-	-	21
		PMMA	638	0.397	27			
**1.22**	**a**	Crystals	437	0.044	11	-5.42	-1.59	22

This journal is © The Royal Society of Chemistry 20xx

			594	0.68				
		Solid State	570	0.6	26			
	**d**	Crystals	451	0.041	17	-5.31	-1.49	22
			588	0.29				
		Solid State	539	1	40			
**1.23** ***Rac***	**s**	THF/H_2O (90%)	489	3	36	-	-	23
**1.23-*SS***	**s**	THF/H_2O (90%)	497	1.3	41	-	-	23
**1.23-*RR***	**s**	Crystal Flattened	495	1.625	42	-	-	23
		Crystal Croocked	508	1.67	39			23
**1.24**	**n**	Solid State	450	0.00577	20	-	-	12
			510	410				
	**o**	Solid State	452	0.00523	19.2	-	-	12
			510	3370				
	**p**	Solid State	427	0.00549	5	-	-	12
			480	630				
	**q**	Solid State	560	0.45	11.5	-	-	12

[a]In oxygen free conditions

This journal is © The Royal Society of Chemistry 20xx

Please do not adjust margins

Table S2: Summary of the photophysical properties of tetrahedral and trigonal monomeric complexes in the solid state.

	RT			LT					
**Compound**	**Φ (%)**	**λ_{em} (nm)**	**τ_{av} (μs)**	**Φ (%)**	**λ_{em} (nm)**	**τ_{av} (μs)**	**HOMO (eV)**	**LUMO (eV)**	**Ref**
**1.25**	41.7	559	14.2	-	558	32.5	-	-	24
**1.26**	88.3	529	55.2	-	521	75	-	-	24
**1.27**	31.1	556	12.28	-	555	33.7	-	-	24
**1.28**	41.3	577	4.8	-	564	27.8	-	-	24
**1.29**	70.5	533	19.5	-	522	58.5	-	-	24
**1.30**	34.6	564	10.5	-	556	39	-	-	24
**1.31**	35	586	5.8	-	-	-	-	-	25
**1.32**	27	589	10.6	-	-	-	-	-	25
**1.33**	30	568	4.5	-	-	-	-	-	25
**1.34**	83	541	9	85	551	3300	-4.08	-2.28	26
**1.35**	82	530	7	85	530	420	-4.04	-2.3	26
**1.36**	90	540	5	90	540	680	-3.67	-2.27	26
**1.37**	4.2	298	462	63.2	77	476	-	-	27
**1.38**	85	472	7.5	-	499	46.9	-	-	27
**1.39**	81	598	46	-	-	-	-	-	28
**1.40**	37	564	21.3	43.6	592	105.5	-5.69	-2.79	29
**1.41**	18.9	561	18.9	59	586	80.3	-5.72	-2.86	29
**1.42**	67.4	570	23.5	73.1	603	103.3	-5.74	-2.85	29
**1.43**	<1	550	0.011	-	-	-	-5.16	-1.25	30
**1.44**	<1	680	0.222	-	-	-	-6.14	-1.24	30
**1.45**	77	470	20.4	-	-	-	-	-	31
**1.46**	92	467	34.48	-	-	-	-	-	31
**1.47**	78	480	21.65	-	-	-	-	-	31
**1.48**	66	444	13.18	-	-	-	-	-	31
**1.49**	76	473	10.23	-	-	-	-	-	31
**1.50**	39	452	54.93	-	-	-	-	-	31
**1.51**	95	503	22.91	-	-	-	-5.23	-1.39	32
**1.52**	87	528	18.21	-	-	-	-5.2	-1.31	32
**1.53**	-	-	4.94	86	494	30	-5.127	-1.918	33
**1.54**	-	-	11.35	85	476	31.3	-5.046	-1.83	33
**1.55**	-	-	13.8	59	489	36.8	-5.1	-1.89	33
**1.56**	-	-	14.37	73	512	126	-5.09	-1.754	33

This journal is © The Royal Society of Chemistry 20xx

Please do not adjust margins

Please do not adjust margins

Table S3: Summary of the photophysical properties of dimeric complexes in the solid state.

	RT				LT (77K)				
Complex	d_{Cu-Cu} (Å)	Φ (%)	λ_{em} (nm)	τ_{av} (µs)	Origin	Φ (%)	λ_{em} (nm)	τ_{av} (µs)	Ref
**2.1**	-	43.3	520	12.5	-	-	-	-	34
**2.2**	-	13.5	560	6.2	-	-	-	-	34
**2.3**	-	5.1	650	1.4	-	-	-	-	34
**2.4**	-	59.6	515	1.9	-	-	-	17.3 (10K)	35
**2.5**	2.4728 - 2.5158	66.5	483	-	CC	-	-	-	36
**2.6**	2.8926	35.1	606	9.9	^{1}MLCT	78.1	625	25	37
**2.7**	2.827	27.5	591	9.5	^{1}MLCT	58.2	612	21.9	37
**2.8**	3.037	52	543	10.7	^{1}MLCT	88.2	568	134.7	37
**2.9**	2.9904	28.2	507	8.6	^{1}MLCT	55.3	527	17.8	37
**2.10**	2.8627	15.6	528	8.3	^{1}MLCT	38.3	528	23.4	37
**2.11**	3.0708	49	558	16.4	^{1}(M+X)LCT	60	576	79.2	38
**2.12**	3.011	44	575	16.1	^{1}(M+X)LCT	65	602	62.9	38
**2.13**	-	51	521	5.5	^{1}(M+X)LCT	94	548	125.9	38
**2.14**	2.64	66	535	6.43	^{3}(M+X)LCT	-	535	37.56(8K)	39
**2.15**	3.07	83	538	6.98	^{1}(M+X)LCT	-	538	109.81(8K)	39
**2.16**	-	90.1	462	9.33	-	-	505	79	40
	-		540	133 500	-	-	-	-	
**2.17**	2.878	37	577	7.9	$^{1/3}$(M+X)LCT	71	592	65	41
**2.18**	2.883	53	545	8.8	$^{1/3}$(M+X)LCT	89	567	110	41
**2.19**	2.7694	81	539	6.5	$^{1/3}$(M+X)LCT	92	552	32	41
**2.20**	-	9	616	1.2	$^{1/3}$(M+X)LCT	14	626	30	41
**2.21**	-	33	583	2.5	$^{1/3}$(M+X)LCT	56	584	29	41
**2.22**	2.693	13	565	1.7	$^{1/3}$(M+X)LCT	67	575	17.4	41
**2.23**	-	11	660	2	$^{1/3}$(M+X)LCT	24	668	42	41
**2.24**	2.7204	38	636	3.3	$^{1/3}$(M+X)LCT	59	645	22	41
**2.25a-H**	2.7617	74	549	6.3	^{1}(M+X)LCT	-	-	40	42
**2.25b-H**	2.766	83	531	6.5	^{1}(M+X)LCT	-	-	44.2	42
**2.25a-OMe**	2.7318	45	567	6.5	^{1}(M+X)LCT	-	-	50.7	42

 This journal is © The Royal Society of Chemistry 20xx

Please do not adjust margins

Please do not adjust margins

**2.25b-OMe**	2.7128	61	556	8.7	^{1}(M+X)LCT	-	-	56.4	42
**2.25a-Furyl**	2.6835	67	524	6.8	^{1}(M+X)LCT	-	-	34.9	42
**2.25a-F**	2.7067	89	529	7.3	^{1}(M+X)LCT	-	-	56.5	42
**2.25b-F**	2.7189	85	520	6.9	^{1}(M+X)LCT	-	-	59.1	42
**2.26a-H**	2.7276	87	530	6.1	^{1}(M+X)LCT	-	-	72.8	42
**2.26b-H**	2.7859	76	538	7.3	^{1}(M+X)LCT	-	-	36.8	42
**2.27b-H**	-	59	543	7.7	^{1}(M+X)LCT	-	-	58.7	42
**2.27b-F**	-	70	540	9.6	^{1}(M+X)LCT	-	-	51.6	42
**2.28a-H**	2.783	68	552	4.4	^{1}(M+X)LCT	-	-	24.2	42
**2.28b-H**	2.7682	68	520	5.5	^{1}(M+X)LCT	-	-	26	42
**2.28a-F**		62	538	7.6	^{1}(M+X)LCT	-	-	45.9	42
**2.29a-H**	2.7218	61	561	7.3	^{1}(M+X)LCT	-	-	26.7	42
**2.30**	-	74.2	445	15.6	$^{1/3}$(M+X)LCT	86.7	461	119.3	43
**2.31**	2.6626	36.9	585	5.85	^{1}(M+X)LCT	-	-	-	44
**2.32**	2.6594	43	565	7.99	^{3}(M+X)LCT	-	-	-	44
**2.33**	2.6783	34	586	5.99	^{3}(M+X)LCT	-	-	-	44
**2.34**	2.778	49	553	4.42	^{3}(M+X)LCT	-	-	-	45
**2.35**	3.471	48	443	19.2	^{1}(M+X)LCT	-	454	577	46
**2.36**	3.133	<0.01	417,574	0.0057	^{1}(M+X)LCT	-	465, 626	2.1	46
**2.37**	3.072	<0.01	534	0.0093	^{1}(M+X)LCT	-	422, 552	0.00048	46
**2.38**	3.053	11	548	1.1	^{1}(M+X)LCT	-	551	112	46
**2.39**	3.05	5	570	0.4	^{1}(M+X)LCT	-	576	202	46
**2.40**	3.014	<0.01	595	0.0044	^{1}(M+X)LCT	-	582	1.7	46

This journal is © The Royal Society of Chemistry 20xx

Please do not adjust margins

Please do not adjust margins

**2.41G**	2.7	43	518	11.5	^{1}(M+X+L)LCT	-	528	92.6	47
**2.41Y**	3.189	18	550	11.6	^{1}(M+X+L)LCT	-	535	81.8	47
**2.42**	2.97	<0.1	660	10.1	^{3}XLCT	-	-	-	48
**2.43 (Form I)**	2.86	3	629	1.3	^{3}XLCT	-	-	-	48
**2.43 (Form II)**	2.65	8	617	2.2	^{3}XLCT	-	-	-	48
**2.44**	2.77	18	602	5.3	^{3}XLCT	-	-	-	48
**2.45**	-	92	485	8.3	$^{1/3}$MLCT	-	-	-	49
**2.46**	3.6941	55	550	14.5	$^{1/3}$(M+X)LCT	100	560	36	50
**2.47**	3.681	53	530	18.3	$^{1/3}$(M+X)LCT	92	540	48	50
**2.48**	3.7949	51	520	20	$^{1/3}$(M+X)LCT	90	530	91	50
**2.49**	-	50	530	9	$^{1/3}$(M+X)LCT	90	535	32	50
**2.50**	-	20	510	3.5	$^{1/3}$(M+X)LCT	51	520	37	50
**2.51**	-	22	500	2	$^{1/3}$(M+X)LCT	84	505	35	50
**2.52**	-	2	465	4	$^{1/3}$(M+X)LCT	/	470	42	50
**2.53**	-	38	535	5	$^{1/3}$(M+X)LCT	-	-	-	51
**2.54**	2.513	82	518	14.2	^{3}MLCT	-	-	16.5	52

 This journal is © The Royal Society of Chemistry 20xx

Please do not adjust margins

Table S4: Summary of the photophysical properties of Cu_3L_3 complexes.

			RT					LT		
**Complex**	**Physical State**	**Inter $d_{Cu\cdots Cu}$ (Å)**	**Φ (%)**	**λ_{em} (nm)**	**τ_{av}**	**Origin**	**λ_{em} (nm)**	**τ_{av}**	**Origin**	**Ref**
**3.1**	Crystal	4.919-5.322	-	684.5	-	^{3}MC	-	-	-	53
**3.2**	Crystal	> 5.000	-	-	-	-	460	-	LC	54
**3.3**	Crystal	3.0526	65	650	27.9 µs	^{3}MLCT	425, 452, 576	770.2 µs	^{3}LC	55
				-	-	-	654	27.68 µs	^{3}MLCT	
**3.4**	Crystal	2.824 - 3.218	1.3	655	5.8 µs	^{1,3}MLCT	688	7.1 µs	^{1,3}MLCT	55
**3.5**	Crystal	3.368 - 3.666	24	663	21 µs	^{3}MC	-	-	-	56
**3.6**	Liquid Crystal	-	42	661	28 µs	^{3}MM	-	-	-	56
**3.7**	Liquid Crystal	-	-	664	22 µs	^{3}MM	-	-	-	56
	Crystal	-	24	664	26 µs	^{3}MM	-	-	-	
**3.8**	Crystal	3.746	-	540	17.89 µs	^{3}MLCT	545	-	^{3}MLCT	57
**3.9**	Crystal	3.094	-	583	11.40 µs	^{3}MLCT	593	-	^{3}MLCT	57
**3.10**	THF	-	0.7	431	2.2 ns	^{1}LC	-	-	-	58
	Crystal	3.366 - 3.506	6.4	467	0.53 ns	^{1}LC	461	0.99 ns	^{1}LC	
**3.11**	DMSO	-	22	518	2.75 ns	^{1}LC/^{1}MSLC	-	-	-	59
		-	-	-	50.9 ns	^{3}LC/^{3}MSLC	-	-	-	
**3.12**	THF[a]	-	-	-	-	-	-	-	-	60
	THF/90% H_2O	-	>99.9	672	38 µs	^{3}LMMCT/^{3}LC	-	-	-	
	PMMA	-	>99.9	674	9.02 µs	^{3}LMMCT/^{3}LC	-	-		
	Crystal	2.976	>99.9	677	35.7 µs	^{3}LMMCT/^{3}LC	-	-	-	
**3.13**	$CHCl_3$	-	-	387	2.09 ns	^{1}ILCT	371, 424, 446	1.19 ns	^{1}ILCT	61
	Crystal	-	-	384	1.39 ns	-	393, 415, 429	2.20 ns	-	
**3.14**	$CHCl_3$	-	-	387	0.91 ns	^{1}ILCT	374, 427, 434	0.58 ns	^{1}ILCT	61
	Crystal	3.007	-	704	16,1 µs	-	713	38,9 µs	-	
**3.15**	Crystal	> 5.000	-	575	56.0 µs	-	592	63.6 µs	-	62
**3.16**	Crystal	> 5.000	-	590	48.4 µs	-	590	68.7 µs	-	62
**3.17**	Crystal	-	-	-	-	-	570	63.8 µs	^{3}ML'CT	63
**3.18**	Crystal	3.661	1.5	570	0.72 ns	^{1}LC (An located)	520	-	^{1}LC (An located)	64
**3.19**	Crystal	2.953	4.6	450	1.03 ns	^{1}MMCT	450	1.69 ns	^{1}MMCT	64
		-	-	650	14.9 µs	^{3}LC (An located)	675	879 µs	^{3}LC (An located)	
**3.20**	DCM	-	-	527	0.22 µs /0.55 µs[a]	^{3}MLCT+^{3}LLCT	535	-	^{3}MLCT+^{3}LLCT	65
	Crystal	-	72	513	60 µs	^{3}MC	557	203 µs	^{3}MC	
**3.21**	DCM	-	-	525	0.47 µs/1.22 µs[a]	^{3}MLCT+^{3}LLCT	542	-	^{3}MLCT+^{3}LLCT	65
	Crystal	-	39	500	52 µs	^{3}MC	555	257 µs	^{3}MC	
**3.22**	DCM	-	-	528	0.13 µs/0.24 µs[a]	^{3}MLCT+^{3}LLCT	542	-	^{3}MLCT+^{3}LLCT	65

Please do not adjust margins

	Crystal	-	58	515	23 µs	^{3}MC	565	215 µs	^{3}MC	
**3.23**	DCM	-	-	533	0.19 µs/0.60 µs[a]	^{3}MLCT+^{3}LLCT	540	-	^{3}MLCT+^{3}LLCT	65
	Crystal	-	72	520	18 µs	^{3}MC	570	129 µs	^{3}MC	
**3.24**	DCM	-	-	537	0.05 µs/0.11 µs[a]	^{3}MLCT+^{3}LLCT	559	-	^{3}MLCT+^{3}LLCT	65
	Crystal	-	74	551	42 µs	^{3}MC	551	142.2 µs	^{3}MC	
**3.25**	DCM	-	-	533	0.06 µs/0.36 µs[a]	^{3}MLCT+^{3}LLCT	545	-	^{3}MLCT+^{3}LLCT	65
	Crystal	-	58	585	15 µs	^{3}MC	585	116.5 µs	^{3}MC	
**3.26**	Crystal	3.727	99.9	626	25.13 µs	-	631	36.33 µs	-	66
**3.27**	Crystal	5.432	69.4	596	36.10 µs	-	450	36.33 µs	-	66
		-	-	-	-	-	573	49.10 µs	-	

[a]In oxygen free conditions

This journal is © The Royal Society of Chemistry 20xx
Please do not adjust margins

Please do not adjust margins

Table S5: Summary of the photophysical properties of Cu_3X_3 complexes

			RT				LT			
**Complex**	**Physical State**	**$d_{Cu\cdots Cu}$ intra (Å)**	**Φ (%)**	**λ_{em} (nm)**	**τ_{av}**	**Origin**	**λ_{em} (nm)**	**τ_{av}**	**Origin**	**Ref**
**3.28**	Crystal	2.48	56	475	15 µs	^{3}(M+X)LCT	495	43.3 µs	^{3}(M+X)LCT	67
			-	-	-	-	580	20 µs	^{3}CC	
**3.29**	Crystal	2.56	100	453	13.4 µs	^{3}(M+X)LCT	461	65.8 µs	^{3}(M+X)LCT	67
			-	-	-	-	580	13 µs	^{3}CC	
**3.30**	Crystal	2.57	50	442	11.0 µs	^{3}(M+X)LCT	462	48 µs	^{3}(M+X)LCT	67
			-	613	5 µs	^{3}CC	622	10 µs	^{3}CC	
	Ground	-	22	505	16.5 µs	^{3}(M+X)LCT	450	50 µs	^{3}(M+X)LCT	
			-	-	-	-	505	128 µs	^{3}(M+X)LCT	
			-	-	-	-	610	11 µs	^{3}CC	
	Melted	-	30	505	19.8 µs	^{3}(M+X)LCT	505	170 µs	^{3}(M+X)LCT	
			-	614	5.5 µs	^{3}CC	-	-	-	
**3.31**	ACN		-	508	-	MLCT	-	-	-	68
	Solid		-	628	0.24 µs	^{3}MLCT	-	-	-	
**3.32**	ACN		-	398	-	-	-	-	-	68
	Crystal	2.53 - 2.56	-	674	5.09 µs	^{3}MLCT	-	-	-	
**3.33**	Crystal	2.8622 - 3.5479	68	488	447 µs	^{3}MLCT	-	-	-	69
**3.34**	Crystal	2.567	31	590	30.2 µs	^{3}MLCT	590	16.3 µs	^{3}MLCT	52

Please do not adjust margins

Please do not adjust margins

Table S6: Summary of the photophysical properties of Cu_4 complexes in the solid state.

		RT				LT (77K)			
**Complex**	**d_{Cu-Cu} (Å)**	**Φ (%)**	**λ_{em} (nm)**	**τ_{av} (µs)**	**Origin**	**λ_{em} (nm)**	**τ_{av} (µs)**	**Origin**	Ref
**4.1bu**	2.677 - 2.749	91.8	463	10	^{3}CC	474	-	^{3}CC	70
**4.1gn**	2.625 - 2.810	90.6	485	14.2	^{3}CC	-	-	-	70
**4.1ye**	2.627 - 2.702	80.1	460, 580	11.4	^{3}CC	-	-	-	70
**4.1wh**	2.642 - 2.804	95.2	475, 588	12	^{3}CC	599	-	^{3}CC	70
**4.2**	2.629 - 2.739	52.8 (R), 59,7 (S)	630	15.4 (R), 14,9 (S)	^{3}CC	-	-	-	71
**4.3**	2.65 - 2.76	94.8 (R), 93.2 (S)	610	11.8 (S)	^{3}CC	-	-	-	72
**4.4**	2.59 - 2.67	78.7 (R), 83.8 (S)	646	15.6 (S)	^{3}CC	-	-	-	72
**4.5**	-	>85	573	9.18	^{3}(M+X)LCT	-	-	-	73
**4.6**	-	>85	623	17.32	^{3}(M+X)LCT	-	-	-	73
**4.7**	2.707	94.9	584	20.36	^{3}CC	644[a]	-	^{3}CC	74
**4.8**	2.630 - 2.704	68	598	6.35	^{3}CC	622	-	^{3}CC	75
**4.9**	2.6276 - 2.7525	-	418	<0.0003	^{3}(M+X)LCT	-	-	-	76
			520	0.31; 2.09	^{3}CC	-	-	-	
**4.10**	2.6693 - 2.7864	-	422	<0.0003	^{3}(M+X)LCT	-	-	-	76
			580	0.32; 2.19	^{3}CC	-	-	-	
**4.11**	2.6424 - 2.7207	-	581	0.32; 2.14	^{3}CC	-	-	-	76
**4.12**	2.6678 - 2.7635	-	578	0.33; 2.27	^{3}CC	-	-	-	76
**4.13**	2.6594 - 2.7473	-	554	0.32; 2.18	^{3}CC	-	-	-	76
**4.14**	2.437	44	526	13.2	^{3}MLCT	-	16.3	^{3}MLCT	52
**4.15**	2.696 - 2.797	1	495	0.015	^{3}(M+X)LCT	495	-	^{3}(M+X)LCT	77
**4.16**	2.702 - 2.744	23	597	0.46	^{3}(M+X)LCT	455	-	^{3}(M+X)LCT	77
**4.17**	2.666 - 2.816	1	550	1.57	^{3}CC	565	-	^{3}CC	77
**4.18**	2.495 - 2.747	1	517	4.12	^{3}(M+X)LCT	494	-	^{3}(M+X)LCT	77

 This journal is © The Royal Society of Chemistry 20xx

Please do not adjust margins

Please do not adjust margins

**4.19**	2.54 - 2.76	58	529	5	^{3}CC	519[b]	24	-	78
**4.20**	2.67 - 2.78	93	593	16	^{3}CC	479[b]	19.6	^{3}MLCT	78
**4.21**	-	65	600	23	^{3}CC	497[b]	-	^{3}MLCT	78
**4.22**	2.78 - 2.92	33	510	10	^{3}CC	505[b]	-	^{3}MLCT	78
**4.23**	2.81 - 2.91	38	567[d]	1.9	^{3}(M+X)LCT + ILCT	-	-	-	79
**4.24**	2.158	9	610	1.1	^{3}CC + ^{3}MLCT	-	-	-	80
**4.25**	2.592 - 2.929	4.9	517	-	^{3}CC	-	-	-	36
**4.26c**	2.54; 2.712	-	492	4.5	^{3}(M+X)LCT	-	-	-	81
**4.26s**	2.5653; 2.8050	-	680	7.1	^{3}(M+X)LCT	494	-	^{3}(M+X)LCT	81
**4.27**	2.97 - 2.99[d]<br>3.40-4.86[e]	94	559[c]	20.9	^{3}CC	-	-	-	82
**4.28**	2.88-2.94[d]<br>3.71-4.44[e]	61	513[c]	17.7	^{3}CC	-	-	-	82
**4.29**	2.87-2.92[d]<br>3.01-4.94[e]	99	458[c]	16.2	^{3}CC	-	-	-	82
**4.30**	2.87-2.91[d]<br>2.99-4.94[e]	8	457[c]	2.6	^{3}CC + ^{3}MLCT	-	-	-	82
**4.31**	2.88-2.94[d]<br>3.71-4.44[e]	82	457[c]	15.6	^{3}CC	-	-	-	82
**4.32**	2.82-2.89[d]<br>3.81-4.08[e]	42	519	27.6	^{3}CC	532	29.8	^{3}CC	83
			450	26.9	^{3}CC	450	29.4	^{3}CC	
**4.33**	3.33 - 3.37	-	380; 426	-	^{1}LC	350	63	^{1}LC	84
			525	-	^{3}MLCT	525	66	^{3}MLCT	
**4.34**	3.33 - 3.37	-	360	-	^{1}LC	387	141	^{1}LC	84
			400	-		510	974	^{3}MLCT	

[a]Measurements performed at 80 K, [b]measurements performed at 10 K, [c]measurements performed on thin films, [d] adjacent Cu atoms, [e] diagonal Cu atoms

Please do not adjust margins

Table S7: Summary of photophysical properties of mechanochromic compounds

Complex	T (K)	Before grinding			After grinding		Proposed mechanism	Ref
		$d_{Cu\cdots Cu}$ (Å)	λ_{em} (nm)	Φ (%)	λ_{em} (nm)	Φ (%)		
**1.34**	298	9.392	472	59	550	34	Crystal-to-amorphous phase transition	85
**1.35*R*/*S***	295	-	564	22	579	55	Disruption of the intermolecular C–H···π interactions	86
	77	-	-	-	580	39		
**1.36*R*/*S***	295	-	549	25	606	53	Disruption of the intermolecular C–H···π interactions	86
	77	-	538	8	603	39		
**1.37**	295	-	496	43.5	546	26.2	Crystal-to-amorphous phase transition *via* disruption of C-H···π hydrogen bonds	87
**1.38**	295	-	524	32.6	560	20.6	Crystal-to-amorphous phase transition *via* disruption of C-H···π hydrogen bonds	87
**1.39**	295	-	503	24.5	534	7.5	Crystal-to-amorphous phase transition *via* disruption of C-H···π hydrogen bonds	87
**1.40**	295	-	542	20.0	557	9.8	Crystal-to-amorphous phase transition *via* disruption of C-H···π hydrogen bonds	87
**1.41**	298	-	544	58.8	596	7	Disruption of intermolecular interactions, in particular hydrogen bonds	88
	77	-	603	98.1	-	-		
**1.42**	298	-	528	70.3	600	34	Disruption of intermolecular interactions, in particular hydrogen bonds	88
	77	-	555	≈ 100	-	-		
**1.43**	298	-	522	29.1	605	16	Disruption of intermolecular interactions, in particular hydrogen bonds	88
	77	-	541	77.4	-	-		
**2.9**	298	2.9904	507	28.2	551		Crystal-to-amorphous phase transition	
**2.55**	298	-	556	66.8	577	-	Crystal-to-amorphous phase transition associated with uptake/release of $CHCl_3$ guest in the porous structure	89
**2.56**	298	-	702	0.92	612	5.20	Release of CH_3CN guest	90
**2.57**	298	-	685	2.00	612	9.28	Release of CH_3CN guest	90
**2.58**	298	-	652	12.03	612	24.30	Release of CH_3CN guest	90
**3.35a**	298		374, 413, 435[a]<br>675[b]	8.3	-	-	Pressure-induced phosphorescence enhancement (PIPE)	91
	77	2.854, 2.909[c,d]<br>3.211, 3.196, 3.255[c,e]	-	28.9	-	-		
**3.35b**	298	3.756, 3.942[d]<br>3.17, 3.18, 3.25[e]	375, 415, 440[a]<br>635[b]	33.1	Red-shift under pressure	-	Cz/Cz excimer-based luminescence	91
	77	-	-	54.9	-	-		
**3.36**	298	3.88[d]<br>3.22[e]	675	2.66/2.67[f]	675	-	Pressure-induced phosphorescence enhancement (PIPE)	92

[a] λ_{ex} 305 nm; [b] λ_{ex} 280 nm; [c] Measured at 100K; [d] Inter-trimeric $d_{Cu\cdots Cu}$ distances; [e] Intra-trimeric $d_{Cu\cdots Cu}$ distances; [f] Calculated.

1. Bibliography

1. R. Hamze, S. Shi, S. C. Kapper, D. S. Muthiah Ravinson, L. Estergreen, M. C. Jung, A. C. Tadle, R. Haiges, P. I. Djurovich, J. L. Peltier, R. Jazzar, G. Bertrand, S. E. Bradforth and M. E. Thompson, "Quick-Silver" from a Systematic Study of Highly Luminescent, Two-Coordinate, d^{10} Coinage Metal Complexes, *J. Am. Chem. Soc.*, 2019, **141**, 8616–8626.
2. J. P. Zobel, A. M. Wernbacher and L. Gonzalez, Efficient Reverse Intersystem Crossing in Carbene-Copper-Amide TADF Emitters via an Intermediate Triplet State, *Angew. Chem. Int. Ed.*, 2023, **62**, e202217620.
3. C. N. Muniz, C. A. Archer, J. S. Applebaum, A. Alagaratnam, J. Schaab, P. I. Djurovich and M. E. Thompson, Two-Coordinate Coinage Metal Complexes as Solar Photosensitizers, *J. Am. Chem. Soc.*, 2023, **145**, 13846–13857.
4. R. Li, A. Ying, Y. Tan, Y. Ai and S. Gong, Efficient Blue Photo- and Electroluminescence from CF_3-Decorated Cu(I) Complexes, *Chem. Eur. J.*, 2024, **30**, e202400817.
5. A. Ying, Y. Tan and S. Gong, Highly Efficient Copper(I) Emitters Supported by Secondary Metal-Ligand Interactions, *Adv. Opt. Mater.*, 2024, **12**, 2303333.
6. H. J. Wang, Y. Liu, B. Yu, S. Q. Song, Y. X. Zheng, K. Liu, P. Chen, H. Wang, J. Jiang and T. Y. Li, A Configurationally Confined Thermally Activated Delayed Fluorescent Two-Coordinate Cu(I) Complex for Efficient Blue Electroluminescence, *Angew. Chem. Int. Ed.*, 2023, **62**, e202217195.
7. A. Ying, Y. H. Huang, C. H. Lu, Z. Chen, W. K. Lee, X. Zeng, T. Chen, X. Cao, C. C. Wu, S. Gong and C. Yang, High-Efficiency Red Electroluminescence Based on a Carbene-Cu(I)-Acridine Complex, *ACS Appl. Mater. Interfaces*, 2021, **13**, 13478–13486.
8. A. Ying, L. Zhan, Y. Tan, X. Cao, C. Yang and S. Gong, Copper(I) complexes with planar chirality realize efficient circularly polarized electroluminescence, *Sci. China Chem.*, 2023, **66**, 2274–2282.
9. T.-y. Li, J. Schaab, P. I. Djurovich and M. E. Thompson, Toward rational design of TADF two-coordinate coinage metal complexes: understanding the relationship between natural transition orbital overlap and photophysical properties, *J. Mater. Chem. C*, 2022, **10**, 4674–4683.
10. C. N. Muniz, J. Schaab, A. Razgoniaev, P. I. Djurovich and M. E. Thompson, π-Extended Ligands in Two-Coordinate Coinage Metal Complexes, *J. Am. Chem. Soc.*, 2022, **144**, 17916–17928.
11. J. Li, L. Wang, Z. Zhao, X. Li, X. Yu, P. Huo, Q. Jin, Z. Liu, Z. Bian and C. Huang, Two-Coordinate Copper(I)/NHC Complexes: Dual Emission Properties and Ultralong Room-Temperature Phosphorescence, *Angew. Chem. Int. Ed.*, 2020, **59**, 8210–8217.
12. M. Ghosh, J. Chatterjee, P. Panwaria, A. Kudlu, S. Tothadi and S. Khan, Silylene-Copper-Amide Emitters: From Thermally Activated Delayed Fluorescence to Dual Emission, *Angew. Chem. Int. Ed.*, 2024, **63**, e202410792.
13. R. Hamze, M. Idris, D. S. Muthiah Ravinson, M. C. Jung, R. Haiges, P. I. Djurovich and M. E. Thompson, Highly Efficient Deep Blue Luminescence of 2-Coordinate Coinage Metal Complexes Bearing Bulky NHC Benzimidazolyl Carbene, *Front. Chem.*, 2020, **8**, 401.
14. A. M. T. Muthig, J. Wieland, C. Lenczyk, S. Koop, J. Tessarolo, G. H. Clever, B. Hupp and A. Steffen, Towards Fast Circularly Polarized Luminescence in 2-Coordinate Chiral Mechanochromic Copper(I) Carbene Complexes, *Chem. Eur. J.*, 2023, **29**, e202300946.
15. Q. Zhang, N. Li, X. Wan, X. F. Song, Y. Zhang, H. Liu, J. Miao, Y. Zou, C. Yang and K. Li, Harnessing of Cooperative Cu⋯H Interactions for Luminescent Low-Coordinate Copper(I) Complexes towards Stable OLEDs, *Angew. Chem. Int. Ed.*, 2025, **64**, e202419290.
16. R. Tang, S. Xu, T. L. Lam, G. Cheng, L. Du, Q. Wan, J. Yang, F. F. Hung, K. H. Low, D. L. Phillips and C. M. Che, Highly Robust Cu^{I}-TADF Emitters for Vacuum-Deposited OLEDs with Luminance up to 222 200 cd m^{-2} and Device Lifetimes (LT_{90}) up to 1300 hours at an Initial Luminance of 1000 cd m^{-2}, *Angew. Chem. Int. Ed.*, 2022, **61**, e202203982.
17. Q. Gu, F. Chotard, J. Eng, A. M. Reponen, I. J. Vitorica-Yrezabal, A. W. Woodward, T. J. Penfold, D. Credgington, M. Bochmann and A. S. Romanov, Excited-State Lifetime Modulation by Twisted and Tilted Molecular Design in Carbene-Metal-Amide Photoemitters, *Chem. Mater.*, 2022, **34**, 7526–7542.
18. A. S. Romanov, S. T. E. Jones, Q. Gu, P. J. Conaghan, B. H. Drummond, J. Feng, F. Chotard, L. Buizza, M. Foley, M. Linnolahti, D. Credgington and M. Bochmann, Carbene metal amide photoemitters: tailoring conformationally flexible amides for full color range emissions including white-emitting OLED, *Chem. Sci.*, 2020, **11**, 435–446.
19. F. Chotard, V. Sivchik, M. Linnolahti, M. Bochmann and A. S. Romanov, Mono- versus Bicyclic Carbene Metal Amide Photoemitters: Which Design Leads to the Best Performance?, *Chem. Mater.*, 2020, **32**, 6114–6122.
20. C. Riley, W. Jones, N. L. Phuoc, M. Linnolahti and A. S. Romanov, Cyclic(amino)(barrelene)carbene metal amide complexes: Synthesis and thermally activated delayed fluorescence, *Org. Electron.*, 2025, **137**, 107156.
21. M. Gernert, L. Balles-Wolf, F. Kerner, U. Muller, A. Schmiedel, M. Holzapfel, C. M. Marian, J. Pflaum, C. Lambert and A. Steffen, Cyclic (Amino)(aryl)carbenes Enter the Field of Chromophore Ligands: Expanded pi System Leads to Unusually Deep Red Emitting Cu(I) Compounds, *J. Am. Chem. Soc.*, 2020, **142**, 8897–8909.
22. L. Cao, S. Huang, W. Liu, H. Zhao, X. G. Xiong, J. P. Zhang, L. M. Fu and X. Yan, Thermally Activated Delayed Fluorescence from d^{10}-Metal Carbene Complexes through Intermolecular Charge Transfer and Multicolor Emission with a Monomer-Dimer Equilibrium, *Chem. Eur. J.*, 2020, **26**, 17222–17229.
23. A. Ying, Y. Ai, C. Yang and S. Gong, Aggregation-Dependent Circularly Polarized Luminescence and Thermally Activated Delayed Fluorescence from Chiral Carbene-Cu(I)-Amide Enantiomers, *Angew. Chem. Int. Ed.*, 2022, **61**, e202210490.
24. W. Yang, W. Wang, M. Cao, N. Gao, C. Liu, J. Zhang, Z. Peng, C. Du and B. Zhang, Efficiently luminescent cuprous iodide complexes supported by novel N^P-chelating ligands: Synthesis, structure and optoelectronic performances, *Dyes Pigm.*, 2020, **180**, 108487.
25. B. Zhang, J. Zhang, A. Sun, C. Liu, M. Gu, Y. Chen, B. Wei and C. Du, Efficiently luminescent mononuclear copper iodide complexes with sterically hindered iminephosphine chelating ligands, *New J. Chem.*, 2021, **45**, 8763–8768.
26. M. Klein, N. Rau, M. Wende, J. Sundermeyer, G. Cheng, C.-M. Che, A. Schinabeck and H. Yersin, Cu(I) and Ag(I)

Please do not adjust margins

Complexes with a New Type of Rigid Tridentate N,P,P-Ligand for Thermally Activated Delayed Fluorescence and OLEDs with High External Quantum Efficiency, *Chem. Mater.*, 2020, **32**, 10365–10382.
27. X. Cheng, C. Liu, W. Lu, J. Xiao, C. Du, M. Yin and B. Zhang, Tetrazolate-based neutral Cu(I) complex for efficient and short-lived blue thermally activated delayed fluorescence, *Polyhedron*, 2024, **258**, 117029.
28. C. Sun, L. Llanos, P. Arce, A. Oliver, R. Wannemacher, J. Cabanillas-Gonzalez, L. Lemus and D. Aravena, Nuclearity Control for Efficient Thermally Activated Delayed Fluorescence in a CuI Complex and its Halogen-Bridged Dimer, *Chem. Mater.*, 2021, **33**, 6383–6393.
29. G. Farias, C. A. M. Salla, J. Toigo, L. Duarte, A. J. Bortoluzzi, E. Girotto, H. Gallardo, T. D. Z. Atvars, B. de Souza and I. H. Bechtold, Enhancing the phosphorescence decay pathway of Cu(I) emitters - the role of copper-iodide moiety, *Dalton Trans.*, 2022, **51**, 1008–1018.
30. S. V. Skvortsova, N. A. Shekhovtsov, M. I. Rakhmanova, D. G. Samsonenko, E. B. Nikolaenkova and M. B. Bushuev, Copper(I) complexes with a 4-(1*H*-pyrazol-1-yl)-2-(pyridin-2-yl)pyrimidine ligand: Synthesis, structures and theoretical insights into the tuning of emission properties, *Inorg. Chem. Commun.*, 2025, **174**, 114048.
31. X. Pan, Z.-X. Li, G. Wang, Y. Yang, X. Xin, H. Han, J.-M. Liu, Q.-H. Jin and D. Yan, Excellent Blue Emissive Neutral Cu(I) Complexes: Structural Analysis, Thermochromic Luminescent Properties, and Terahertz Spectrum Research, *Cryst. Growth Des.*, 2021, **21**, 6425–6436.
32. Y. Zhu, X. Kuang, T. T. Li, C. L. Hou, H. Yang and C. Z. Lu, Synthesis and Characterization of Copper(I) Halide Heteroleptic Complexes with Thermally Activated Delayed Fluorescence, *Inorg. Chem.*, 2025, **64**, 8334–8342.
33. Y. Zhao, T. Nakae, K. Segawa, M. Yoshida, M. Kato, K. Omoto, S. Ito, T. Yamada and Y. Yamanoi, Structural and Photophysical Differences in Crystalline Trigonal Planar Copper Iodide Complexes with 1,2-Bis(methylpyridin-2-yl)disilane Ligands, *Inorg. Chem.*, 2024, **63**, 22361–22371.
34. C. Xu, L. Lv, Z. Zhang and W. Liu, Inorganic–Organic Hybrid Molecular Clusters with Cu_2I_2 Rhomboid Dimer Core as Light-Emitting Coating Materials, *J. Cluster Sci.*, 2021, **32**, 1205–1211.
35. S. Lin, Z. Ma, X. Ji, W. Chu, Q. Zhou, Y. Liu and Z. Shi, Efficient non-doped cluster light-emitting diodes based on semiconducting copper iodide hybrids, *J. Lumin.*, 2024, **271**.
36. J. Soldevila-Sanmartín, E. Ruiz, D. Choquesillo-Lazarte, M. E. Light, C. Viñas, F. Teixidor, R. Núñez, J. Pons and J. G. Planas, Tuning the architectures and luminescence properties of Cu(I) compounds of phenyl and carboranyl pyrazoles: the impact of 2D versus 3D aromatic moieties in the ligand backbone, *J. Mater. Chem. C*, 2021, **9**, 7643–7657.
37. A. Gusev, M. Kiskin, E. Braga, E. Zamnius, M. Kryukova, N. Karaush-Karmazin, G. Baryshnikov, B. Minaev and W. Linert, Structure and emission properties of dinuclear copper(I) complexes with pyridyltriazole, *RSC Adv.*, 2023, **13**, 3899–3909.
38. A. Gusev, E. Braga, E. Zamnius, K. Zakharov, M. Kiskin and W. Linert, 0D and 1D-dimensional Cu(I)-based halides pyridyltriazoles basis: Synthesis, Structures, and photophysical properties, *Inorg. Chim. Acta*, 2024, **568**.
39. J. Chatterjee, A. Chatterjee, R. Tanwar, P. Panwaria, S. Saikia, M. D. Ambhore, P. Mandal and P. Hazra, Activation of TADF in Photon Upconverting Crystals of Dinuclear Cu(I)-Iodide Complexes by Ligand Engineering, *J. Phys. Chem. Lett.*, 2024, **15**, 6069–6080.
40. Y. N. Zhao, Q. Yang, B. H. Yao, R. Y. Cao, H. Zhang, S. L. Wei, D. H. Wei, K. Li, Y. B. Si and S. Q. Zang, Afterglow Copper(I) Iodine Cluster Scintillator, *Angew. Chem. Int. Ed.*, 2025, **64**, e202500481.
41. T. Hofbeck, T. A. Niehaus, M. Fleck, U. Monkowius and H. Yersin, P intersectionN Bridged Cu(I) Dimers Featuring Both TADF and Phosphorescence. From Overview towards Detailed Case Study of the Excited Singlet and Triplet States, *Molecules*, 2021, **26**.
42. K. Xu, B. L. Chen, F. Yang, L. Liu, X. X. Zhong, L. Wang, X. J. Zhu, F. B. Li, W. Y. Wong and H. M. Qin, Largely Color-Tuning Prompt and Delayed Fluorescence: Dinuclear Cu(I) Halide Complexes with tert-Amines and Phosphines, *Inorg. Chem.*, 2021, **60**, 4841–4851.
43. J. Jiang, Y. Zhao, Z. Li, Y. Ye, Z. Wu, F. Jiang, L. Chen and M. Hong, Copper(I) Halide Complex Featuring Blue Thermally Activated Delayed Fluorescence and Aggregate Induced Emission for Efficient X-ray Scintillation and Imaging, *Angew. Chem. Int. Ed.*, 2025, **64**, e202422995.
44. M. Cao, Y. Zhao, M. Gu, C. Liu, Q. Zhu, Y. Chen, B. Wei, C. Du and B. Zhang, Syntheses, Crystal Structures and Photophysical Properties of Dinuclear Copper(I) Complexes Bearing Diphenylphosphino-Substituted Benzimidazole Ligands, *ChemistrySelect*, 2021, **6**, 2156–2163.
45. H. Qi, C. Liu, Q. Geng, M. Liu, M. Gu, C. Du and B. Zhang, A combined experimental and theoretical investigations of structure and luminescence in mono− and di−nuclear copper(I) complexes containing N^P−type iminephosphine ligand, *J. Organomet. Chem.*, 2025, **1036**.
46. J. M. Busch, D. S. Koshelev, A. A. Vashchenko, O. Fuhr, M. Nieger, V. V. Utochnikova and S. Brase, Various Structural Design Modifications: para-Substituted Diphenylphosphinopyridine Bridged Cu(I) Complexes in Organic Light-Emitting Diodes, *Inorg. Chem.*, 2021, **60**, 2315–2332.
47. X. W. Zhang, C. H. Huang, M. Yang, X. L. Chen and C. Z. Lu, Rotational isomerization: spontaneous structural transformation of a thermally activated delayed fluorescence binuclear copper(I) complex, *Dalton Trans.*, 2023, **52**, 9893–9898.
48. S. V. Skvortsova, F. K. Verkhov, E. B. Nikolaenkova, M. I. Rakhmanova, T. E. Kokina, T. S. Sukhikh, N. A. Shekhovtsov and M. B. Bushuev, Interplay of the Cucdots, three dots, centeredCu distance and coordination geometry as a factor affecting the quantum efficiency in dimeric copper(I) halide complexes with derivatives of 4-pyrazolylpyrimidine-2-thiol, *Dalton Trans.*, 2025, **54**, 9000–9015.
49. G. Cheng, D. Zhou, U. Monkowius and H. Yersin, Fabrication of a Solution-Processed White Light Emitting Diode Containing a Single Dimeric Copper(I) Emitter Featuring Combined TADF and Phosphorescence, *Micromachines (Basel)*, 2021, **12**.
50. A. Y. Baranov, A. S. Berezin, D. G. Samsonenko, A. S. Mazur, P. M. Tolstoy, V. F. Plyusnin, I. E. Kolesnikov and A. V. Artem'ev, New Cu(I) halide complexes showing TADF combined with room temperature phosphorescence: the

 This journal is © The Royal Society of Chemistry 20xx

Please do not adjust margins

Please do not adjust margins

balance tuned by halogens, *Dalton Trans.*, 2020, **49**, 3155–3163.
51. Y. V. Demyanov, I. Y. Bagryanskaya, M. I. Rakhmanova and A. V. Artem’ev, Bi- and tetranuclear Cu(I) complexes based on tris(6-methyl-2-pyridyl)phosphine: Synthesis and photophysical study, *Inorg. Chem. Commun.*, 2024, **169**, 113062.
52. Y.-Y. Yang, R.-C. Hao, Q.-Q. Wu, P. Luo, J. Xu, X.-Y. Dong and C.-X. Zhang, Structural regulation of NHC-protected copper(I) clusters through substitution for photoluminescence enhancement, *Inorg. Chem. Front.*, 2024, **11**, 4757–4769.
53. K. Fujisawa, M. Saotome, S. Takeda and D. J. Young, Structures and Photoluminescence of Coinage Metal(I) Phenylpyrazolato Trinuclear Complexes [M(3,5-Et_2-4-Ph-pz)]$_3$ and Arene Sandwich Complexes {[Ag(3,5-Et_2-4-Ph-pz)]$_3$}$_2$(Ar) (Ar = Mesitylene and Toluene), *Chem. Lett.*, 2020, **49**, 670–673.
54. J. S. Lakhi, M. R. Patterson and H. V. R. Dias, Coinage metal metallacycles involving a fluorinated 3,5-diarylpyrazolate, *New J. Chem.*, 2020, **44**, 14814–14822.
55. L.-R. Xing, Z. Lu, M. Li, J. Zheng and D. Li, Revealing High-Lying Intersystem Crossing in Brightly Luminescent Cyclic Trinuclear CuI/AgI Complexes, *J. Phys. Chem. Lett.*, 2020, **11**, 2067–2073.
56. R. Giménez, O. Crespo, B. Diosdado and A. Elduque, Liquid crystalline copper(i) complexes with bright room temperature phosphorescence, *J. Mater. Chem. C*, 2020, **8**, 6552–6557.
57. S.-Z. Zhan, W. Chen, J. Zheng, S. W. Ng and D. Li, Luminescent polymorphic aggregates of trinuclear Cu(I)–pyrazolate tuned by intertrimeric Cu⋯NPy weak coordination bonds, *Dalton Trans.*, 2021, **50**, 1733–1739.
58. H. Li, J. Luo, Z.-Y. Zhang, R.-J. Wei, M. Xie, Y.-L. Huang, G.-H. Ning and D. Li, Cyclic Trinuclear Copper(I) Complex Exhibiting Aggregation-Induced Emission: A Novel Fluorescent Probe for the Selective Detection of Gold(III) Ions, *Inorg. Chem.*, 2022, **61**, 414–421.
59. R.-Q. Xia, J. Zheng, R.-J. Wei, J. He, D.-Q. Ye, M.-D. Li, G.-H. Ning and D. Li, Strong visible light-absorbing BODIPY-based Cu(I) cyclic trinuclear sensitizer for photocatalysis, *Inorg. Chem. Front.*, 2022, **9**, 2928–2937.
60. H. Yang, J. Zheng, M. Xie, D. Luo, W.-J. Tang, S.-K. Peng, G. Cheng, X. Zhang, X.-P. Zhou, C.-M. Che and D. Li, Aggregation-Enhanced Emission in a Red Cu(I) Emitter with Quantum Yield >99%, *ACS Mater. Lett.*, 2022, **4**, 1921–1928.
61. M. Vanga, B. T. Diroll, Á. R. Muñoz-Castro and H. V. R. Dias, Filling the gap with a bulky diaryl boron group: fluorinated and non-fluorinated copper pyrazolates fitted with a dimesityl boron moiety on the backbone, *Dalton Trans.*, 2023, **52**, 16356–16363.
62. C. V. Hettiarachchi, M. A. Rawashdeh-Omary, D. Korir, J. Kohistani, M. Yousufuddin and H. V. R. Dias, Trinuclear Copper(I) and Silver(I) Adducts of 4-Chloro-3,5-bis(trifluoromethyl)pyrazolate and 4-Bromo-3,5-bis(trifluoromethyl)pyrazolate, *Inorg. Chem.*, 2013, **52**, 13576–13583.
63. Z. Lu, M. Vanga, S. Li, J. O. Adebanjo, M. R. Patterson, H. V. R. Dias and M. A. Omary, Relativistic modulation of supramolecular halogen/copper interactions and phosphorescence in Cu(I) pyrazolate cyclotrimers, *Dalton Trans.*, 2023, **52**, 3964–3970.
64. Z.-M. Xiao, J.-X. Yang, X. Chen, W.-J. Tang, S.-K. Peng, D.-B. Hao, Z.-P. Zhao, J. Zheng and D. Li, A fluorescence–phosphorescence dual-emissive Cu_3(pyrazolate)$_3$ complex with highly tunable emission colours for anticounterfeiting and temperature sensing, *Inorg. Chem. Front.*, 2024, **11**, 1808–1818.
65. K. F. Baranova, A. A. Titov, J. R. Shakirova, V. A. Baigildin, A. F. Smol’yakov, D. A. Valyaev, G.-H. Ning, O. A. Filippov, S. P. Tunik and E. S. Shubina, Substituents’ Effect on the Photophysics of Trinuclear Copper(I) and Silver(I) Pyrazolate–Phosphine Cages, *Inorg. Chem.*, 2024, **63**, 16610–16621.
66. G.-Q. Huang, R.-Q. Xia, X. Chen, H. Yang, Y.-L. Huang, K. Wu, J. Zheng, W. Lu and D. Li, Enabling Thermally Stimulated Delayed Phosphorescence in Cu(I) Cyclic Trinuclear Complexes with Near-Unity Quantum Yield, *J. Am. Chem. Soc.*, 2025, **147**, 6415–6426.
67. A. Y. Baranov, E. A. Pritchina, A. S. Berezin, D. G. Samsonenko, V. P. Fedin, N. A. Belogorlova, N. P. Gritsan and A. V. Artem'ev, Beyond Classical Coordination Chemistry: The First Case of a Triply Bridging Phosphine Ligand, *Angew. Chem. Int. Ed.*, 2021, **60**, 12577–12584.
68. J. Yang, P. Shang, H. Yuan, L. Song, Q. Huang, Z. Jiang, C. Wu and X. Jiang, Regulated Adaptive Self-Assembly of Cu_3I_3 Supramolecular Clusters and their Photocatalytic Properties, *Cryst. Growth Des.*, 2022, **22**, 4926–4934.
69. B. Kaur, R. Gourkhede and M. S. Balakrishna, Luminescence Behavior of Cationic and Neutral Cu^I Complexes of Phosphine and Pyridine Embedded 1,2,3-Triazole, *Inorg. Chem.*, 2024, **63**, 16981–16990.
70. J. Chen, S. Geng, X. Zhang, X. Pan, C. Chen, R. Li, J. Wen, C. Sun, R. Chen, Z. Xiao and L. Mao, Isomeric Zero-Dimensional Hybrid Copper(I) Iodides as Highly Efficient Multicolor Phosphors, *ACS Mater. Lett.*, 2024, **6**, 865–876.
71. L. Yao, G. Niu, J. Li, L. Gao, X. Luo, B. Xia, Y. Liu, P. Du, D. Li, C. Chen, Y. Zheng, Z. Xiao and J. Tang, Circularly Polarized Luminescence from Chiral Tetranuclear Copper(I) Iodide Clusters, *J. Phys. Chem. Lett.*, 2020, **11**, 1255–1260.
72. X. Ji, Y. Liu, R. Li, Z. Zhang, X. Zhang, C. Chen, J. Chen, H. Lu, R. Chen and L. Mao, Mono- and Bi-Dentate Chiral Ligands Lead to Efficient Circularly Polarized Luminescence in 0D and 3D Semiconducting Copper(I) Iodides, *Adv. Opt. Mater.*, 2023, **11**.
73. Y. Zhou, T. He, P. Yuan, J. Yin, S. Chen, L. Gutiérrez-Arzaluz, L. Wang, O. M. Bakr and O. F. Mohammed, Colloidal Cu_4I_4 Clusters for High-Resolution X-ray Imaging Scintillation Screens, *ACS Mater. Lett.*, 2023, **5**, 2002–2008.
74. Q. Hu, C. Zhang, X. Wu, G. Liang, L. Wang, X. Niu, Z. Wang, W. D. Si, Y. Han, R. Huang, J. Xiao and D. Sun, Highly Effective Hybrid Copper(I) Iodide Cluster Emitter with Negative Thermal Quenched Phosphorescence for X-Ray Imaging, *Angew. Chem. Int. Ed.*, 2023, **62**, e202217784.
75. L. Wang, H. Sun, C. Sun, D. Xu, J. Tao, T. Wei, Z. H. Zhang, Y. Zhang, Z. Wang and W. Bi, Lead-free, stable orange-red-emitting hybrid copper based organic-inorganic compounds, *Dalton Trans.*, 2021, **50**, 2766–2773.
76. Y. N. Toikka, Y. N. Toikka, A. S. Mereshchenko, G. L. Starova and N. A. Bokach, Synthesis, Structure, and Luminescent Properties of Copper(I) Iodide Clusters Bearing

Please do not adjust margins

Please do not adjust margins

Dialkylcyanamide Ligands, *Russ. J. Gen. Chem.*, 2022, **92**, 1467–1474.
77. M. F. Galimova, E. M. Zueva, M. M. Petrova, A. B. Dobrynin, I. E. Kolesnikov, E. I. Musina, R. R. Musin, A. A. Karasik and O. G. Sinyashin, Design of luminescent complexes with different Cu_4I_4 cores based on pyridyl phenoxarsines, *Dalton Trans.*, 2024, **53**, 1087–1098.
78. P. Boden, P. Di Martino-Fumo, J. M. Busch, F. R. Rehak, S. Steiger, O. Fuhr, M. Nieger, D. Volz, W. Klopper, S. Brase and M. Gerhards, Investigation of Luminescent Triplet States in Tetranuclear Cu(I) Complexes: Thermochromism and Structural Characterization, *Chem. Eur. J.*, 2021, **27**, 5439–5452.
79. X. Lu, S.-J. Wu, Y.-S. Wang, S.-Y. Wei, L. Meng, X.-H. Huang, X.-L. Chen and C.-Z. Lu, Efficient doped and non-doped light-emitting diodes based on a TADF-emitting Cu_4Br_4 cluster, *Inorg. Chem. Front.*, 2024, **11**, 2775–2783.
80. S. Wang, E. E. Morgan, S. Panuganti, L. Mao, P. Vishnoi, G. Wu, Q. Liu, M. G. Kanatzidis, R. D. Schaller and R. Seshadri, Ligand Control of Structural Diversity in Luminescent Hybrid Copper(I) Iodides, *Chem. Mater.*, 2022, **34**, 3206–3216.
81. I. Strelnik, A. Shamsieva, K. Akhmadgaleev, T. Gerasimova, I. Dayanova, I. Kolesnikov, R. Fayzullin, D. Islamov, E. Musina, A. Karasik and O. Sinyashin, Emission and Luminescent Vapochromism Control of Octahedral Cu4I4 Complexes by Conformationally Restricted P,N Ligands, *Chem. Eur. J.*, 2023, **29**, e202202864.
82. Y. Watanabe, B. M. Washer, M. Zeller, S. Savikhin, L. V. Slipchenko and A. Wei, Copper(I)–Pyrazolate Complexes as Solid-State Phosphors: Deep-Blue Emission through a Remote Steric Effect, *J. Am. Chem. Soc.*, 2022, **144**, 10186–10192.
83. S. K. Rajagopal, M. Zeller, S. Savikhin, L. V. Slipchenko and A. Wei, Rigidochromism of tetranuclear Cu(I)-pyrazolate macrocycles: steric crowding with trifluoromethyl groups, *Chem. Commun.*, 2024, **60**, 11307–11310.
84. G. B. Yakovlev, A. A. Titov, A. F. Smol'yakov, A. Y. Chernyadyev, O. A. Filippov and E. S. Shubina, Tetranuclear Copper(I) and Silver(I) Pyrazolate Adducts with 1,1'-Dimethyl-2,2'-bibenzimidazole: Influence of Structure on Photophysics, *Molecules*, 2023, **28**.
85. X. Yu, X. Li, Z. Cai, L. Sun, C. Wang, H. Rao, C. Wei, Z. Bian, Q. Jin and Z. Liu, Mechanochromic properties in a mononuclear Cu(I) complex without cuprophilic interactions, *Chem. Commun.*, 2021, **57**, 5082–5085.
86. A. M. T. Muthig, O. Mrozek, T. Ferschke, M. Rodel, B. Ewald, J. Kuhnt, C. Lenczyk, J. Pflaum and A. Steffen, Mechano-Stimulus and Environment-Dependent Circularly Polarized TADF in Chiral Copper(I) Complexes and Their Application in OLEDs, *J. Am. Chem. Soc.*, 2023, **145**, 4438–4449.
87. M. Yan, W. Lu, B. Zhang, C. Liu, X. Zi, J. Zhang, C. Qi, M. Liu and C. Du, Mononuclear copper(I) complexes with mechanochromic thermally activated delayed fluorescence behaviour based on switchable hydrogen bonds, *Polyhedron*, 2023, **237**.
88. A. Gusev, E. Braga, E. Zamnius, M. Kiskin, A. Ali, G. Baryshnikov and W. Linert, Mononuclear copper(I) complexes bearing a 3-phenyl-5-(pyridin-4-yl)-1,2,4-triazole ligand: synthesis, crystal structure, TADF-luminescence, and mechanochromic effects, *Dalton Trans.*, 2023, **52**, 14995–15008.
89. W. Lu, Y. Song, C. Liu, J. Zhang, B. Zhang and C. Du, Highly phosphorescent dinuclear Cu(I) complexes with iminephosphine tetradentate chelating ligand (PNNP): Structures and luminescence properties, *Inorg. Chem. Commun.*, 2021, **134**.
90. W.-T. Chen, C.-H. Li, Z.-Y. Liang, Z.-L. Zhang, D.-Q. Liu, J.-W. Ye, L. Chen and X.-M. Chen, Large and Tunable Wavelength Blue Shifts in Luminescent Piezochromism of Cu(I) Complexes via a Guest Encapsulation Strategy, *ACS Mater. Lett.*, 2024, **6**, 2077–2084.
91. M. Xie, X. R. Chen, K. Wu, Z. Lu, K. Wang, N. Li, R. J. Wei, S. Z. Zhan, G. H. Ning, B. Zou and D. Li, Pressure-induced phosphorescence enhancement and piezochromism of a carbazole-based cyclic trinuclear Cu(I) complex, *Chem. Sci.*, 2021, **12**, 4425–4431.
92. Z. Lu, C. M. Archambault, S. Li, U. Syed, S. Wang, A. Kumar, G. Shen, Z. Liu, M. A. Omary and H. Yan, Modulating the Extent of Anisotropic Cuprophilicity via High Pressure with Piezochromic Luminescence Sensitization, *J. Phys. Chem. Lett.*, 2023, **14**, 508–515.

 This journal is © The Royal Society of Chemistry 20xx

Please do not adjust margins
